# Locus-specific ChIP combined with NGS analysis reveals genomic regulatory regions that physically interact with the *Pax5* promoter in a chicken B cell line

**DOI:** 10.1093/dnares/dsx023

**Published:** 2017-06-06

**Authors:** Toshitsugu Fujita, Fusako Kitaura, Miyuki Yuno, Yutaka Suzuki, Sumio Sugano, Hodaka Fujii

**Affiliations:** 1 Department of Biochemistry and Genome Biology, Hirosaki University Graduate School of Medicine, Hirosaki, Aomori 036-8562, Japan; 2 Chromatin Biochemistry Research Group, Combined Program on Microbiology and Immunology, Research Institute for Microbial Diseases, Osaka University, Suita, Osaka 565-0871, Japan; 3 Department of Medical Genome Sciences, Graduate School of Frontier Sciences, The University of Tokyo, Kashiwa, Chiba 277-8562, Japan; 4 Department of Medical Genome Sciences, Graduate School of Frontier Sciences, The University of Tokyo, Minato-ku, Tokyo 108-8639, Japan

**Keywords:** chromosomal interaction, ChIP, iChIP, *in vitro* enChIP, Pax5

## Abstract

Chromosomal interactions regulate genome functions, such as transcription, via dynamic chromosomal organization in the nucleus. In this study, we attempted to identify genomic regions that physically bind to the promoter region of the *Pax5* gene, which encodes a master regulator for B cell lineage commitment, in a chicken B cell line, DT40, with the goal of obtaining mechanistic insight into transcriptional regulation through chromosomal interaction. We found that the *Pax5* promoter bound to multiple genomic regions using locus-specific chromatin immunoprecipitation (locus-specific ChIP), a method for locus-specific isolation of target genomic regions, in combination with next-generation sequencing (NGS). Comparing chromosomal interactions in wild-type DT40 with those in a macrophage-like counterpart, we found that some of the identified chromosomal interactions were organized in a B cell-specific manner. In addition, deletion of a B cell-specific interacting genomic region in chromosome 11, which was marked by active enhancer histone modifications, resulted in moderate but significant down-regulation of *Pax5* transcription. Together, these results suggested that *Pax5* transcription in DT40 is regulated by B cell-specific inter-chromosomal interactions. Moreover, these analyses showed that locus-specific ChIP combined with NGS analysis is useful for non-biased identification of functional genomic regions that physically interact with a locus of interest.

## 1. Introduction

Elucidation of the molecular mechanisms underlying genome functions, such as transcription, requires identification of molecules that interact with the genomic regions of interest. To this end, several biochemical methods have been developed. For example, proteomics of isolated chromatin (PICh) utilizes oligonucleotide probes to capture target loci for identification of associated proteins.^1^ We developed locus-specific chromatin immunoprecipitation (locus-specific ChIP) technologies [see review^2–4^]. By combining locus-specific ChIP with downstream biochemical analyses, one can identify molecules that physically interact with target genomic regions in cells in a locus-specific manner.

In principle, locus-specific ChIP consists of locus-tagging and affinity purification. On the basis of various strategies for locus-tagging, we developed two locus-specific ChIP technologies, insertional ChIP (iChIP)^5^^,^^6^ and engineered DNA-binding molecule-mediated ChIP (enChIP).^7^^,^^8^ iChIP utilizes an exogenous DNA-binding protein, such as a bacterial protein LexA, and its binding element for locus-tagging, whereas enChIP employs engineered DNA-binding molecules, such as transcription activator-like (TAL) proteins^9^^,^^10^ and the clustered regularly interspaced short palindromic repeats (CRISPR) system,^11^^,^^12^ for the same purpose. After isolation of tagged loci by affinity purification, their interacting molecules can be comprehensively identified by downstream analyses including mass spectrometry (MS), next-generation sequencing (NGS), and microarrays. In fact, we have successfully identified proteins that interact with target loci by iChIP or enChIP in combination with MS, including a quantitative form of MS, stable isotope labeling with amino acids in cell culture (SILAC) (iChIP/enChIP-MS or -SILAC).^7^^,^^8^^,^^13–15^ In addition, identification of chromatin-binding RNAs is also feasible using enChIP in combination with RT-PCR (enChIP-RT-PCR) or RNA sequencing (enChIP-RNA-Seq).^8^^,^^16^ Locus-specific ChIP has been used by other researchers.^17^^,^^18^ Several years after our initial publications of iChIP, essentially identical methods have been reported by other groups.^19–21^ In addition, after our initial publication of enChIP, a method essentially identical with enChIP using a TAL protein has been reported.^22^

Genome functions are mediated by chromosomal interactions (e.g. interactions between enhancers and promoters). To detect physical chromosomal interactions, several techniques have been utilized to date, including fluorescence *in situ* hybridization (FISH)^23^ and chromosome conformation capture (3C) plus 3C-derived methods.^24–28^ In this regard, locus-specific ChIP can also be applied to detection of physical chromosomal interactions (one-to-many interactions). In fact, using iChIP in combination with microarrays (iChIP-microarray), McCullagh et al. succeeded in non-biased identification of genomic regions that interact with a target locus in yeast.^17^ More recently, we showed that it is also feasible to analyze physical chromosomal interactions using enChIP combined with NGS analysis (enChIP-Seq).^29^

The *Pax5* gene encodes a transcription factor essential for B cell lineage commitment.^30^ Disruption of the *Pax5* gene inhibits B cell differentiation,^31^^,^^32^ and *Pax5*-deficient B cells can be trans-differentiated into other lymphoid cell types in mice.^33–35^ To obtain mechanistic insight into transcriptional regulation of the *Pax5* gene, we previously used iChIP-SILAC to identify proteins that interact with the *Pax5* promoter region in the chicken B cell line DT40.^15^ However, the mechanisms underlying regulation of *Pax5* transcription by chromosomal interactions remain incompletely understood. Although intron 5 of the mouse *Pax5* gene contains enhancers essential for transcription of the gene,^36^ it remains unclear whether similar regulatory mechanisms exist across species. In this regard, because the DNA sequences of *Pax5* intron 5 are scarcely conserved between mouse and chicken, it is possible that transcription of *Pax5* is controlled in a species-specific manner.

In this study, we applied iChIP in combination with NGS analysis (iChIP-Seq) to direct identification of genomic regions that interact with the *Pax5* promoter region in DT40 cells. Some of the detected chromosomal interactions were independently confirmed by an updated form of enChIP-Seq. In addition, deletion of a B cell-specific interacting genomic region significantly decreased *Pax5* transcription in DT40 cells, suggesting that the deleted region is an enhancer and that *Pax5* transcription is regulated through chromosomal interactions between this enhancer and the promoter in a B cell-specific manner. Thus, locus-specific ChIP in combination with NGS analysis revealed a mechanism of transcriptional regulation of the chicken *Pax5* gene.

## 2. Materials and methods

### 2.1. Cell culture

DT40, Non-KI(B), KI(B), and KI(MΦ) were maintained as described previously.^15^

### 2.2. iChIP-Seq, *in vitro* enChIP-Seq, and bioinformatics analysis

Non-KI(B), KI(B), and KI(MΦ) (2 × 10^7^ each) were subjected to the iChIP procedure as described previously.^15^ DT40 was subjected to the *in vitro* enChIP procedure as described previously.^37^ The complex of CRISPR RNA (crRNA) targeting the *Pax5* promoter and trans-activating crRNA (tracrRNA) was used as Pax5 gRNA for *in vitro* enChIP. The gRNA sequences are shown in Supplementary Table S1. Briefly, after fragmentation of chromatin DNA (the average length of fragments was about 2 kbp), the target region was isolated by iChIP or *in vitro* enChIP. After purification of DNA, DNA libraries were prepared using TruSeq ChIP Sample Prep Kit (Illumina); in this preparation step, DNA fragments around 0.4 kbp in length were selectively concentrated. The libraries were subjected to DNA sequencing using the HiSeq platform according to the manufacturer’s protocol. NGS and data analysis were performed as described previously.^38^^,^^39^ Additional information on NGS analysis is provided in Supplementary Table S2. NGS data were mapped onto the reference genome galGal4 using ELAND (Illumina). Narrow peaks of each iChIP-Seq dataset (see Steps 1 and 2 in Fig. 2) were detected using Model-based Analysis of ChIP-Seq 2 (MACS2, http://liulab.dfci.harvard.edu/MACS/ (14 May 2017, date last accessed)) with default parameters. Images of NGS peaks were generated using Integrative Genomics Veiwer (IGV) (http://software.broadinstitute.org/software/igv/ (14 May 2017, date last accessed)). The accession number of the NGS data is DRA005236.

### 2.3. Plasmids

The Cas9 expression plasmid (Addgene #41815)^40^ and chimeric single guide RNA (sgRNA) expression plasmid (Addgene #41824)^40^ were provided by Dr. George Church through Addgene. For construction of the sgRNA expression plasmids, double-stranded DNA (dsDNA) encoding the target sequences were cloned downstream of the U6 promoter in the sgRNA expression plasmid. Alternatively, DNA fragments coding the U6 promoter, target sequence, gRNA scaffold, and termination signal were synthesized and cloned in plasmids by GeneArt gene synthesis services (Thermo Fisher Scientific).

### 2.4. Deletion of genomic loci by CRISPR-mediated genome editing

DT40 cells (1 × 10^7^) were transfected with a Cas9 expression plasmid (120 µg), sgRNA expression plasmids (120 µg) targeting each end of a target genomic region, and pEGFP-N3 (0.3 µg, Clontech) by electroporation using Gene Pulser II (Bio-Rad) at 250 V and 950 µF. One day later, GFP-positive cells were sorted and expanded individually. To confirm targeted locus deletion, genomic DNA was extracted and subjected to genotyping PCR with KOD FX (Toyobo). PCR cycles were as follows: heating at 94 °C for 2 min followed by 30 cycles of 98 °C for 10 s, 60 °C for 30 s, and 68 °C for 1 min. Primers used for genotyping PCR are shown in Supplementary Table S1.

### 2.5. RNA extraction and quantitative RT-PCR

Extraction of total RNA and quantitative RT-PCR were performed as described previously.^41^ Primers used in this experiment are shown in Supplementary Table S1.

### 2.6. ChIP assays

Antibodies against H3K4me1 (39298, Active Motif), H3K27ac (39134, Active Motif), and histone H3 (MABI0301, Wako) were used. ChIP assays were performed with DT40 cells (2 × 10^6^) and each antibody (3.5 µl for H3K4me1 or 2 µg for the others) as described previously.^13^ DNA purified using ChIP DNA Clean & Concentrator (Zymo Research) was used as template for real-time PCR with SYBR Select Master Mix (Applied Biosystems) on Applied Biosystems 7900HT Fast Real-Time PCR System. Primers used in this experiment are shown in Supplementary Table S1.

## 3. Results and discussion

### 3.1. Scheme of iChIP-Seq for analysis of chromosomal interactions around the *Pax5* promoter region

**Figure 1.**
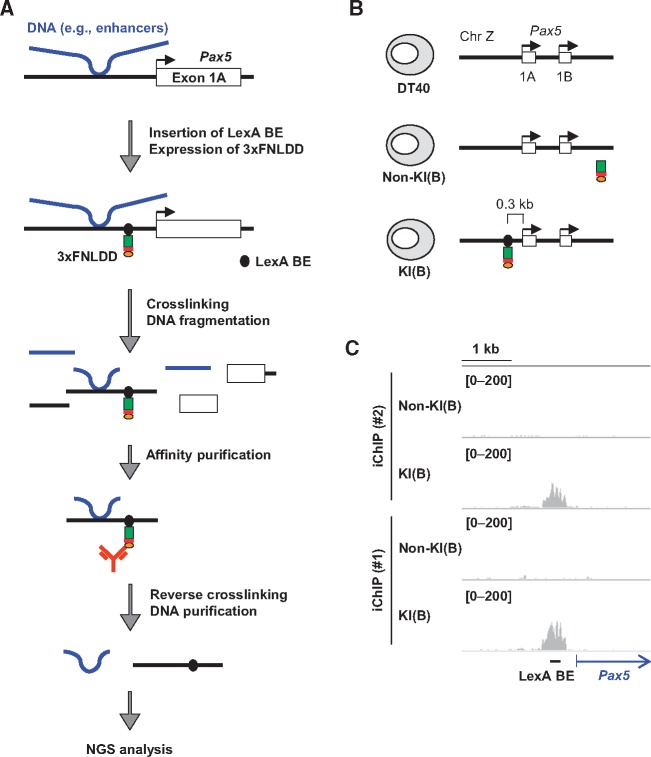
iChIP-Seq for identification of genomic regions that physically interact with the *Pax5* promoter in chicken B cells. (**A**) A schematic diagram of iChIP-Seq in this study. First, LexA-binding elements (LexA BE) were inserted into the *Pax5* promoter region, and 3xFNLDD [a fusion protein of the 3xFLAG-tag, a nuclear localization signal (NLS), and LexA DNA-binding domain plus dimerization domain] was expressed in DT40. After crosslinking with formaldehyde, chromatin DNA was fragmented by sonication, and the target locus was affinity-purified with anti-FLAG antibody. After reversal of crosslinking, DNA was purified and subjected to NGS analysis. (**B**) The chicken B cell line DT40 and its derivatives used for iChIP-Seq. Non-KI(B): DT40 expressing 3xFNLDD, KI(B): DT40 containing LexA BE in the *Pax5* promoter region and expressing 3xFNLDD. The LexA BE was inserted 0.3 kb upstream from the transcription start site of *Pax5* exon 1A. KI: Knock-In. (**C**) Images of NGS peaks around the *Pax5* promoter region. NGS data from iChIP-Seq were visualized in IGV. The vertical viewing range (*y*-axis shown as scale) was set to 0–200 based on the magnitude of the noise peaks.

The scheme of iChIP-Seq used in this study is as follows (Fig. 1A): (I) Using homologous recombination, binding elements of the bacterial DNA-binding protein LexA (LexA BE) were inserted ∼0.3 kb upstream from the transcription start site (TSS) of the *Pax5* exon 1A in chromosome Z of DT40 cells. (II) 3xFNLDD, which consists of 3xFLAG-tag, a nuclear localization signal (NLS), and LexA DNA-binding and dimerization domains, was expressed in the cells established in Step (I). (III) The resultant cells were crosslinked with formaldehyde and lysed, and chromatin DNA was fragmented by sonication. (IV) The tagged locus (the *Pax5* promoter region) was affinity-purified using an anti-FLAG antibody. (V) After reverse crosslinking and DNA purification, genomic regions interacting with the *Pax5* promoter region were identified by NGS analysis.

In this study, we utilized DT40-derived cell lines (Fig. 1B), which were previously established for iChIP-SILAC analysis of the *Pax5* promoter region;^15^ Non-KI(B) is DT40 expressing 3xFNLDD, and KI(B) is a DT40-derived cell line harboring an insertion of LexA BE in the *Pax5* promoter region and expressing 3xFNLDD. In our previous study, insertion of LexA BE and expression of 3xFNLDD did not disturb transcription of the endogenous *Pax5* gene,^15^ suggesting that the regulatory machinery involved for *Pax5* transcription is retained in both Non-KI(B) and KI(B). In addition, we previously showed that the *Pax5* promoter region can be efficiently isolated from KI(B) by iChIP (∼10% of input as DNA yields).^15^ Following the experimental scheme (Fig. 1A), we isolated the *Pax5* promoter region by iChIP and subjected the purified DNA samples to NGS analysis using HiSeq. NGS reads corresponding to the *Pax5* promoter region were clearly enriched when iChIP was performed with KI(B) but not Non-KI(B) [iChIP(#1) in Fig. 1C]. A biological replicate of the iChIP-Seq analysis showed similar results [iChIP(#2) in Fig. 1C]. These results demonstrated efficient isolation of the *Pax5* promoter region by iChIP.

### 3.2. Detection of genomic regions that physically interact with the *Pax5* promoter region in DT40

**Figure 2.**
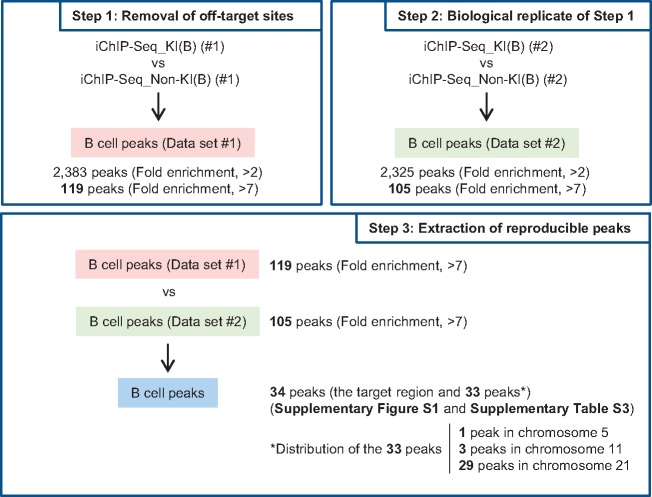
Extraction of genomic regions interacting with the *Pax5* promoter region in DT40. (**Step 1**) Removal of off-target binding sites. iChIP-Seq data were compared between KI(B) and Non-KI(B) (negative control) using Model-based Analysis of ChIP-Seq (MACS) to eliminate off-target binding sites. (**Step 2**) Analysis of another biological replicate of Step 1. (**Step 3**) Identification of genomic regions that interact with the *Pax5* promoter region. The genomic regions commonly detected in Steps 1 and 2 represent candidate genomic regions that physically interact with the *Pax5* promoter region.

Next, we proceeded to identify the genomic regions that interact with the *Pax5* promoter region in DT40 (Fig. 2). Because 3xFNLDD might interact with endogenous DNA sequences, similar to the recognition sequence of LexA (CTGTN_8_ACAG)^42^ in the DT40 genome, iChIP-Seq data obtained from Non-KI(B) were used to eliminate genomic regions detected due to such off-target binding (Step 1 in Fig. 2). We identified 2,383 peak positions with read numbers more than 2-fold higher in KI(B) than in Non-KI(B), and considered these as potential interacting genomic regions. Because the top 5% peaks (119 peaks) had >7-fold enrichment (Step 1 in Fig. 2), we arbitrarily set 7-fold as the threshold for extraction of genomic regions that interact with the *Pax5* promoter region with high frequency. As shown in Step 2 in Figure 2, 105 peaks passed this criterion (>7-fold), from among 2,325 peaks (>2-fold) in the biological replicate. Comparing the 119 (Step 1) and 105 (Step 2) peaks, we identified 34 peaks as reproducibly passing the criterion (Step 3 in Fig. 2, Supplementary Fig. S1, and Supplementary Table S3). In this regard, 90 out of the 119 peaks (75.6%) in Data set #1 (>7-fold) were detected in the 2,325 peaks in Data set #2 (>2-fold), and 62 out of the 105 peaks (59.0%) in Data set #2 (>7-fold) were detected in the 2,383 peaks in Data set #1 (>2-fold) (Supplementary Fig. S1). Therefore, more than about 60% of the peaks that passed the criterion ‘>7-fold’ were reproducibly detected.

Among the 34 peaks, 1 peak was the LexA BE-inserted *Pax5* promoter region (Supplementary Table S3) and the other 33 were considered as candidate genomic regions that physically interact with the *Pax5* promoter region. *Pax5* intron 5 was not detected as the candidates (Supplementary Table S3), although intron 5 of the mouse *Pax5* gene contains enhancers essential for transcription of the gene.^36^ In this study, we filtered iChIP-Seq data on the basis of the criterion ‘>7-fold’ to extract genomic regions that bind to the *Pax5* promoter region with high frequency. In this regard, more permissive criteria would increase the number of potentially interacting genomic regions. In fact, the criterion ‘more than 2-fold’ extracted 680 common peaks between the 2,383 peaks (Step 1) and 2,325 peaks (Step 2). However, in this case, it might be more difficult to confidently evaluate whether the detected peaks reflect physiological interactions or noise. Therefore, hereafter we focused on the 33 peaks passing the more stringent criterion.

### 3.3. Confirmation of physical chromosomal interactions by *in vitro* enChIP

**Figure 3.**
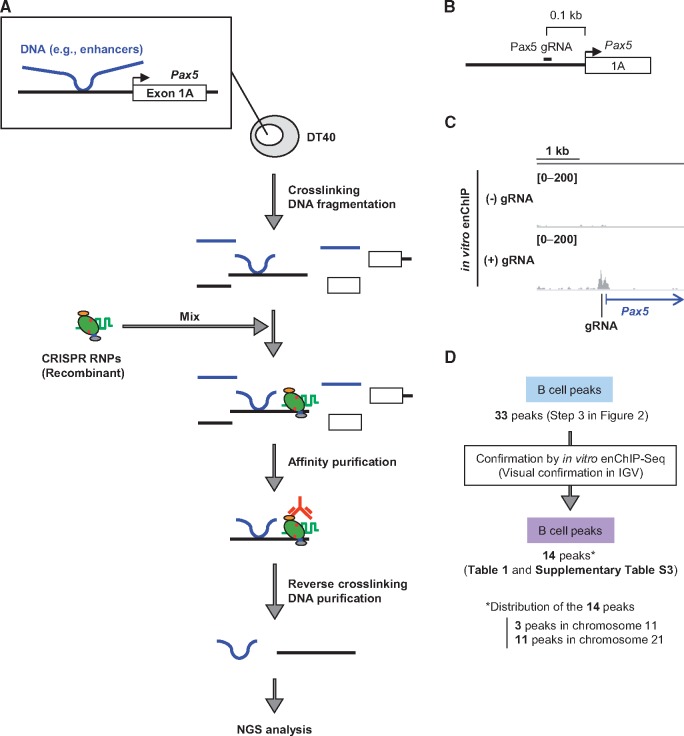
*In vitro* enChIP-Seq for confirmation of the results of iChIP-Seq. (**A**) Intact DT40 cells were crosslinked with formaldehyde, and chromatin DNA was fragmented by sonication. Recombinant CRISPR ribonucleoproteins (RNPs), which consist of 3xFLAG-dCas9-Dock and gRNA targeting the *Pax5* promoter region, were mixed with the fragmented chromatin DNA to capture the target region. After affinity purification with anti-FLAG antibody, reversal of crosslinking, and DNA purification, the DNA was subjected to NGS analysis. (**B**) Target position of Pax5 gRNA. (**C**) NGS peak images around the *Pax5* promoter region. NGS data from *in vitro* enChIP-Seq were visualized in IGV. The vertical viewing range (*y*-axis shown as scale) was set at 0-200 based on the magnitude of the noise peaks. (**D**) Confirmation of the results of iChIP-Seq. The peak positions identified by iChIP-Seq were confirmed by *in vitro* enChIP-Seq in IGV.

The chromosomal interactions identified by iChIP-Seq (Fig. 2) could include artificial ones caused by insertion of LexA BE. Therefore, it was necessary to confirm the identified chromosomal interactions by another independent method in intact DT40 cells. To this end, we attempted to utilize *in vitro* enChIP, an updated form of conventional enChIP.^37^^,^^43^ In *in vitro* enChIP, recombinant molecules [e.g. recombinant CRISPR ribonucleoproteins (RNPs)] are used for *in vitro* locus-tagging rather than in cell locus-tagging (Fig. 3A). Because intact cells can be utilized in this *in vitro* system, it is unnecessary to consider disruption of physiological chromosomal conformation and potential side-effects caused by in cell locus-tagging. In *in vitro* enChIP using CRISPR RNPs (Fig. 3A), chromosomal conformation in intact DT40 was fixed by formaldehyde crosslinking, and chromatin DNA was fragmented by sonication. The *Pax5* promoter was captured by CRISPR RNPs and isolated from a mixture of the fragmented chromatin by affinity purification. NGS analysis of the isolated material then revealed the genomic regions that physically interact with the *Pax5* promoter.

**Figure 4.**
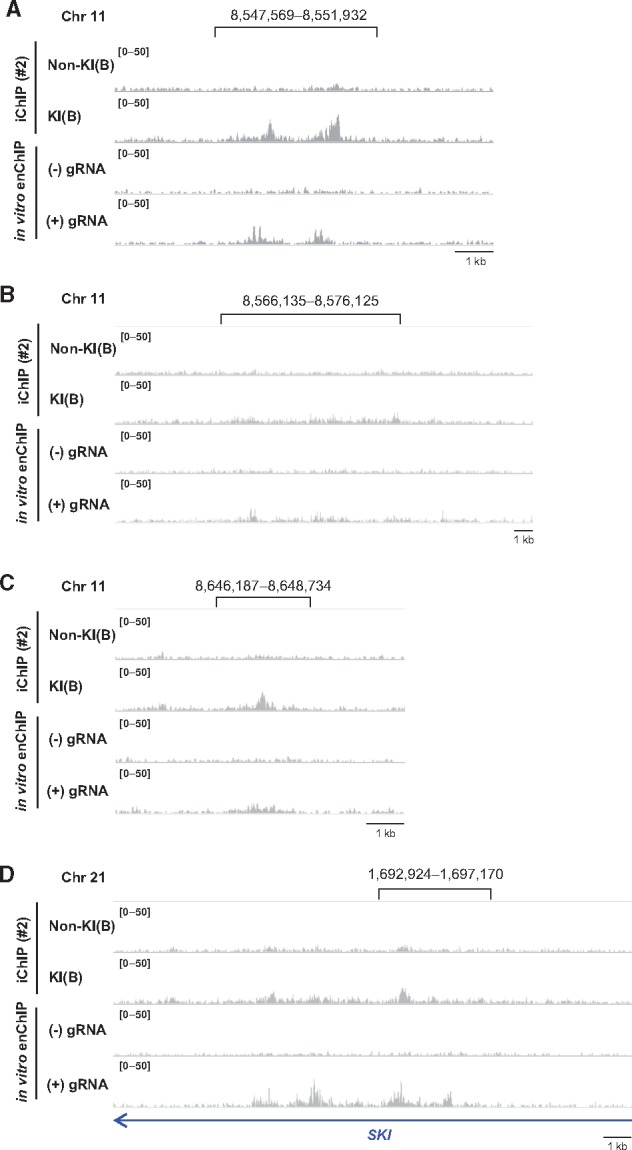
Genomic regions that physically interact with the *Pax5* promoter region. (**A**–**D**) iChIP-Seq data [#2, Non-KI(B) and KI(B)] and *in vitro* enChIP-Seq data (with or without Pax5 gRNA) were displayed in IGV. Representative regions in chromosome 11 (**A**–**C**) and chromosome 21 (**D**) are shown. The vertical viewing range (*y*-axis shown as scale) was set at 0–50 based on the noise peaks. The same loci in the iChIP-Seq data [#1, Non-KI(B) and KI(B)] are shown in Supplementary Figure S3.

We designed a guide RNA (Pax5 gRNA) recognizing a 23 bp target site that is 0.1 kb upstream from the TSS of the *Pax5* exon 1A (Fig. 3B and Supplementary Fig. S2); the recognized DNA sequence exists only in the target site, i.e. nowhere else in the chicken genome. We performed *in vitro* enChIP with Pax5 gRNA to specifically isolate the *Pax5* promoter region from intact DT40. Isolation of the *Pax5* promoter region was confirmed by NGS analysis (*in vitro* enChIP-Seq) (Fig. 3C) and PCR (Supplementary Fig. S2). Next, we examined whether the 33 peaks identified by iChIP-Seq (Step 3 in Fig. 2) were also observed by *in vitro* enChIP-Seq (Fig. 3D). Based on visual confirmation in IGV, a high-performance visualization tool, approximately half of the peaks (14 peaks) were also observed by *in vitro* enChIP-Seq in the presence of Pax5 gRNA but not in the absence of gRNA (Table 1 and Supplementary Table S3); representative results are shown in Figure 4 and Supplementary Figures S3 and S4. CRISPR binds to DNA sequences similar to the target sequence, a phenomenon known as off-target binding.^44–47^ However, potential off-target binding sites were not found in the 14 identified genomic regions (Supplementary Fig. S5). Thus, the genomic regions independently confirmed by *in vitro* enChIP-Seq (Table 1) can be considered as those that physically interact with the *Pax5* promoter region in DT40 cells. These results show that, in addition to iChIP-microarray,^17^ iChIP-Seq would be a useful tool for non-biased identification of physical chromosomal interactions.

**Table 1:** Genomic regions potentially interacting with the *Pax5* promoter in DT40

chr	Start	End	Length	Summit	Tags	= −10*LOG10(pvalue)	fold_enrichment	Genes (fwd)	Genes (rev)	B cell-specific or Constitutive [iChIP-Seq_KI(B) vs. iChIP-Seq_KI(Mφ)]	Names of regions (Figs 6 and 7)
chr11	8547569	8551932	4364	3237	576	604	9.7			B cell-specific	IRC11-1
chr11	8566135	8576125	9991	9522	737	242	9.5			B cell-specific	IRC11-2
chr21	3171858	3177104	5247	3845	542	91	9.0			Constitutive	—
chr21	6343936	6355287	11352	7637	1091	171	8.3	20582:LEPRE1:NM_001001529,|	−1689:CDC42:NM_205048,|	Constitutive	—
chr21	5832758	5834832	2075	1457	215	179	8.2	2230:GUCA2A:NM_001197038,|		Constitutive	—
chr21	1692924	1697170	4247	939	352	105	7.9			B cell-specific	—
chr21	1907673	1913024	5352	4697	618	120	7.8			Constitutive	—
chr21	3253691	3261188	7498	1131	689	214	7.7		−5551:MIR34A:NR_031475,|	Constitutive	—
chr21	3550015	3551370	1356	801	125	159	7.4			B cell-specific	—
chr21	5512600	5515428	2829	1629	362	334	7.3			Constitutive	—
chr11	8646187	8648734	2548	1274	232	240	7.1			B cell-specific	IRC11-3
chr21	744369	746776	2408	1674	231	125	7.1			B cell-specific	—
chr21	6596929	6598628	1700	1172	144	244	7.0	3330:ECE1:NM_204717,|	910:ALPL:NM_205360,|	Constitutive	—
chr21	4371052	4372756	1705	868	142	263	7.0	1794:C21H1orf144:NM_001030880,|		Constitutive	—

The genomic regions detected in iChIP-Seq_KI(B) (#2) are shown. Those corresponding to iChIP-Seq_KI(B) (#1) are shown in Supplementary Table S3.

Intra-chromosomal interactions would be more frequently observed than inter-chromosomal interactions by 3C-based techniques.^48^ Although we detected intra-chromosomal interactions using the criterion ‘>2-fold’, percentages of intra-chromosomal interactions were not necessarily high (Supplementary Fig. S6). One of the causes of this may be that sonication was used for fragmentation of chromatin, whereas milder enzymatic digestion is usually used in 3C-based techniques. In fact, it has been reported that 4C-Seq using enzymatic digestion detected intra-chromosomal interactions much more frequently than 4C-Seq using sonication-based fragmentation.^49^ In addition, it has been reported that sonication weakens the signals detected by 3C using enzymatic digestion.^50^^,^^51^ Neighboring loci in the same chromosome would be spatially proximal each other in a chromosomal compartment. Weak chromosomal interactions such as random (non-specific) collision in the compartment would not be disrupted by gentle restriction enzyme digestion and detected much more frequently as intra-chromosomal interactions by 3C-based technologies using enzymatic digestion. By contrast, such weak interactions and chromosomal compartments could be disrupted by sonication.^50^^,^^52^ Strong chromosomal interactions mediated by specific mediators could be retained even under fragmentation by sonication. Thus, sonication method used for fragmentation of chromatin in our analyses may disrupt such weak intra-chromosomal interactions.

Interestingly, most of the identified genomic regions were localized in chromosome 21 (Table 1 and Supplementary Table S3). Because those regions were spread equally in chromosome 21 (Supplementary Fig. S7), the entire chromosome 21 might interact with the *Pax5* gene (or chromosome Z on which the *Pax5* gene is located) in the nucleus of DT40 cells. On the other hand, some peaks identified by iChIP-Seq were not confirmed by *in vitro* enChIP-Seq. In this regard, insertion of LexA BE might partially change the chromosomal conformation around the insertion site and organize artificial chromosomal interactions, which would not be confirmed in intact DT40 by *in vitro* enChIP. Alternatively, *in vitro* enChIP might fail to confirm some *bona fide* chromosomal interactions identified by iChIP-Seq. Because the insertion site of LexA BE is 0.2 kb upstream from the target site of the Pax5 gRNA (Supplementary Fig. S8), iChIP can capture chromosomal interactions organized in the more upstream region of the *Pax5* promoter, whereas *in vitro* enChIP might fail to confirm such chromosomal interactions.

### 3.4. Identification of genomic regions that physically interact with the *Pax5* promoter region in a B cell-specific manner

**Figure 5.**
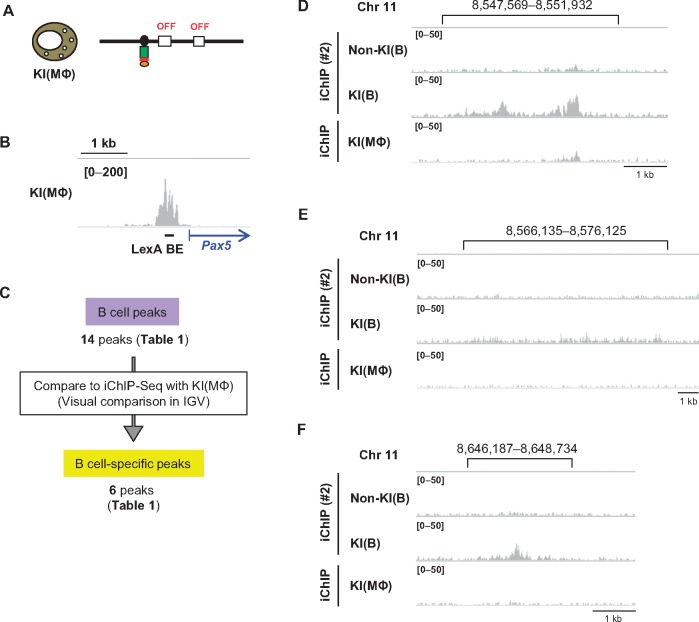
Extraction of genomic regions interacting with the *Pax5* promoter region in a B cell-specific manner. (**A**) In KI(MΦ), which is KI(B) trans-differentiated into a macrophage-like cell, *Pax5* gene is not transcribed. (**B**) Image of NGS peaks around the *Pax5* promoter region. NGS data from iChIP-Seq of KI(MΦ) were visualized in IGV. The vertical viewing range (*y*-axis shown as scale) was set at 0–200 based on the magnitude of the noise peaks. (**C**) Extraction of genomic regions interacting with the *Pax5* promoter region in a B cell-specific manner. Peak positions identified by iChIP-Seq of KI(B) were compared with the results of iChIP-Seq of KI(MΦ) in IGV. (**D**–**F**) Representative genomic regions observed in a B cell-specific manner. The images from iChIP-Seq (#2) in Figure 4A–C are also shown here for comparison with those from iChIP-Seq of KI(MΦ).

The chromosomal interactions identified above might be organized in a B cell-specific manner or occur constitutively in different cell types. Therefore, we next examined whether the 14 interacting genomic regions (Table 1) could be detected in NGS data of iChIP-Seq of KI(MΦ), which is KI(B) trans-differentiated into a macrophage-like cell by expression of chicken C/EBPβ.^15^ KI(MΦ) expresses M-CSFR, a macrophage marker, but neither Pax5 nor AID, another B cell marker (Fig. 5A).^15^ As shown in Figure 5B, the *Pax5* promoter region was isolated from KI(MΦ) by iChIP. Visual comparison in IGV revealed that three peaks in chromosome 11 and three peaks in chromosome 21 (total six peaks) were observed in a B cell-specific manner, whereas the other eight peaks were constitutively observed both in the B cell and the macrophage-like cell lines (Fig. 5C, Table 1); the three B cell specific peaks in chromosome 11 and two constitutive peaks in chromosome 21 are shown as representatives in Figure 5D–F and Supplementary Figure S9, respectively. Thus, by comparing iChIP-Seq data, we were able to identify genomic regions that interact with the *Pax5* promoter region in a B cell-specific manner.

We also attempted *in vitro* enChIP-Seq with the macrophage-like cell line DT40(MΦ), which is DT40 trans-differentiated into a macrophage-like cell by ectopic expression of chicken C/EBPβ (Supplementary Fig. S10A–C). However, *in vitro* enChIP with Pax5 gRNA failed to isolate the *Pax5* promoter region from DT40(MΦ) (Supplementary Fig. S10D), suggesting that the CRISPR RNP was unable to access the gRNA target site. Because *Pax5* transcription is silenced in DT40(MΦ) (Supplementary Fig. S10B), the *Pax5* promoter might be heterochromatinized. Alternatively, effector molecules, such as transcriptional repressors, might occupy the gRNA target site, which would block access by the CRISPR RNP.

### 3.5. Regulation of expression of *Pax5* by a physical interaction between genomic regions

**Figure 6.**
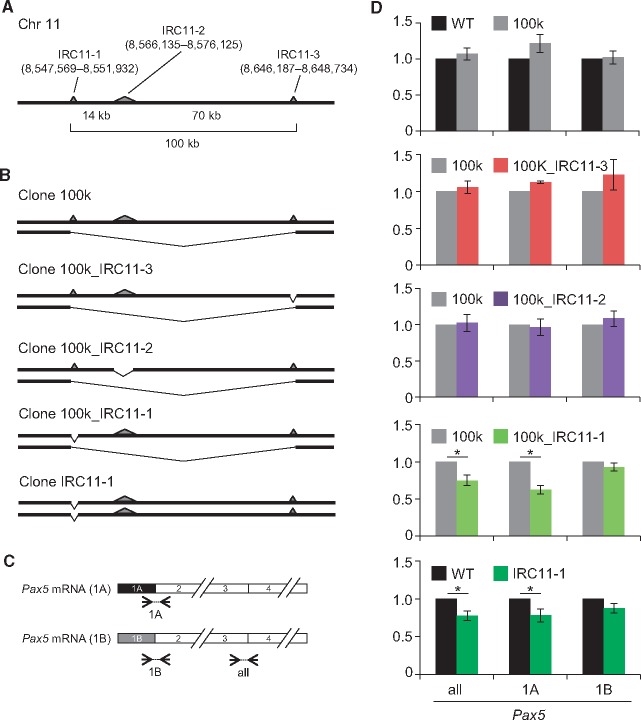
A genomic region interacting with the *Pax5* promoter is involved in transcriptional regulation of the *Pax5* gene. (**A**) Schematic depiction of the loci in chromosome 11 that were identified as interacting with the *Pax5* promoter region in a B cell-specific manner. One allele is shown. The interacting regions are shown as gray triangles. (**B**) CRISPR-mediated knock-out of the interacting regions in DT40. (**C**) Primer sets for evaluation of the amounts of *Pax5* mRNA. (**D**) Expression levels of the *Pax5* gene in the knock-out cells. Expression levels of *Pax5* were normalized to those of *GAPDH*, and the mRNA levels in the control cells were defined as 1 [mean ± s.e.m., *n* = 3 (two upper graphs), *n* = 6 (others)]. WT: DT40. *: *t*-test *P* value < 0.05. all: mRNA transcribed from both the *Pax5* exons 1A and 1B, 1A: mRNA transcribed from the *Pax5* exon 1A, 1B: mRNA transcribed from the *Pax5* exon 1B.

The identified genomic regions (Table 1) might include transcriptional regulatory regions that control *Pax5* transcription through chromosomal interactions. To examine this possibility, we used CRISPR-mediated genome editing to delete genomic regions that B cell-specifically interacted with the *Pax5* promoter region.^11^^,^^12^ We chose the three regions in chromosome 11 for locus deletion because they are within 100 kb of each other, and it was therefore feasible to delete all of them at once (Fig. 6A), and two of those regions are highly ranked in Table 1. We refer to these three regions (Chr11: 8,547,569–8,551,932; Chr11: 8,566,135–8,576,125; and Chr11: 8,646,187–8,648,734) as Interacting Region in Chromosome 11 No. 1 (IRC11-1), IRC11-2, and IRC11-3, respectively (Table 1). We constructed plasmids for expression of sgRNAs targeting each end of those genomic regions (Supplementary Fig. S11A–C) and co-transfected them with a Cas9 expression plasmid to delete the target genomic regions (Supplementary Fig. S11). We were able to delete all three regions (100 kb) in one allele in DT40 (Fig. 6B and Supplementary Figs S11D and S12). In the resultant cells (Clone 100k), the transcript levels of the *Pax5* gene were not changed (Fig. 6C and D). Next, we deleted each interacting genomic region (IRC11-1, IRC11-2, or IRC11-3) in the other allele in Clone 100k (Fig. 6B and Supplementary Figs S11D and S12). Additional deletion of IRC11-2 or IRC11-3 did not have any effects on *Pax5* transcription, whereas deletion of IRC11-1 moderately but significantly decreased transcription of *Pax5* (Fig. 6C and D). In DT40, *Pax5* was transcribed comparably from the exons 1A and 1B.^53^ Deletion of IRC11-1 decreased transcription from the exon 1A, but not 1B (Fig. 6C and D). To further confirm the physiological importance of IRC11-1 for *Pax5* transcription, we deleted only this region from both alleles in DT40 (Fig. 6B and Supplementary Figs S11E and S12). The resultant cells (Clone IRC11-1) also exhibited reduced *Pax5* transcription from the exon 1A (Fig. 6C and D). Thus, the decrease in levels of *Pax5* transcription in two independently established cell lines (Clone 100k_IRC11-1 and Clone IRC11-1) suggested that IRC11-1 is involved in transcriptional regulation of the *Pax5* gene, acting as an enhancer via inter-chromosomal interaction.

**Figure 7.**
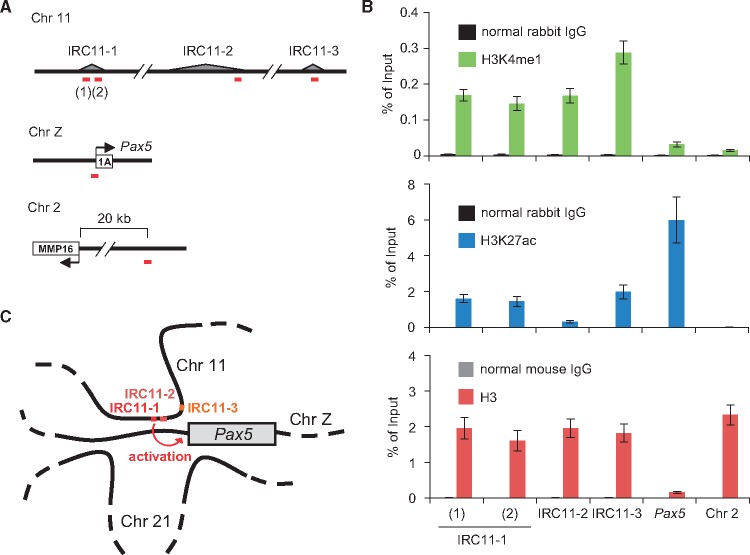
Enrichment of active enhancer marks on the identified genomic regions. (**A**) Positions of primer sets (red lines) used in ChIP assays. (**B**) ChIP assays. DT40 cells were used for ChIP assays with an antibody against H3K4me1 (top), H3K27ac (middle), or H3 (bottom) (means ± s.e.m., *n* = 3). (**C**) A potential regulation mechanism of *Pax5* transcription through B cell-specific inter-chromosomal interaction in chicken B cells.

Active enhancers are marked by enrichment of histone H3 lysine 4 mono-methylation (H3K4me1) and histone H3 lysine 27 acetylation (H3K27ac).^54–57^ We therefore investigated whether IRC11-1 is marked by these histone modifications. Because two peaks were observed in IRC11-1 by iChIP-Seq analyses (Fig. 4A and Supplementary Fig. S3A), we examined these histone modifications at both positions. ChIP assays clearly showed that these histone modifications were enriched at both positions in IRC11-1 but not in an irrelevant genomic region in chromosome 2 (Fig. 7), suggesting that IRC11-1 functions as a distal enhancer for *Pax5* transcription. Enrichment of the active enhancer marks was also observed in IRC11-3, whereas only H3K4me1 was enriched in IRC11-2 (Fig. 7). Therefore, IRC11-3 might be involved in transcriptional regulation of genes other than *Pax5*.

Deletion of the 100 kb region including IRC11-1 in one allele did not have any effects on *Pax5* transcription (Clone 100k in Fig. 6D). Because chromosome Z, which contains *Pax5*, is a single-copy chromosome in DT40,^58^^,^^59^ IRC11-1 in each allele may be sufficient for transcription of the single-copy *Pax5* gene. In addition, deletion of IRC11-1 significantly but only partially down-regulated *Pax5* transcription, suggesting that it plays a limited role in *Pax5* transcription. In IRC11-1, two sub-regions, which are marked by H3K4me1 and H3K27ac, interacted strongly with the *Pax5* promoter (two peak positions in Fig. 4A and Supplementary Fig. S3A), suggesting that each sub-region might work independently or collaboratively to regulate *Pax5* transcription from the exon 1A. On the other hand, we cannot exclude a possibility that IRC11-1 might indirectly control transcription of the *Pax5* gene through regulation of transcription of other genes. In this context, it might be possible that IRC11-1 interacts with promoters of other genes for regulation of their transcription. Deletion of IRC11-1 might affect transcription of those genes, which might result in suppression of *Pax5* transcription through alteration of multiple signaling pathways. Future work should seek to elucidate the mechanistic details underlying transcriptional regulation of *Pax5* by IRC11-1.

## 4. Conclusions and perspectives

In this study, we identified physical chromosomal interactions between the *Pax5* promoter and other genomic regions by locus-specific ChIP in combination with NGS analysis. iChIP-Seq and *in vitro* enChIP-Seq revealed that the *Pax5* promoter binds to multiple genomic regions, in which most regions are localized in chromosome 21 (Figs 1–4). Some of these interactions were organized in a B cell-specific manner (Fig. 5). In addition, we showed that deletion of an interacting genomic region in chromosome 11, which is marked by active enhancer histone modifications, decreased transcriptional levels of the *Pax5* gene (Figs 6 and 7), suggesting its physiological involvement in transcriptional regulation of the *Pax5* gene. To our knowledge, this study is the first report to reveal physical chromosomal interactions focusing on the *Pax5* gene and a regulation mechanism of *Pax5* transcription through B cell-specific inter-chromosomal interaction (Fig. 7C). In this study, we used the chicken B cell line DT40 as a model B cell. It would be an interesting future study to examine whether these chromosomal interactions play any roles in *Pax5* transcription in different species.

Our results also indicate that locus-specific ChIP in combination with NGS analysis is a useful tool for performing non-biased searches for physical chromosomal interactions (one-to-many interactions). Thus, this technology could facilitate elucidation of the molecular mechanisms underlying regulation of genome functions, including transcription.

Several methods have been utilized for detection of genome-wide chromosomal interaction. However, observation by only a single method might not accurately reflect physiological chromosomal interactions. In this regard, potential discrepancies have been reported between the results of FISH and those of 3C or its derivatives.^60^ Therefore, in analysis of chromosomal interactions, it may be necessary to combine several independent methods to eliminate potential contamination of artifactual signals. In this regard, iChIP-Seq (and *in vitro* enChIP-Seq) could be used as one of several methods. In this study, we used *in vitro* enChIP-Seq just to confirm the results of iChIP-Seq. We believe that *in vitro* enChIP is potentially useful for identification of chromosomal interactions. However, to make the claim of utility of this technology stronger, more analyses using other gRNAs (e.g. more than two gRNAs targeting one target locus or other transcribed genes as controls) should be necessary. Nevertheless, considering its convenience, *in vitro* enChIP-Seq may be preferable for future identification of chromosomal interactions.

## Data availability

The accession number of the NGS data is DRA005236 (https://www.ncbi.nlm.nih.gov/sra/?term=DRA005236 (12 May 2017, date last accessed)).

## Supplementary Material

Supplementary Data

Supplementary Data

Supplementary Data

Supplementary Data

Supplementary Data

Supplementary Data

Supplementary Data
