# Supplementary material for: Locus-specific ChIP combined with NGS analysis reveals genomic regulatory regions that physically interact with the Pax5 promoter in a chicken B cell line
Source: DNA Res. 2017 Jun 6;24(5):537–48. doi: 10.1093/dnares/dsx023 (PMC5737561; doi:10.1093/dnares/dsx023)
Supplement: Supplementary Data [file dsx023_supp_2017_fujita_genes_cells_in_press.pdf]

Identification of physical interactions between genomic regions by enChIP-Seq

Toshitsugu Fujita, Miyuki Yuno, Yutaka Suzuki, Sumio Sugano, and Hodaka Fujii*

Toshitsugu Fujita, Miyuki Yuno, Hodaka Fujii

Chromatin Biochemistry Research Group, Combined Program on Microbiology and Immunology, Research Institute for Microbial Diseases, Osaka University, Suita, Osaka, Japan

Yutaka Suzuki, Sumio Sugano

Department of Medical Genome Sciences, Graduate School of Frontier Sciences, The University of Tokyo, Kashiwa, Chiba, Japan

Department of Medical Genome Sciences, Graduate School of Frontier Sciences, The University of Tokyo, Minato-ku, Tokyo, Japan

*Corresponding author

E-mail: hodaka@biken.osaka-u.ac.jp

Chromatin Biochemistry Research Group, Combined Program on Microbiology and Immunology, Research Institute for Microbial Diseases, Osaka University

3-1 Yamadaoka, Suita, Osaka 565-0871, Japan

Short title:

enChIP-Seq to map genomic organization

Abstract

Physical interactions between genomic regions play critical roles in the regulation of genome functions, including gene expression. Here, we demonstrate the feasibility of using engineered DNA-binding molecule-mediated chromatin immunoprecipitation (enChIP) in combination with next-generation sequencing (NGS) (enChIP-Seq) to detect such interactions. In enChIP-Seq, the target genomic region is captured by an engineered DNA-binding complex, such as a clustered regularly interspaced short palindromic repeats (CRISPR) system consisting of a catalytically inactive form of Cas9 and a single guide RNA. Subsequently, the genomic regions that physically interact with the target genomic region in the captured complex are sequenced by NGS. Using enChIP-Seq, we found that the 5'HS5 locus, which is involved in the regulation of *globin* genes expression at the *β-globin* locus, interacts with multiple genomic regions upon erythroid differentiation in the human erythroleukemia cell line K562. Genes near the genomic regions inducibly associated with the 5'HS5 locus were transcriptionally up-regulated in the differentiated state, suggesting the existence of a coordinated transcription mechanism mediated by physical interactions between these loci. Thus, enChIP-Seq might be a potentially useful tool for detecting physical interactions between genomic regions in a non-biased manner, which would facilitate elucidation of the molecular mechanisms underlying regulation of genome functions.

Introduction

Physical interactions between genomic regions play important roles in the regulation of genome functions, including transcription and epigenetic regulation (Williams *et al.* 2010). Several techniques, such as fluorescence *in situ* hybridization (FISH) (Fraser & Bickmore 2007), chromosome conformation capture (3C), and 3C-derived methods (Dekker *et al.* 2002; de Wit & de Laat 2012), have been used to detect such interactions. Although these techniques are widely used, they have certain limitations. The resolution of FISH is low, i.e., apparent co-localization of FISH signals does not necessarily mean that the loci in question physically interact. In addition, FISH cannot be used in a non-biased search for interacting genomic regions. In 3C and related methods, molecular interactions are maintained by crosslinking with formaldehyde prior to digestion with a restriction enzyme(s). The digested DNA is purified after ligation of the DNA ends within the same complex. Interaction between genomic loci is detected by PCR using locus-specific primers or next-generation sequencing (NGS). As with FISH, 3C-based approaches also have intrinsic drawbacks. For example, these methods require enzymatic reactions, including digestion with restriction enzyme(s) and ligation of crosslinked chromatin; the difficulty of achieving complete digestion of crosslinked chromatin can result in detection of artifactual interactions. In addition, in 3C and its derivatives, it is difficult to distinguish the products of intra-molecular and inter-molecular ligation reactions, although several attempts to overcome these problems have been reported (Kalhor *et al.* 2011; Nagano *et al.* 2015). These drawbacks of 3C-based methods make it potentially difficult to ensure that the detected signals truly reflect physical interactions between different genomic regions.

An alternative approach to detecting physical interactions between genomic regions is to purify specific genomic regions engaged in molecular interactions and then analyze the genomic DNA in the purified complexes. To purify specific genomic regions, we recently developed two locus-specific chromatin immunoprecipitation (locus-specific ChIP) technologies, insertional ChIP (iChIP) (Hoshino & Fujii 2009; Fujita & Fujii 2011, 2012a, 2014a; Fujita *et al.* 2015a) (see review (Fujita & Fujii 2016)) and engineered DNA-binding molecule-mediated ChIP (enChIP) (Fujita *et al.* 2013; Fujita & Fujii 2013, 2014b; Fujita *et al.* 2015b; Fujita *et al.* 2016b, 2016a) (see reviews (Fujii & Fujita 2015; Fujita & Fujii 2015)). enChIP consists of the following steps (Fig. 1): (i) A DNA-binding molecule or complex (DB) that recognizes a target DNA sequence in a genomic region of interest is engineered. Zinc-finger proteins (Pabo *et al.* 2001), transcription activator-like (TAL) proteins (Bogdanove & Voytas 2011), and a clustered regularly interspaced short palindromic repeats (CRISPR) system (Qi *et al.* 2013) consisting of a catalytically inactive Cas9 (dCas9) plus a single guide RNA (sgRNA) can be used as the DB. Tag(s) and a nuclear localization signal (NLS)(s) can be fused with the engineered DB; (ii) The fusion protein(s) are expressed in the cells of interest; and (iii) If necessary, the DB-expressing cells are stimulated and crosslinked with formaldehyde or other crosslinkers. The cells are lysed, and chromatin is fragmented by sonication or digested with nucleases. (iv) Chromatin complexes containing the engineered DB are affinity-purified by immunoprecipitation or other methods. (v) After reverse crosslinking (if necessary), DNA, RNA, proteins, or other molecules are purified and identified by various methods including NGS and mass spectrometry (MS).

As a model locus in this study, we focused on 5'HS, an extensively analyzed locus that has shown to regulate expression of *globin* genes at the *β-globin* locus during development (Fig. 2a) (Ghirlando *et al.* 2012; Holwerda & de Laat 2013). The 5'HS2-4 regions in the 5'HS locus

behave as enhancers for expression of the *globin* genes (Caterina *et al.* 1991; Peterson *et al.* 1996; Navas *et al.* 2001). By contrast, 5'HS5 functions as an insulator to prevent invasion of heterochromatin into the *β-globin* locus (Farrell *et al.* 2002). In addition, the 5'HS5 locus interacts with the 3'HS1 locus in the 3' region of the *β-globin* locus (Dostie *et al.* 2006; Chien *et al.* 2011). Moreover, CTCF, a major component of the insulator complex, plays a critical role in insulation and formation of a chromatin loop (Farrell *et al.* 2002; Chien *et al.* 2011). However, the molecular mechanisms underlying the functions of 5'HS5 remain incompletely understood.

Here, we combined enChIP with NGS (enChIP-Seq) to detect genomic regions that physically interact with the 5'HS5 locus. Using enChIP-Seq, we showed that the 5'HS5 locus physically interacts with multiple genomic regions upon erythroid differentiation in the human erythroleukemia cell line K562. Thus, enChIP-Seq represents a potentially useful tool for analysis of genome functions.

Results and Discussion

Isolation of the 5'HS5 locus by enChIP using the CRISPR system

To purify the 5'HS5 locus by enChIP using the CRISPR system (Figs. 1 and 2a) (Fujita & Fujii 2013, 2014b), we generated human erythroleukemia K562-derived cells expressing 3xFLAG-dCas9 (Fujita & Fujii 2014b) and sgRNA targeting the 5'HS5 locus (Fig. 2a). We designed two sgRNAs (#6 and #17) to target different sites in the 5'HS5 locus separated by 52 bp (Fig. 2b). These target sites were taken from a region 0.3 - 0.4 kb downstream of the CTCF-binding site and 5'HS5 core site to prevent CRISPR complex binding from influencing the intrinsic functions of the 5'HS5 locus. Like the parental K562 cells, the derivative cells were white, suggesting that they did not spontaneously express *globin* genes as a result of introduction of the CRISPR complex or its binding to the target locus. Crosslinked chromatin was fragmented by sonication and subjected to affinity purification with anti-FLAG antibody (Ab) (Fig. 1b). Subsequently, crosslinking was reversed and DNA was purified from the isolated chromatin. As shown in Fig. 2c, 0.1-0.4% of the input 5'HS5 locus was isolated, whereas the irrelevant *SOX2* locus was not enriched, suggesting that enChIP using either of the two sgRNAs (#6 and #17) could isolate the 5'HS5 locus.

Detection of genomic regions that physically interact with the 5'HS5 locus

To identify genomic regions associated with the 5'HS5 locus on a genome-wide scale in erythroid cells under undifferentiated or differentiated conditions, K562-derived cells were mock-treated or treated with sodium butyrate (NaB) for 4 days before being crosslinked with formaldehyde and subjected to enChIP. The K562-derived cells changed from white to pink upon

NaB treatment, suggesting that they had begun to express the *globin* genes, as wild-type K562 cells do in response to NaB. After isolating the 5'HS5 locus by enChIP, we subjected the purified DNA to NGS analysis. As expected, reads corresponding to the 5'HS5 locus were clearly detected in cells expressing either sgRNA #6 or #17, but not in cells expressing neither sgRNA (Fig. 2d and Table 1). By contrast, no peak was detected at the irrelevant *Sox2* locus (Fig. 2e).

To identify genomic regions physically interacting with 5'HS5 upon erythroid differentiation, we analyzed the NGS peaks observed in the differentiated state. The CRISPR complex can interact with multiple genomic sites containing sequences similar to the sgRNA sequence (Cencic *et al.* 2014; Kuscu *et al.* 2014; Wu *et al.* 2014; O'Green *et al.* 2015). In addition, in the absence of sgRNA, it is possible that dCas9 could bind non-specifically to some genomic sites *in vivo*. Therefore, the peaks identified for sgRNA #6 and #17 may include such off-target sites. To remove those off-target sites, we first eliminated peaks derived from non-specific binding of dCas9 in the absence of sgRNA from those identified for each sgRNA (Steps 1 and 2 in Fig. 3). To identify peaks with confidence, we next established two criteria for choosing peaks based on NGS information from the target 5'HS5 locus: (i) tag number >5% of that of the target 5'HS5 locus, and (ii) fold enrichment relative to input genomic DNA >10. As shown in Fig. 3 (Step 2), 19 and 228 peaks for sgRNA #6 and #17, respectively, fulfilled these criteria. Next, to eliminate sgRNA-dependent off-target sites, we compared the peaks for sgRNA #6 and #17 and selected peaks detected in common by both sgRNA #6 and #17. These peaks were considered to represent regions engaged in bona fide physical interactions with the 5'HS5 locus (Step 3 in Fig. 3). The six identified peaks could be classified into two categories: (i) peaks that were larger in the differentiated state, and (ii) peaks constitutively observed in both the undifferentiated and differentiated states. The first category should contain genomic regions that inducibly associate with the 5'HS5 locus upon erythroid differentiation, whereas the second category should contain

genomic regions constitutively associated with the 5'HS5 locus. To extract the peaks that grew larger specifically in the differentiated state, we selected peaks observed constitutively or in the undifferentiated state (Step 4 in Fig. 3) and compared them with the six peaks extracted in Step 3 (Step 5 in Fig. 3). As shown in Fig. 3 (Step 5) and Table 2, the 5'HS5 site was the unique peak constitutively observed in the undifferentiated and differentiated states, whereas the five other peaks were larger specifically in the differentiated state. These included one intra-chromosomal interaction and four inter-chromosomal interactions (Table 2); the two peaks on chromosome 1 corresponding to inter-chromosomal interactions were adjacent to each other in the primary sequence. As for the associated genomic regions, their tag numbers were much lower than those of the target genomic region (Tables 1 and 2). This is reasonable considering that associated genomic regions are likely to have been indirectly purified by enChIP. In other words, not all complexes of the target genomic region would be expected to contain associated genomic regions. The binding stoichiometry of the associated genomic regions could be affected by multiple factors, such as the cell cycle phase the cells were in at the time of harvesting. Next, we attempted to extract genomic regions that interacted with the 5'HS5 locus specifically in the undifferentiated state. However, bioinformatics analysis based on the aforementioned criteria identified no regions in this category (Supplementary Fig. S1).

We visualized some of the identified peaks in the UCSC Genome Browser (Fig. 4). The peaks were clearly visible for both sgRNA #6 and #17. The adjacent NaB-specific peaks in chromosome 1 were both located in the first intron of the *ZNF670* and *ZNF670-ZNF695* genes. The other NaB-specific peaks were located in the vicinity of the *MIR422A* gene in chromosome 15 and between the *TMEM151A* and *YIF1A* genes in chromosome 11.

To confirm the interactions identified by enChIP-Seq, we used the ligation-mediated approach used in 3C-based assays (Supplementary Fig. S2). In this approach, cells are subjected to crosslinking, and then chromatin is randomly fragmented by sonication. After proximity ligation of genomic DNA, the junction between the target locus and a potential interacting site is amplified by PCR. Subsequently, a part of the amplified region in the potential interacting locus is detected by a second PCR. Amplification of a region in the second PCR suggests that the potential interacting locus is physically proximal to the target locus. When we used this assay to examine the interaction between the 5'HS5 locus and the *ZNF670/ZNF670-ZNF695* locus, the second PCR amplified the *ZNF670/ZNF670-ZNF695* locus in an NaB-specific manner only when the proximal ligation step was performed (Fig. 5a and Supplementary Fig. S3a). This observation is consistent with the enChIP-Seq result (Fig. 4), showing that the 5'HS5 locus interacts with the *ZNF670/ZNF670-ZNF695* locus in the differentiated state in K562 cells. In addition, we observed similar results for *MIR422A* and *YIF1A* loci (Fig. 5b and c and Supplementary Fig. S3b and c). Thus, we were able to confirm the chromosomal interactions identified by enChIP-Seq by another independent method, suggesting that it is feasible to use enChIP-Seq to perform non-biased identification of physical interactions between genomic regions. It is an interesting future issue how interactions between 5'HS5 and these regions are mediated.

Transcription of genes near the 5'HS5-interacting genomic regions identified by enChIP-Seq could be directly or indirectly regulated by the induced association with the 5'HS locus. Such regulation could involve the 5'HS2-4 regions, which function as enhancers (Caterina *et al.* 1991; Peterson *et al.* 1996; Navas *et al.* 2001). Therefore, we investigated whether mRNA levels of the genes in the vicinity (±10 kb) of the 5'HS5-interacting genomic regions changed after NaB treatment. As shown in Fig. 6, mRNA levels of the *ZNF670*, *MIR422A*, and *CNIH2* genes were

clearly up-regulated in the NaB-induced differentiated state, suggesting that the identified chromosomal interactions are involved in transcriptional regulation of these genes. At this time, it is not clear whether these gene products play any roles in erythroid development. Future studies should attempt to elucidate how the 5’HS locus regulates transcription of these genes. It is possible that the enhancer function of the 5’HS locus directly activates transcription of these genes via interactions with their promoters under differentiated conditions. Alternatively, these loci could be incorporated into the “transcription factory” (Deng *et al.* 2013) upon erythroid differentiation, independent of the enhancer function of the 5’HS locus. In this regard, it is interesting that expression of the *YIF1A* gene, which is closer to the region interacting with 5'HS5, is not induced by NaB treatment, whereas expression of the *CNIH2* gene, which is more distal to the interacting region, is markedly induced. There would be a couple of possibilities to explain differential effects of interaction of 5'HS5 to this genomic region on expression of genes in the vicinity. First, interactions of 5'HS5 might not be the sole determinant of gene expression of the nearby genes. It might be possible that transcription factors essential for *YIF1A* might not be expressed in this cell line. Alternatively, *YIF1A* transcription might be actively repressed by repressor factors. Mechanisms of differential regulation of gene expression of this locus would be an interesting future issue.

Interactions between the 5’HS5 locus and globin genes

Studies using 3C and 3C-derived methods suggested that the 5’HS5 locus and *globin* genes (*HBG1* and *HBG2* genes) at the *β-globin* locus interact (Dostie *et al.* 2006; Chien *et al.* 2011). Therefore, we sought to detect a physical interaction between these loci by enChIP-Seq. As shown in Fig. 3 and Table 2, bioinformatics analysis based on the criteria described above (fold enrichment: >10, tag number: >5% of that of the target positions) did not extract genomic regions

around the *HBG1* and *HBG2* genes. In fact, the peak images did not indicate any physical interactions between 5'HS5 and those genes (Supplementary Fig. S4). However, because (1) DNA sequences of *HBG1* and *HBG2* genes show high identity (98%, Supplementary Fig. S5) and (2) multiple mapped reads (NGS reads mapped to multiple loci) cannot be mapped to a reference genome in a standard analytical setting, our NGS data analysis may lack reference information on *HBG1* and *HBG2* genes. Therefore, we manually counted and analyzed the NGS reads corresponding to the DNA sequences of *HBG1* and *HBG2* genes. We counted NGS reads around their transcription start sites (TSSs) (± 0.5 kb), because (1) in general, transcriptional regulatory elements interact with the promoter regions of their target genes to regulate transcription and (2) high DNA sequence identity is detected up to ~ 0.5 kb upstream from the TSSs (Supplementary Fig. S5). As shown in Fig. 7 and Supplementary Table S3, the corresponding NGS reads were enriched in enChIP using sgRNA#6 (2.3-fold enrichment with respect to input reads) and sgRNA#17 (4.0-fold enrichment) in an NaB-specific manner. Such enrichment was not observed in enChIP without sgRNA. We also manually counted NGS reads corresponding to DNA sequences of two irrelevant genes, *interferon γ* (*IFNG*) and *actin α* (*ACTA1*). As shown in Supplementary Fig. S6 and Supplementary Table S4, there was no specific increase in NGS reads around the TSS (± 0.5 kb) of these genes in the enChIP-Seq (NaB) samples. Thus, the interaction between the 5'HS5 locus and those *globin* genes in the differentiated state, could be detected by enChIP-Seq, although we cannot distinguish which genes (i.e., *HBG1* and *HBG2*) mainly interact with the 5'HS5 locus at this stage.

Although the results of 3C showed that the 5'HS5 locus interacts with *HBG1* and *HBG2* even in cells in the undifferentiated state (Dostie *et al.* 2006; Chien *et al.* 2011), these interactions were not clearly observed in enChIP-Seq analysis (Fig. 7). One possibility is that 3C-derived methods

are much more sensitive than enChIP-Seq. Specifically, because 3C-derived methods use PCR amplification to detect ligated regions consisting of different genomic regions, they may be capable of detecting transient or much weaker interactions. In this context, the DNA-looping between 5’HS5 and the *HBG1* / *HBG2* region might be organized more stably after NaB treatment for induction for *HBG1* / *HBG2* transcription, resulting in the detection of NGS reads by enChIP-Seq. Alternatively, the fragmentation of chromatin DNA by the sonication step in enChIP-Seq may be too harsh to retain weak chromosomal interactions. By contrast, 3C-derived methods employ restriction digestion, which is much milder than sonication, to fragment chromatin DNA. In this respect, it was reported that DNA fragmentation by sonication decreases 3C signals (Gavrilov *et al.* 2013). In fact, when sonication was employed for DNA fragmentation, 3C signals between the HS4/5 and *β-globin* loci in mouse liver cells decreased in proportion to sonication time (Gavrilov *et al.* 2013). In addition, INGRID (in-gel replication of interacting DNA segments), another method using sonication to fragment DNA and in-gel PCR to detect chromosomal interactions, could not detect the interaction between HS5 and *β-globin* loci in mouse liver cells (Gavrilov *et al.* 2014).

Dealing with potential aberrant effects of CRISPR complex binding to the target locus

Binding of the CRISPR complex to target sites might block recruitment of endogenous binding proteins, such as transcription factors, to the sites and physically influence chromosome conformation. To avoid detecting such artificial phenomena, we designed two sgRNAs (#6, #17) at different positions in the locus, both of which did not overlap with the CTCF-binding site or the 5’HS5 core region (Fig. 2). If dCas9 with each sgRNA physically disrupts chromosome conformation around the 5’HS5 locus, we would be unable to extract common peak positions through the filtering steps in Fig. 3. However, we were able to extract several peak positions

(Step 5 in Fig. 3 and Table 2). In addition, we succeeded in detecting a chromosomal interaction between the 5'HS5 locus and *HBG1* and *HBG2* genes (Fig. 7). Thus, it seems unlikely that locus targeting with sgRNA#6 and #17 significantly affects physical chromosome interactions around the locus. Alternatively, although locus targeting might partially induce unexpected physical side-effects on chromosome conformation, enChIP-Seq detected chromosomal interactions that were not disrupted. In this regard, enChIP-Seq failed to detect the DNA looping between two CTCF binding sites of 5'HS5 and 3'HS1 (Supplementary Fig. S4). Therefore, the CRISPR complex may negatively affect chromatin conformation and the binding of proteins to DNA. The failure to detect DNA looping between 5'HS5 and 3'HS1 could be due to disruption of loop formation by the CRISPR complex. Alternatively, enChIP might not isolate sufficient amounts of the CTCF-binding site, which is 0.5 kb upstream from sgRNA target sites. It is not surprising that the yields of enChIP decreased as the distance from the sgRNA target site increased. In this context, the loci identified by enChIP-Seq (e.g., *ZNF670*, *HBG1*/*HBG2*) might bind to genomic regions much closer to the sgRNA target sites and allow better detection of DNA-looping.

Managing potential contamination of off-target sites

In this study, we identified physical interactions between genomic regions based on signals detected by enChIP-Seq. dCas9 can bind to multiple sites containing sequences similar to the sgRNA sequence (Kuscu *et al.* 2014; Wu *et al.* 2014). To eliminate potential contamination of our findings by off-target sites, we propose several strategies:

(1) Carefully examine the sequences of the detected peaks and remove those containing sequences similar to the target sequence.

(2) Use different conditions or cell types. Signals specifically detected in one condition or cell type should reflect true physical interactions between genomic regions.

(3) Use multiple different sgRNAs. Because different sgRNA are unlikely to engage in off-target binding at the same genomic regions, signals observed in common using different sgRNAs should reflect true physical interactions between genomic regions. This approach can also help exclude the possibility that CRISPR complex binding to a target site influences physically the intrinsic chromosome conformations around the site. In addition, cells expressing dCas9 without sgRNA should be used as a negative control to eliminate off-target sites associated with dCas9 in the absence of sgRNA. In this respect, cells expressing dCas9 and with sgRNA targeting irrelevant loci could also be used as the negative controls.

(4) Use a sequential purification scheme. Cas9 orthologs derived from different bacterial species recognize distinct proto-spacer adjacent motif (PAM) sequences and can be used for genome editing and gene regulation (Esvelt *et al.* 2013). Tagging of a given locus with dCas9s derived from different lineages and bearing distinct tags would make it feasible to sequentially purify the locus, minimizing contamination with off-target sites. Using these techniques, we believe that we can effectively manage potential contamination of dCas9 off-target sites in enChIP analyses.

In this study, we used enChIP-Seq analysis to detect physical interactions between genomic regions. In K562-derived cells, the 5'HS5 locus physically interacted with multiple regions in the genome (Fig. 4, Table 2). These interactions were induced by erythroid differentiation in response to NaB treatment (Fig. 4, Table 2). Transcription of genes around the interacting genomic region was up-regulated in the differentiated state (Fig. 6), suggesting a direct or indirect involvement of 5'HS enhancer activity in transcription of genes proximal to the interacting sites. In addition, we succeeded in detecting a chromosomal interaction between the 5'HS5 locus and *globin* genes at the *β-globin* locus using enChIP-Seq (Fig. 7). Our results suggest that enChIP-Seq represents a potentially useful tool for performing non-biased searches for physical

interactions between genomic regions, which would facilitate elucidation of the molecular mechanisms underlying regulation of genome functions. Recently, potential discrepancies were reported between the results of 3C or its derivatives and those of FISH (Williamson *et al.* 2014). This highlights the importance of confirming chromosomal interactions by independent methods. enChIP-Seq could be one such method.

Experimental procedures

Design of sgRNAs

All the potential target sites containing the PAM sequence (37 sequences) were obtained from ca. 0.4 kb downstream of the 5’HS5 core site and potential off-target sites were searched for using the GGGenome webtool (http://gggenome.dbcls.jp). Two sequences (#6 and #17) showing the highest specificity were selected to generate sgRNAs.

Plasmids

3xFLAG-dCas9/pMXs-puro (Addgene #51240) was described previously (Fujita & Fujii 2014b). To construct vectors for expression of sgRNAs, two oligos for each sgRNA were annealed and extended using Phusion polymerase (New England Biolabs) to make 100 bp double-stranded DNA fragments, as described previously (Fujita & Fujii 2013). The nucleotide sequences were as follows: hHS5 #6, 5'-TTTCTTGGCTTTATATATCTTGTGGAAAGGACGAAACACCGGATTCATAGCAGACAGCTA-3' and 5'-GACTAGCCTTATTTTAACTTGCTATTTCTAGCTCTAAAACTAGCTGTCTGCTATGAATCC-3'; hHS5 #17, 5'-TTTCTTGGCTTTATATATCTTGTGGAAAGGACGAAACACCGGGAAGATAGGGTAAGAGAC-3' and 5'-GACTAGCCTTATTTTAACTTGCTATTTCTAGCTCTAAAACGTCTCTTACCCTATCTTCCC-3'. Fragments were purified following agarose gel electrophoresis and subjected to Gibson assembly (New England Biolabs) with the linearized sgRNA cloning vector (Addgene #41824), a gift from George Church (Mali *et al.* 2013), to yield sgRNA-hHS5 #6 and sgRNA-hHS5 #17.

The gBlocks were excised with *Xho* I and *Hind* III and cloned into the *Xho* I/*Hind* III-cleaved pSIR vector to generate self-inactivating retroviral vectors for sgRNAs, as described previously (Fujita & Fujii 2014b).

Cell culture

K562-derived cells (Fujita & Fujii 2014b) were maintained in RPMI (Wako) supplemented with 10% fetal calf serum (FCS).

Establishment of cells stably expressing 3xFLAG-dCas9 and sgRNA

The establishment of K562-derived cells expressing 3xFLAG-dCas9 was described previously (Fujita & Fujii 2014b). To establish cells expressing both 3xFLAG-dCas9 and sgRNAs targeting the 5'HS5 locus, 2 µg sgRNA-hHS5 #6/pSIR or sgRNA-hHS5 #17/pSIR was transfected along with 2 µg pPAM3 into 1×10^6 293T cells (Miller & Buttimore 1986). Two days after transfection, K562-derived cells expressing 3xFLAG-dCas9 were infected with the supernatant (5 ml) of 293T cells containing the virus particles. K562-derived cells expressing both 3xFLAG-dCas9 and sgRNA-hHS5 #6 or sgRNA-hHS5 #17 were selected in RPMI medium containing 10% FCS, puromycin (0.5 µg/ml), and G418 (0.8 mg/ml).

Induction of differentiation of K562-derived cells

To induce erythroid differentiation of the K562-derived cells, cells were incubated in the presence of 1 mM sodium butyrate (NaB) for 4 days.

enChIP-real-time PCR

enChIP-real-time PCR was performed as previously described (Fujita & Fujii 2013), except that ChIP DNA Clean & Concentrator (Zymo Research) was used for the purification of DNA. Primers used in the analysis are shown in Supplementary Table S5.

enChIP-Seq and bioinformatics analysis

Undifferentiated or differentiated K562-derived cells (2×10^7 each) expressing 3xFLAG-dCas9 and sgRNAs were subjected to the enChIP procedure as described previously (Fujita & Fujii 2013), except that ChIP DNA Clean & Concentrator was used for the purification of DNA. NGS and data analysis were performed at the University of Tokyo, as described previously (Yamashita *et al.* 2011; Seki *et al.* 2014). Briefly, after fragmentation of chromatin DNA (average fragment length, 2 kbp), the 5'HS5 region was isolated by enChIP. After DNA purification, the enChIP-Seq libraries were purified using TruSeq ChIP Sample Prep Kit (Illumina), which resulted in the selective concentration of DNA fragments with an average size of ~0.4 kbp. The libraries were subjected to DNA sequencing using the HiSeq platform according to the manufacturer's protocol. enChIP-Seq data were mapped to the human reference genome hg19 using ELAND (Illumina). Each enChIP-Seq experiment was performed once. Narrow peaks of each enChIP-Seq dataset were detected using MACS2 using the default parameters. Additional information on enChIP-Seq analysis is provided in Supplementary Table S6. Further data analysis for Step 2 in Fig. 3 was performed at Hokkaido System Science Co., Ltd. Images of NGS peaks were generated using the UCSC Genome Browser (https://genome.ucsc.edu/index.html). The accession number of the enChIP-Seq data is DRA004361 (http://www.ncbi.nlm.nih.gov/sra/?term=DRA004361).

Detection of DNA-DNA interactions to confirm interactions between genomic regions

K562 cells (1 × 10^7) were fixed with 1% formaldehyde at 37°C for 5 min. The chromatin fraction was extracted and fragmented by sonication (average fragment length, 2 kbp), as described previously (Fujita *et al.* 2007), except that TE buffer (800 µl; 10 mM Tris pH 8.0, 1 mM EDTA) and a UD-201 ultrasonic disruptor (TOMY SEIKO) were used. Sonicated chromatin (34 µl) was treated with the End-It DNA End-Repair kit (Epicentre) in a 50 µl reaction mixture at room temperature for 45 min. After heating at 70°C for 10 min, the reaction mixture (23.5 µl) was incubated in the presence or absence of T4 DNA ligase (Roche) at room temperature for 2 h. After reverse crosslinking at 65°C and RNase A and Proteinase K treatment, DNA was purified using ChIP DNA Clean & Concentrator. The purified DNA was used as a template for the first PCR with KOD FX (Toyobo) and a primer set including a primer containing the *I-Sce I* site biotinylated at its 5' end (Supplementary Table S5). PCR conditions were as follows: denaturing at 94°C for 2 min; 40 cycles of 98°C for 10 sec, 60°C for 30 sec, and 68°C for 6 min. The reaction mixture (15 µl) was mixed with 15 µl of Dynabeads M-280 Streptavidin (Thermo Fisher Scientific) and 500 µl of RIPA buffer (50 mM Tris [pH 7.5], 150 mM NaCl, 1 mM EDTA, 0.5% sodium deoxycholate, 0.1% SDS, 1% IGEPAL-CA630) at 4°C for 1 h. After three washes with RIPA buffer and one wash with 1× NEBuffer 2 (New England Biolabs), the Dynabeads were treated with *I-Sce I* at 37°C for 2 h. The supernatant was collected, incubated at 65°C for 20 min, and used for the second PCR with AmpliTaq Gold 360 Master Mix (Applied Biosystems). PCR conditions were as follows: denaturing at 95°C for 10 min; 27 cycles of 95°C for 30 sec, 60°C for 30 sec, and 72°C for 1 min. Primers used in the analysis are shown in Supplementary Table S5. All the amplicons were sequenced to confirm that they were the expected PCR products.

RNA extraction and quantitative RT-PCR

Total RNA was extracted from mock- or NaB-treated K562 cells and used for quantitative RT-PCR, as previously described (Fujita & Fujii 2012b). Primers used in the analysis are shown in Supplementary Table S5.

Acknowledgements

We thank G.M. Church for providing a plasmid (Addgene plasmid #41824), F. Kitaura for technical assistance, and T. Kikuchi, H. Horiuchi, and M. Tosaka for NGS analysis.

Funding

This work was supported by the Asahi Glass Foundation (H.F.), the Uehara Memorial Foundation (H.F.), the Kurata Memorial Hitachi Science and Technology Foundation (T.F. and H.F.), Grant-in-Aid for Young Scientists (B) (#25830131) (T.F.), Grant-in-Aid for Scientific Research (C) (#15K06895) (T.F.), Grant-in-Aid for Scientific Research (B) (#15H04329) (T.F., H.F.), Grant-in-Aid for Scientific Research on Innovative Areas "Cell Fate" (#23118516) (T.F.), "Transcription Cycle" (#25118512 and #15H01354) (H.F.), "Genome Science" (#221S0002) (H.F.), and a Grant-in-Aid for Exploratory Research (#26650059) (H.F.) from the Ministry of Education, Culture, Sports, Science and Technology of Japan.

Abbreviations

enChIP, engineered DNA-binding molecule-mediated chromatin immunoprecipitation; NGS, next generation sequencing; enChIP-Seq, enChIP combined with NGS; CRISPR, clustered regularly interspaced short palindromic repeats; dCas9, a catalytically inactive form of Cas9; sgRNA, single guide RNA; FISH, fluorescence *in situ* hybridization; 3C, chromosome conformation capture; iChIP, insertional ChIP; DB, DNA-binding molecule or complex; TAL, transcription activator-like; NLS, nuclear localization signal; MS, mass spectrometry; NaB, sodium butyrate.

Conflicts of interest

T.F. and H.F. have filed a patent application for enChIP (Patent name: "Method for isolating specific genomic regions using DNA-binding molecules recognizing endogenous DNA sequences"; Patent number: WO2014/125668). T.F. and H.F. are founders of Epigeneron, LLC.

References

Bogdanove, A.J. & Voytas, D.F. (2011) TAL effectors: customizable proteins for DNA targeting. *Science* **333**, 1843-1846.

Caterina, J.J., Ryan, T.M., Pawlik, K.M., Palmiter, R.D., Brinster, R.L., Behringer, R.R. & Townes, T.M. (1991) Human beta-globin locus control region: analysis of the 5' DNase I hypersensitive site HS 2 in transgenic mice. *Proc. Natl. Acad. Sci. U. S. A.* **88**, 1626-1630.

Cencic, R., Miura, H., Malina, A., Robert, F., Ethier, S., Schmeing, T.M., Dosite, J. & Pelletier, J. (2014) Protospacer adjacent motif (PAM)-distal sequences engage CRISPR Cas9 DNA target cleavage. *PLoS One* **9**, e109213.

Chien, R., Zeng, W., Kawauchi, S., Bender, M.A., Santos, R., Gregson, H.C., Schmiesing, J.A., Newkirk, D.A., Kong, X., Ball, A.R.J., Calof, A.L., Lander, A.D., Groudine, M.T. & Yokomori, K. (2011) Cohesin mediates chromatin interactions that regulate mammalian β-globin expression. *J. Biol. Chem.* **286**, 17870-17878.

de Wit, E. & de Laat, W. (2012) A decade of 3C technologies: insights into nuclear organization. *Genes Dev.* **26**, 11-24.

Dekker, J., Rippe, K., Dekker, M. & Kleckner, N. (2002) Capturing chromosome conformation. *Science* **295**, 1306-1311.

Deng, B., Melnik, S. & Cook, P.R. (2013) Transcription factories, chromatin loops, and the dysregulation of gene expression in malignancy. *Semin. Cancer Biol.* **23**, 65-71.

Dostie, J., Richmond, T.A., Arnaout, R.A., Selzer, R.R., Lee, W.L., Honan, T.A., Rubio, E.D., Krumm, A., Lamb, J., Nusbaum, C., Green, R.D. & Dekker, J. (2006) Chromosome Conformation Capture Carbon Copy (5C): a massively parallel solution for mapping interactions between genomic elements. *Genome Res* **16**, 1299-1309.

Esvelt, K.M., Mali, P., Braff, J.L., Moosburner, M., Yaung, S.J. & Church, G.M. (2013) Orthogonal Cas9 proteins for RNA-guided gene regulation and editing. *Nat. Methods* **10**, 1116-1121.

Farrell, C.M., West, A.G. & Felsenfeld, G. (2002) Conserved CTCF insulator elements flank the mouse and human beta-globin loci. *Mol. Cell. Biol* **22**, 3820-3831.

Fraser, P. & Bickmore, W. (2007) Nuclear organization of the genome and the potential for gene regulation. *Nature* **447**, 413-417.

Fujii, H. & Fujita, T. (2015) Isolation of specific genomic regions and identification of their associated molecules by engineered DNA-binding molecule-mediated chromatin immunoprecipitation (enChIP) using the CRISPR system and TAL proteins. *Int. J. Mol. Sci.* **16**, 21802-21812.

Fujita, T., Asano, Y., Ohtsuka, J., Takada, Y., Saito, K., Ohki, R. & Fujii, H. (2013) Identification of telomere-associated molecules by engineered DNA-binding molecule-mediated chromatin immunoprecipitation (enChIP). *Sci. Rep.* **3**, 3171.

Fujita, T. & Fujii, H. (2011) Direct idenification of insulator components by insertional chromatin immunoprecipitation. *PLoS One* **6**, e26109.

Fujita, T. & Fujii, H. (2012a) Efficient isolation of specific genomic regions by insertional chromatin immunoprecipitation (iChIP) with a second-generation tagged LexA DNA-binding domain. *Adv. Biosci. Biotechnol.* **3**, 626-629.

Fujita, T. & Fujii, H. (2012b) Transcription start sites and usage of the first exon of mouse Foxp3 gene. *Mol. Biol. Rep.* **39**, 9613-9619.

Fujita, T. & Fujii, H. (2013) Efficient isolation of specific genomic regions and identification of associated proteins by engineered DNA-binding molecule-mediated chromatin immunoprecipitation (enChIP) using CRISPR. *Biochem. Biophys. Res. Commun.* **439**, 132-136.

Fujita, T. & Fujii, H. (2014a) Efficient isolation of specific genomic regions retaining molecular interactions by the iChIP system using recombinant exogenous DNA-binding proteins. *BMC Mol. Biol.* **15**, 26.

Fujita, T. & Fujii, H. (2014b) Identification of proteins associated with an IFNγ-responsive promoter by a retroviral expression system for enChIP using CRISPR. *PLoS One* **9**, e103084.

Fujita, T. & Fujii, H. (2015) Applications of engineered DNA-binding molecules such as TAL proteins and the CRISPR/Cas system in biology research. *Int. J. Mol. Sci.* **16**, 23143-23164.

Fujita, T. & Fujii, H. (2016) Biochemical analysis of genome functions using locus-specific chromatin immunoprecipitation technologies. *Gene Regul Syst Bio* **Suppl. 1**, 1-9.

Fujita, T., Kitaura, F. & Fujii, H. (2015a) A critical role of the Thy28-MYH9 axis in B cell-specific expression of the *Pax5* gene in chicken B cells. *PLoS One* **10**, e0116579.

Fujita, T., Ryser, S., Tortola, S., Piuz, I. & Schlegel, W. (2007) Gene-specific recruitment of positive and negative elongation factors during stimulated transcription of the MKP-1 gene in neuroendocrine cells. *Nucleic Acids Res.* **35**, 1007-1017.

Fujita, T., Yuno, M. & Fujii, H. (2016a) Allele-specific locus binding and genome editing by CRISPR at the p16INK4a locus. *Sci. Rep.* **6**, 30485.

Fujita, T., Yuno, M. & Fujii, H. (2016b) Efficient sequence-specific isolation of DNA fragments and chromatin by in vitro enChIP technology using recombinant CRISPR ribonucleoproteins. *Genes Cells* **21**, 370-377.

Fujita, T., Yuno, M., Okuzaki, D., Ohki, R. & Fujii, H. (2015b) Identification of non-coding RNAs associated with telomeres using a combination of enChIP and RNA sequencing. *PLoS One* **10**, e0123387.

Gavrilov, A.A., Chetverina, H.V., Chermnykh, E.S., Razin, S.V. & Chetverin, A.B. (2014) Quantitative analysis of genomic element interactions by molecular colony technique. *Nucleic Acids Res.* **42**, e36.

Gavrilov, A.A., Gushchanskaya, E.S., Strelkova, O., Zhironkina, O., Kireev, I.I., Iarovaia, O.V. & Razin, S.V. (2013) Disclosure of a structural milieu for the proximity ligation reveals the elusive nature of an active chromatin hub. *Nucleic Acids Res.* **41**, 3563-3575.

Ghirlando, R., Giles, K., Gowher, H., Xiao, T., Xu, Z., Yao, H. & Felsenfeld, G. (2012) Chromatin domains, insulators, and the regulation of gene expression. *Biochim. Biophys. Acta* **1819**.

Holwerda, S.J. & de Laat, W. (2013) CTCF: the protein, the binding partners, the binding sites and their chromatin loops. *Philos. Trans. R. Soc. Lond. B. Biol. Sci.* **368**, 20120369.

Hoshino, A. & Fujii, H. (2009) Insertional chromatin immunoprecipitation: a method for isolating specific genomic regions. *J. Biosci. Bioeng.* **108**, 446-449.

Kalhor, R., Tjong, H., Jayathilaka, N., Alber, F. & Chen, L. (2011) Genome architectures revealed by tethered chromosome conformation capture and population-based modeling. *Nat Biotechnol.* **30**, 90-98.

Kuscu, C., Arslan, S., Singh, R., Thorpe, J. & Adli, M. (2014) Genome-wide analysis reveals characteristics of off-target sites bound by the Cas9 endonuclease. *Nat Biotechnol.* **32**, 677-683.

Mali, P., Yang, L., Esvelt, K.M., Aach, J., Guell, M., DiCarlo, J.E., Norville, J.E. & Church, G.M. (2013) RNA-guided human genome engineering via Cas9. *Science* **339**, 823-826.

Miller, A.D. & Buttimore, C. (1986) Redesign of retrovirus packaging cell lines to avoid recombination leading to helper virus production. *Mol Cell Biol.* **6**, 2895-2902.

Nagano, T., Várnai, C., Schoenfelder, S., Javierre, B.M., Wingett, S.W. & Fraser, P. (2015) Comparison of Hi-C results using in-solution versus in-nucleus ligation. *Genome Biol.* **16**, 175.

Navas, P.A., Peterson, K.R., Li, Q., McArthur, M. & Stamatoyannopoulos, G. (2001) The 5'HS4 core element of the human beta-globin locus control region is required for high-level globin gene expression in definitive but not in primitive erythropoiesis. *J. Mol. Biol.* **312**, 17-26.

O'Green, H., Henry, I.M., Bhakta, M.S., Meckler, J.F. & Segal, D.J. (2015) A genome-wide analysis of Cas9 binding specificity using ChIP-seq and targeted sequence capture. *Nucleic Acids Res.* **43**, 3389-3404.

Pabo, C.O., Peisach, E. & Grant, R.A. (2001) Design and selection of novel Cys2his2 zinc finger poroteins. *Annu. Rev. Biochem.* **70**, 313-340.

Peterson, K.R., Clegg, C.H., Navas, P.A., Norton, E.J., Kimbrough, T.G. & Stamatoyannopoulos, G. (1996) Effect of deletion of 5'HS3 or 5'HS2 of the human beta-globin locus control region on the developmental regulation of globin gene expression in beta-globin locus yeast artificial chromosome transgenic mice. *Proc. Natl. Acad. Sci. U. S. A.* **93**, 6605-6609.

Qi, L.S., Larson, M.H., Gilbert, L.A., Doudna, J.A., Weissman, J.S., Arkin, A.P. & Lim, W.A. (2013) Repurposing CRISPR as an RNA-guided platform for sequence-specific control of gene expression. *Cell* **152**, 1173-1183.

Seki, M., Masaki, H., Arauchi, T., Makauchi, H., Sugano, S. & Suzuki, Y. (2014) A comparison of the Rest complex binding patterns in embryonic stem cells and epiblast stem cells. *PLoS One* **9**, e95374.

Williams, A., Spilianakis, C.G. & Flavell, R.A. (2010) Interchromosomal association and gene regulation in trans. *Trends Genet.* **26**, 188-197.

Williamson, I., Berlivet, S., Eskeland, R., Boyle, S., Illingworth, R.S., Paquette, D., Dostie, J. & Bickmore, W.A. (2014) Spatial genome organization: contrasting views from chromosome conformation capture and fluorescence in situ hybridization. *Genes Dev.* **28**, 2778-2791.

Wu, X., Scott, D.A., Kriz, A.J., Chiu, A.C., Hsu, P.D., Dadon, D.B., Cheng, A.W., Trevino, A.E., Konermann, S., Chen, S., Jaenisch, R., Zhang, F. & Sharp, P.A. (2014) Genome-wide binding of the CRISPR endonuclease Cas9 in mammalian cells. *Nat. Biotechnol.* **32**, 670-676.

Yamashita, R., Sathira, N.P., Kanai, A., Tanimoto, K., Arauchi, T., Tanaka, Y., Hashimoto, S., Sugano, S., Nakai, K. & Suzuki, Y. (2011) Genome-wide characterization of transcriptional start sites in humans by integrative transcriptome analysis. *Genome Res* **21**, 775-789.

Figure legends

Fig. 1. **Schematic of the use of enChIP-Seq analysis to identify physical interactions between genomic regions.** (i) The CRISPR system for locus tagging. It consists of 3xFLAG-dCas9 (a fusion protein of the 3xFLAG-tag, dCas9, and a nuclear localization signal [NLS]) and a single guide RNA (sgRNA) targeting the locus of interest. (ii) 3xFLAG-dCas9 and the sgRNA are expressed for locus tagging in the cells to be analyzed. (iii) The cells are crosslinked, if necessary, and lysed. Chromatin is purified and fragmented by sonication or other methods. (iv) Complexes containing the CRISPR complex are immunoprecipitated with anti-FLAG Ab. (v) After reversal of crosslinking, if necessary, DNA is purified and subjected to the NGS analysis to identify interacting genomic regions (blue line). In this scheme, the CRISPR system is shown as a representative. Other engineered DNA-binding molecules including ZF and TAL proteins are also suitable for use in enChIP-Seq.

Fig. 2. **Isolation of the 5'HS5 locus by enChIP.** (*A*) Schematic depiction of the *β-globin* locus. The positions of the 5'HS and 3'HS1 loci and the *globin* genes are indicated. (*B*) Positions of sgRNA target sites. Purple, CTCF-binding site (Farrell *et al.* 2002); red, 5'HS5 core region (NCBI Reference Sequence: NG_000007.3); green, sgRNA target site (5'HS5 #6); blue, sgRNA target site (5'HS5 #17); italic and underline, primer positions used in enChIP-real-time PCR in (**c**). (*C*) Yields of enChIP for the 5'HS5 locus. (*D* and *E*) NGS peaks at the target 5'HS5 locus (*D*) and the irrelevant *SOX2* locus (*E*). Raw ChIP-Seq read data were displayed as density plots in the UCSC Genome Browser. The vertical viewing range (*y*-axis shown as Scale) was set at 1-300 based on the magnitude of the noise peaks. Black vertical bars show locus positions in the human

genome (hg19 assembly). The position of the *SOX2* gene is shown under the plot (*E*). (-), mock-treated; NaB, treated with sodium butyrate.

Fig. 3. **Filtering of the NGS peaks to identify genomic regions that interact with the 5'HS5 locus in the differentiated state.** (**Step 1**) Extraction of enChIP-specific NGS peaks by comparison with enChIP and input peaks detected in the differentiated state, using Model-based Analysis of ChIP-Seq (MACS) (http://liulab.dfci.harvard.edu/MACS/). The extracted enChIP-specific peaks include NaB-specific and constitutively detected peaks. (**Step 2**) Elimination of peaks derived from non-specific binding of dCas9. After the elimination step, peaks fulfilling the defined criteria (19 for sgRNA #6 and 228 for sgRNA #17) were analyzed in step 3. (**Step 3**) Identification of peaks detected in common by both sgRNA #6 and #17. Genomic regions corresponding to the identified peaks were considered to physically interact with the 5'HS5 locus. (**Step 4**) Extraction of enChIP-specific NGS peaks by comparison with enChIP and input peaks detected in the undifferentiated state, using MACS. The extracted enChIP-specific peaks include peaks specifically detected in undifferentiated cells and constitutively detected peaks. (**Step 5**) Identification of genomic regions that interact with the 5'HS5 locus in an NaB-specific manner.

Fig. 4. Identification of genomic regions that physically interact with the 5'HS5 locus by enChIP-Seq. Raw enChIP-Seq read data were displayed as density plots in the UCSC Genome Browser. The vertical viewing range (*y*-axis shown as Scale) was set at 1-160 based on the noise peaks. Black vertical bars reveal locus positions in the human genome (hg19 assembly). Positions of genes are shown under the plots. (-), mock-treated; NaB, treated with sodium butyrate.

Fig. 5. Confirmation of enChIP-Seq results by the ligation-mediated assay. Results of PCR. K562 cells cultured in the presence or absence of NaB were crosslinked, and the chromatin was extracted, fragmented, and subjected to the proximity ligation assay (Supplementary Fig. S2) to confirm interaction between the 5'HS5 and the loci detected by enChIP-Seq. The loci in chromosome 1 (*ZNF670/ZNF670-ZNF695*) (*A*), chromosome 15 (*MIR422A*) (*B*), and chromosome 11 (*YIF1A*) (*C*) were detected by the second PCR. Amplification in the second PCR suggests that 5'HS5 is physically proximal to the loci detected by enChIP-Seq. Two representatives (#1 and #2) of each sample (±NaB treatment) are shown. See also Supplementary Fig. S3.

Fig. 6. NaB-induced expression of genes in the vicinity of genomic regions that interact with the 5'HS5 locus. Total RNA was extracted from K562 and used for quantitative RT-PCR analysis. Expression levels of the indicated genes were normalized to those of *GAPDH*, and the levels of mRNA in the absence of NaB were defined as 1 (mean ± SD, n = 3). N.D., not detected; *, t-test p value <0.05; **, t-test p value <0.01.

Fig. 7. Detection of chromosomal interactions between the 5'HS5 locus and the *globin* genes (*HBG1* and *HBG2*) at the *β-globin* locus by enChIP-Seq. enChIP-Seq reads corresponding to *HBG1* and *HBG2* genes (± 0.5 kb around each TSS, Supplementary Fig. S5) were manually counted and fold enrichment with respect to input reads was analyzed. The data are shown in Supplementary Table S3.

Table 1. NGS information regarding the 5'HS5 target locus.

sgRNA	Condition	Fold enrichment (compared with input gDNA)*	Number of tags†
#6	minus (mock)	120	655
	NaB	168	783
#17	minus (mock)	65	273
	NaB	122	217

*, NGS tag numbers of the target 5'HS5 locus were compared between enChIP-Seq and input genomic DNA samples and fold enrichment relative to input genomic DNA calculated by the MACS2 software is shown.

†, NGS tag numbers of the target 5'HS5 locus detected by enChIP-Seq.

Table 2. List of peak positions detected in common by both sgRNA #6 and #17.

sgRNA #6 (NaB)						sgRNA #17 (NaB)						
chr	start	end	length (base)	tags§	fold enrichment†	chr	start	end	length (base)	tags§	fold enrichment†	status‡
chr1	247240307	247240894	588	68	17.62	chr1	247240610	247240804	195	18	12.84	NaB-specific
chr1	247240925	247241339	415	49	12.95	chr1	247240971	247241324	354	28	10.33	NaB-specific
chr7	132001863	132002364	502	71	27.24	chr7	132001929	132002385	457	50	22.29	NaB-specific
chr11	66057056	66057342	287	45	30.65	chr11	66057120	66057297	178	13	14.48	NaB-specific
chr15	64162769	64163183	415	51	29.06	chr15	64162762	64163061	300	31	27.51	NaB-specific
chr11*	5311586	5312647	1062	783	168.11	chr11	5311878	5312599	722	217	122.27	constitutive

The peak positions detected in Step 5 in Fig. 3 are shown.

*, target locus (5'HS5)

§, the NGS tag numbers of the peak positions detected in enChIP-Seq.

†, the NGS tag numbers of peak positions were compared between enChIP-Seq and input genomic DNA samples and fold enrichment relative to input genomic DNA calculated by the MACS2 software is shown.

‡, the peak positions were detected constitutively or in an NaB-specific manner by enChIP-Seq.

Figure 1

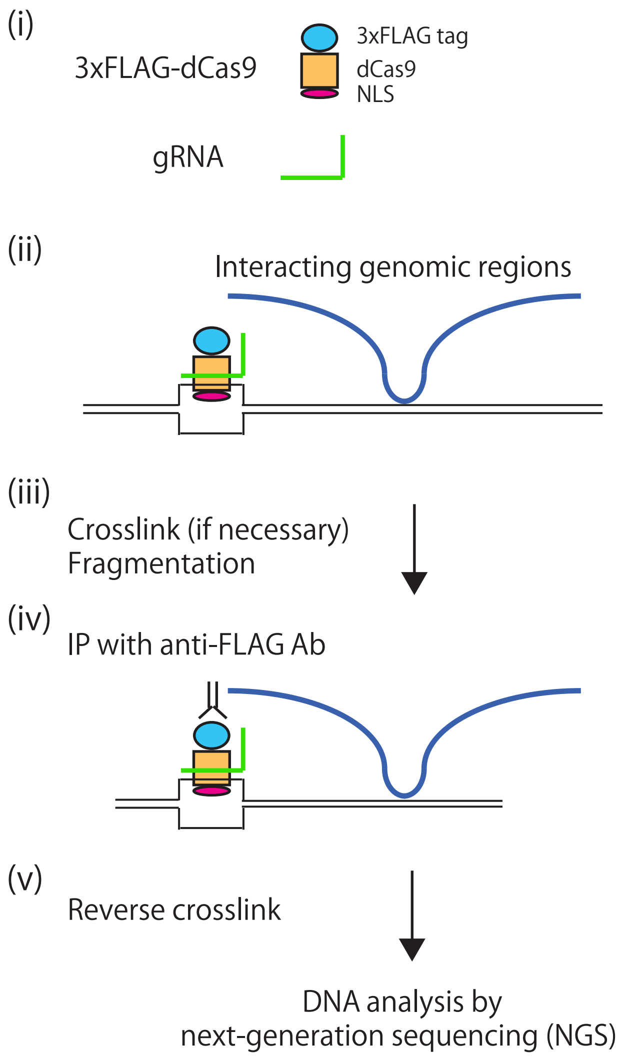
(i)
3xFLAG-dCas9
3xFLAG tag
dCas9
NLS
gRNA
(ii)
Interacting genomic regions
(iii)
Crosslink (if necessary)
Fragmentation
(iv)
IP with anti-FLAG Ab
(v)
Reverse crosslink
DNA analysis by
next-generation sequencing (NGS)

Figure 2

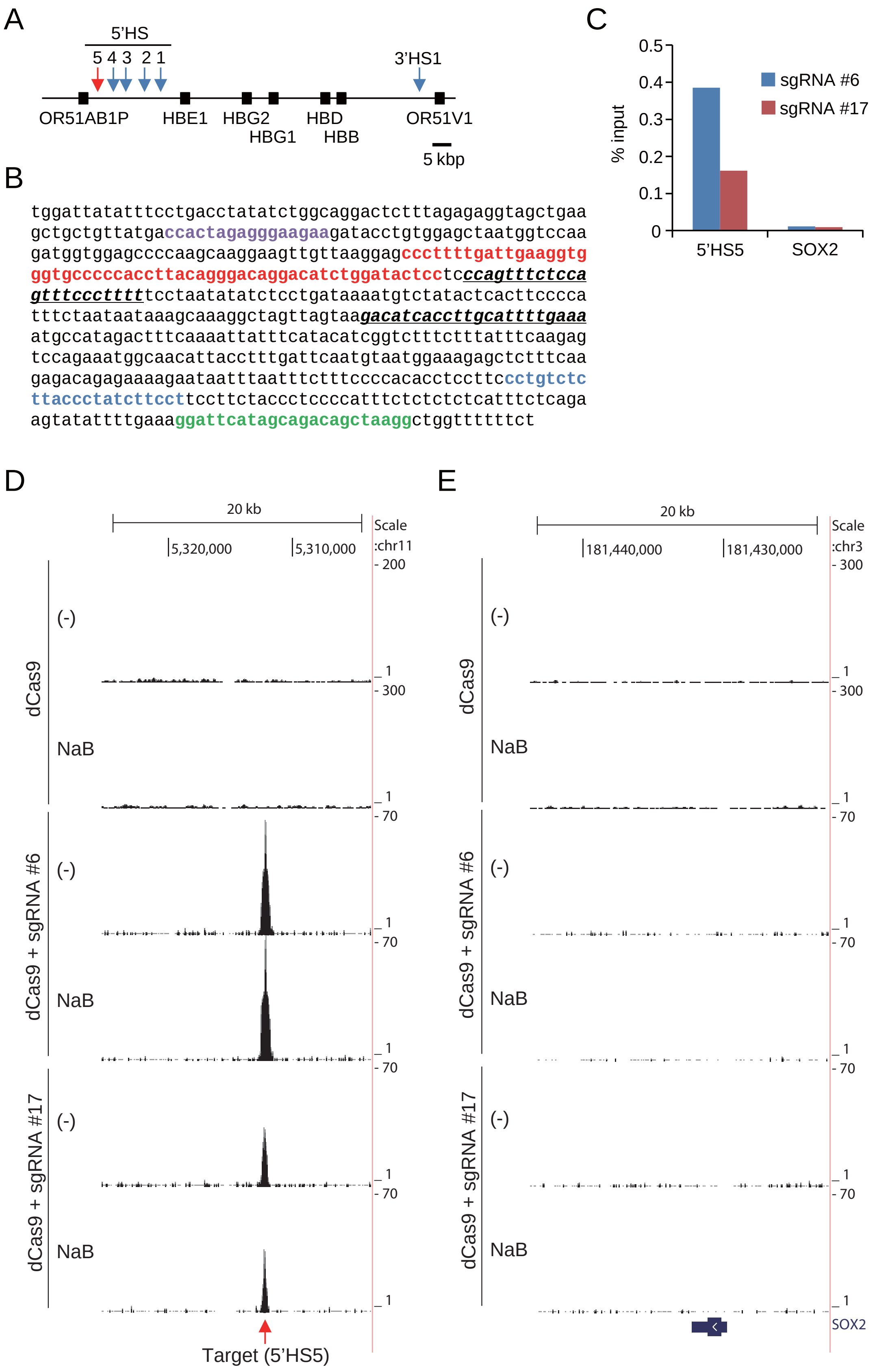
A
5'HS
5 4 3 2 1
3'HS1
OR51AB1P
HBE1
HBG2
HBG1
HBD
HBB
OR51V1
5 kbp
B
tggattatatttcctgacctatatctggcaggactctttagagaggtagctgaa
gctgctgttatgaccactagagggaagaagatacctgtggagctaatggtccaa
gatggtggagccccaagcaaggaagttgttaaggagccctttttgattgaaggtg
ggtgcccccaccttacagggacaggacatctggatactcctcccagtttctcca
gtttcccttttttcctaatatatctcctgataaaatgtctatactcacttcccca
tttctaataataaagcaaaggctagttagtaagacatcaccttgcattttgaaa
atgccatagactttcaaaattatttcatacatcggtctttctttatttcaagag
tccagaaatggcaacattacctttgattcaatgtaatggaaagagctctttcaa
gagacagagaaaagaataatttaatttctttccccacacctccttccctgtctc
ttaccctatcttccttccttctaccctccccatttctctctctcatttctcaga
agtatattttgaaaggattcatagcagacagctaaggctggtttttttct
C
% input
0.5
0.4
0.3
0.2
0.1
0
sgRNA #6
sgRNA #17
5'HS5
SOX2
D
20 kb
5,320,000
5,310,000
Scale
:chr11
- 200
_ 1
- 300
_ 1
- 70
_ 1
- 70
_ 1
- 70
_ 1
- 70
_ 1
dCas9
(-)
NaB
dCas9 + sgRNA #6
(-)
NaB
dCas9 + sgRNA #17
(-)
NaB
Target (5'HS5)
E
20 kb
181,440,000
181,430,000
Scale
:chr3
- 300
_ 1
- 300
_ 1
- 70
_ 1
- 70
_ 1
- 70
_ 1
- 70
_ 1
dCas9
(-)
NaB
dCas9 + sgRNA #6
(-)
NaB
dCas9 + sgRNA #17
(-)
NaB
SOX2

Figure 3

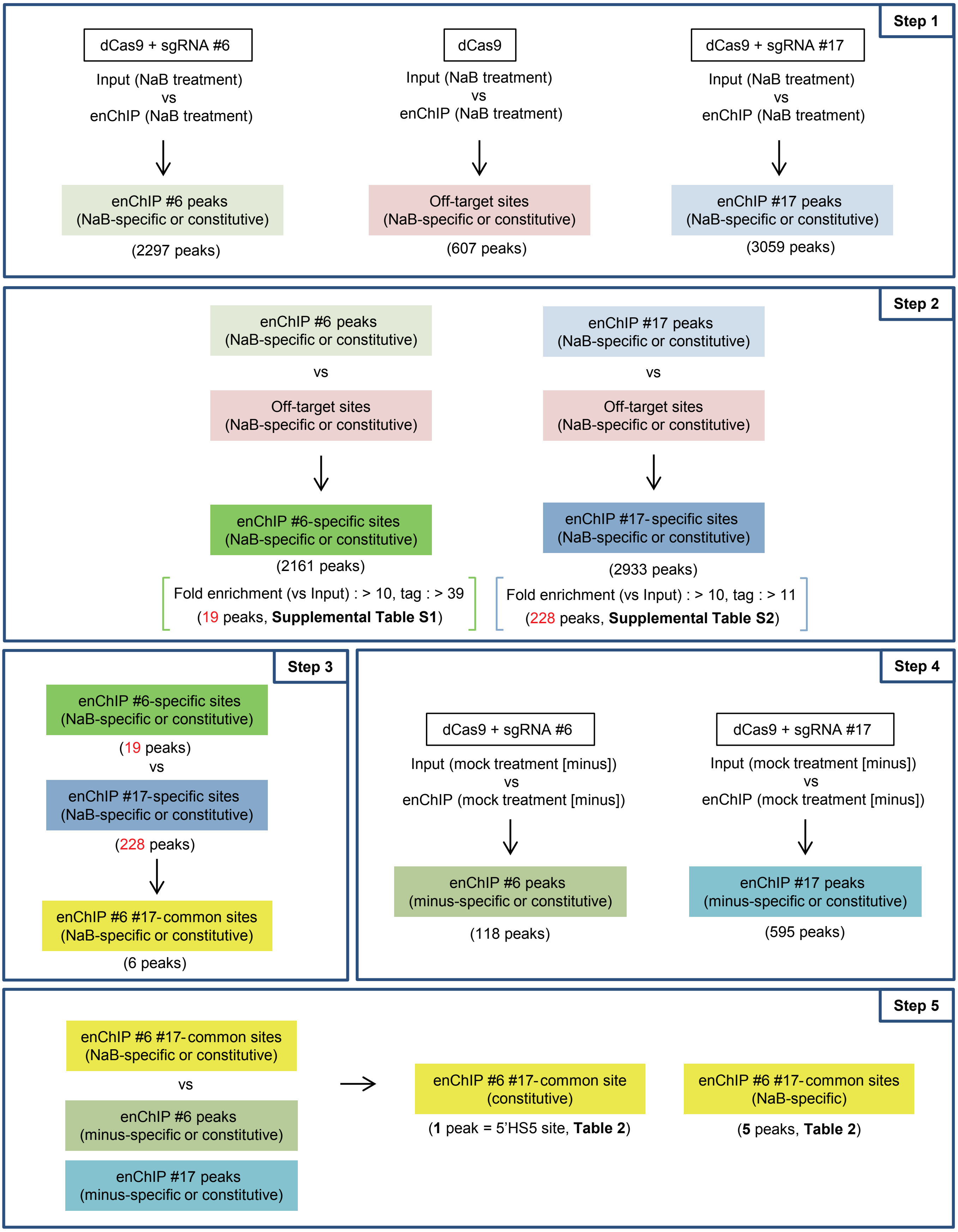

Step 1
dCas9 + sgRNA #6
Input (NaB treatment)
vs
enChIP (NaB treatment)
enChIP #6 peaks
(NaB-specific or constitutive)
(2297 peaks)
dCas9
Input (NaB treatment)
vs
enChIP (NaB treatment)
Off-target sites
(NaB-specific or constitutive)
(607 peaks)
dCas9 + sgRNA #17
Input (NaB treatment)
vs
enChIP (NaB treatment)
enChIP #17 peaks
(NaB-specific or constitutive)
(3059 peaks)
Step 2
enChIP #6 peaks
(NaB-specific or constitutive)
vs
Off-target sites
(NaB-specific or constitutive)
enChIP #6-specific sites
(NaB-specific or constitutive)
(2161 peaks)
Fold enrichment (vs Input) : > 10, tag : > 39
(19 peaks, Supplemental Table S1)
enChIP #17 peaks
(NaB-specific or constitutive)
vs
Off-target sites
(NaB-specific or constitutive)
enChIP #17-specific sites
(NaB-specific or constitutive)
(2933 peaks)
Fold enrichment (vs Input) : > 10, tag : > 11
(228 peaks, Supplemental Table S2)
Step 3
enChIP #6-specific sites
(NaB-specific or constitutive)
(19 peaks)
vs
enChIP #17-specific sites
(NaB-specific or constitutive)
(228 peaks)
enChIP #6 #17-common sites
(NaB-specific or constitutive)
(6 peaks)
Step 4
dCas9 + sgRNA #6
Input (mock treatment [minus])
vs
enChIP (mock treatment [minus])
enChIP #6 peaks
(minus-specific or constitutive)
(118 peaks)
dCas9 + sgRNA #17
Input (mock treatment [minus])
vs
enChIP (mock treatment [minus])
enChIP #17 peaks
(minus-specific or constitutive)
(595 peaks)
Step 5
enChIP #6 #17-common sites
(NaB-specific or constitutive)
vs
enChIP #6 peaks
(minus-specific or constitutive)
enChIP #17 peaks
(minus-specific or constitutive)
enChIP #6 #17-common site
(constitutive)
(1 peak = 5'HS5 site, Table 2)
enChIP #6 #17-common sites
(NaB-specific)
(5 peaks, Table 2)

Figure 4

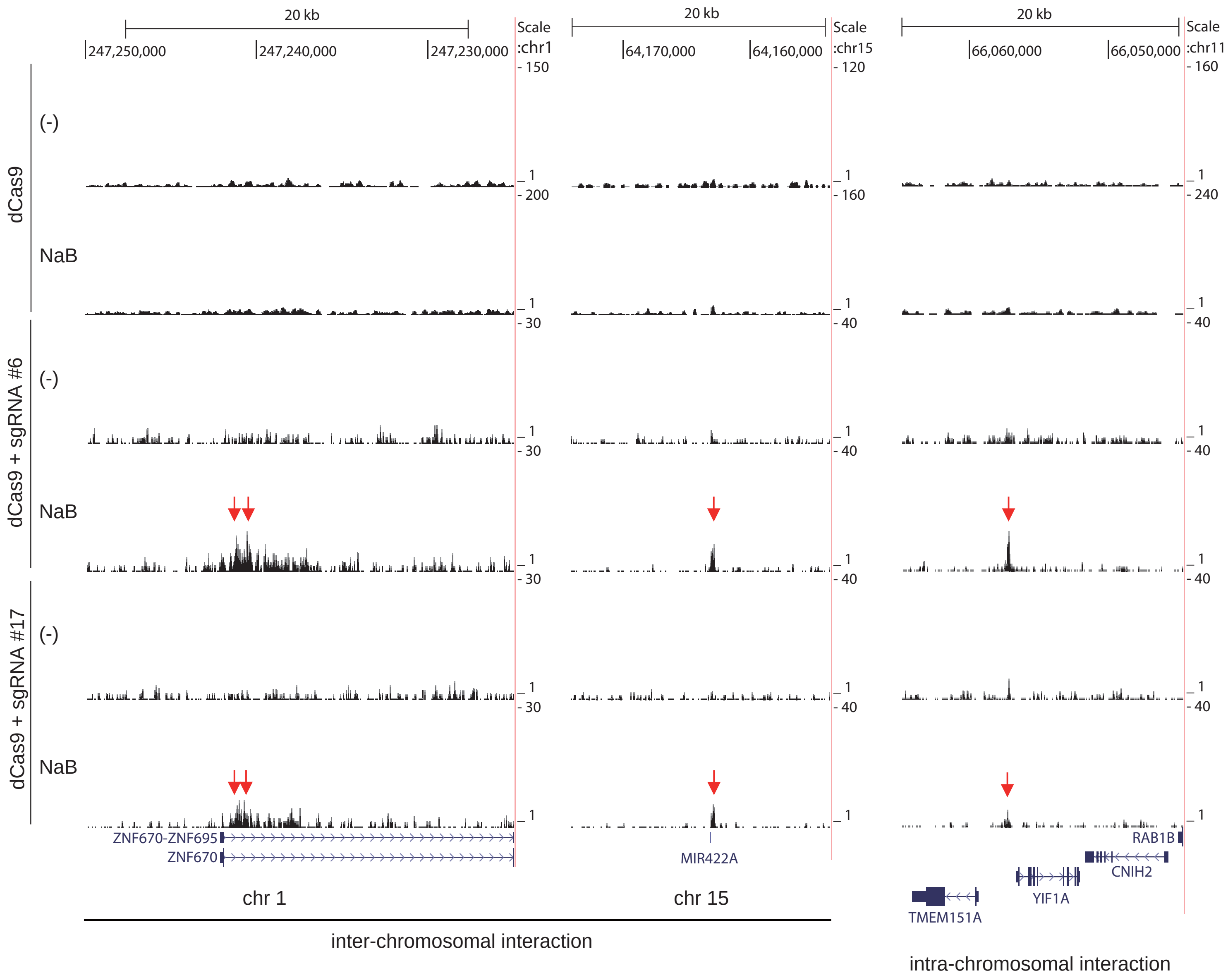
20 kb
Scale
:chr1
247,250,000
247,240,000
247,230,000
:chr15
64,170,000
64,160,000
:chr11
66,060,000
66,050,000
dCas9
dCas9 + sgRNA #6
dCas9 + sgRNA #17
(-)
NaB
ZNF670-ZNF695
ZNF670
MIR422A
RAB1B
CNIH2
YIF1A
TMEM151A
chr 1
chr 15
inter-chromosomal interaction
intra-chromosomal interaction

Figure 5

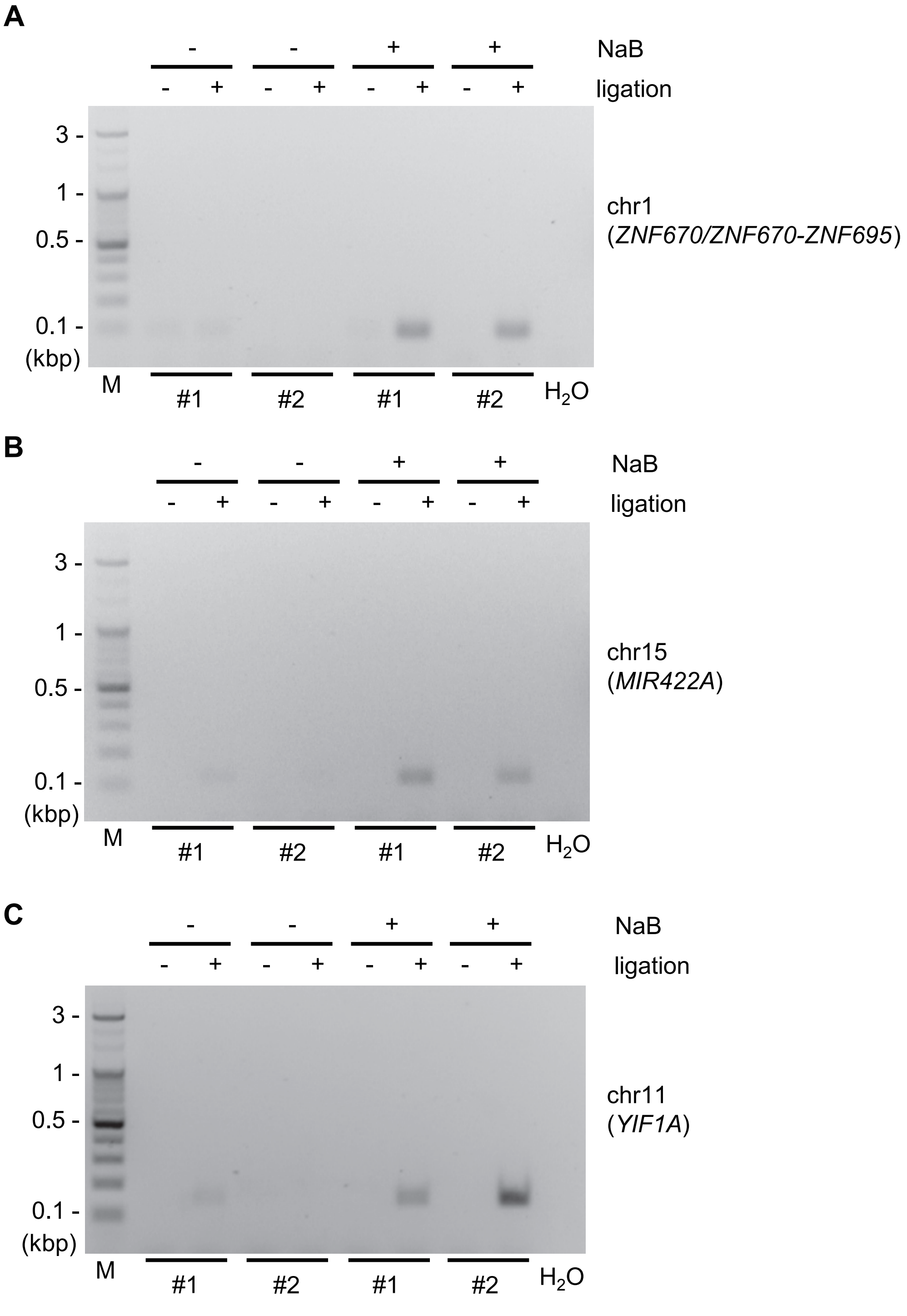
A
NaB
ligation
3 -
1 -
0.5 -
0.1 -
(kbp)
M
#1
#2
#1
#2
H2O
chr1
(ZNF670/ZNF670-ZNF695)
B
NaB
ligation
chr15
(MIR422A)
C
NaB
ligation
chr11
(YIF1A)

Figure 6

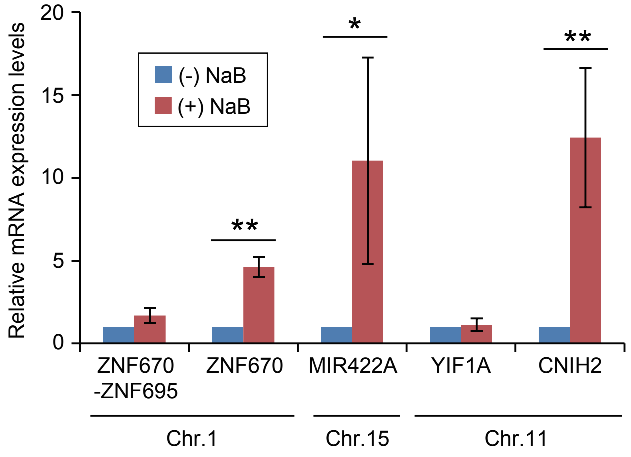

Relative mRNA expression levels
20
15
10
5
0
(-) NaB
(+) NaB
*
**
**
ZNF670
-ZNF695
ZNF670
MIR422A
YIF1A
CNIH2
Chr.1
Chr.15
Chr.11

Figure 7

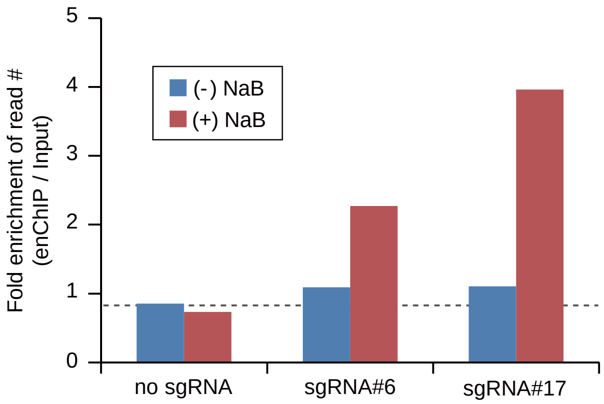

Fold enrichment of read #
(enChIP / Input)
5
4
3
2
1
0
(-) NaB
(+) NaB
no sgRNA
sgRNA#6
sgRNA#17

Supplementary Information

Identification of physical interactions between genomic regions by enChIP-Seq

Toshitsugu Fujita, Miyuki Yuno, Yutaka Suzuki, Sumio Sugano, and Hodaka Fujii

Supplementary Figure Legends

Supplementary Fig. S1. Filtering of NGS peaks to identify genomic regions that interact with the 5'HS5 locus in the undifferentiated state. (**Step 1**) Extraction of genomic regions that interact with the 5'HS5 locus specifically in the undifferentiated state. After the extraction step, regions fulfilling the defined criteria (one for sgRNA #6 and seven for sgRNA #17) were analyzed in Step 2. (**Step 2**) Identification of peaks detected in common using sgRNA #6 and #17.

Supplementary Fig. S2. Scheme for Detection of DNA-DNA interactions (D-Cube), a ligation-mediated assay, to confirm interactions between genomic regions. Cells are crosslinked and lysed, and genomic DNA is randomly fragmented by sonication or other methods. After repair of DNA ends, proximal ligation, and reversal of crosslinking, the junction between the target locus and its potential interacting region is amplified by PCR. If a biotinylated primer is used, the amplicon can be purified using streptavidin. Subsequently, a part of the amplified region in the potential interacting locus is detected by the second PCR. Amplification of the region in the second PCR suggests that the potential interacting locus is physically proximal to the target locus.

Supplementary Fig. S3. Confirmation of the enChIP-Seq results by the ligation-mediated assay. The PCR results obtained using the DNA for the first PCR and the locus-specific primer sets used for the second PCR are shown. The loci in chromosome 1 (*ZNF670/ZNF670-ZNF695*) (**A**), chromosome 15 (*MIR422A*) (**B**), and chromosome 11 (*YIF1A*) (**C**) were detected. These results show that the assays were run with comparable amounts of input DNA. Two representatives (#1 and #2) of each sample (±NaB treatment) are shown.

Supplementary Fig. S4. NGS peak images in the *β-globin* locus. Raw ChIP-Seq read data were displayed as density plots in the UCSC Genome Browser. The vertical viewing range (*y*-axis shown as Scale) was set at 1-300, based on the magnitude of the noise peaks. Black vertical bars show locus positions in the human genome (hg19 assembly). Positions of genes, including *globin* genes, are shown under the plots.

Supplementary Fig. S5. Identities of the DNA sequences between human *HBG1* and *HBG2* genes. (**Upper**) Sequence identity was analyzed in the gene bodies and upstream regions (from -1 kb to -0.5 kb and form -0.5 kb to -1). (**Lower**) The results of a comparison of DNA sequences around the TSSs (± 0.5 kb) are shown. The NGS reads corresponding to these genomic regions were manually counted.

Supplementary Fig. S6. enChIP-Seq did not show chromosomal interactions between the 5'HS5 locus and irrelevant loci. enChIP-Seq reads corresponding to *IFNG* and *ACTA1* genes (± 0.5 kb around TSSs) were manually counted and fold enrichment with respect to input reads was analyzed. The detailed data are shown in Supplementary Table S4.

Supplementary Table S1. List of genomic regions detected in the differentiated state (sgRNA #6) by enChIP-Seq.

<table>
<tr><th colspan="11">sgRNA #6 (NaB-specific and constitutive peaks)</th></tr>
<tr><th>chr</th><th>start</th><th>end</th><th>length</th><th>summit</th><th>tags</th><th>-10*LOG10(pvalue)</th><th>fold_enrichment</th><th>FDR(%)</th><th>gene1</th><th>gene2</th></tr>
<tr><td>chr1</td><td>33526002</td><td>33526540</td><td>539</td><td>188</td><td>95</td><td>869.16</td><td>51.67</td><td>0</td><td>.</td><td>.</td></tr>
<tr><td>chr1</td><td>153586701</td><td>153587167</td><td>467</td><td>81</td><td>49</td><td>259.09</td><td>15.83</td><td>0</td><td>.</td><td>1654:NM_080388:S100A16|-1640:NM_020672:S100A14|</td></tr>
<tr><td>chr1</td><td>247240307</td><td>247240894</td><td>588</td><td>246</td><td>68</td><td>322.89</td><td>17.62</td><td>0</td><td>.</td><td>-1220:NM_001204220,NM_033213:ZNF670|</td></tr>
<tr><td>chr1</td><td>247240925</td><td>247241339</td><td>415</td><td>243</td><td>49</td><td>221.87</td><td>12.95</td><td>0</td><td>.</td><td>-775:NM_001204220,NM_033213:ZNF670|</td></tr>
<tr><td>chr10</td><td>15387617</td><td>15388319</td><td>703</td><td>369</td><td>382</td><td>3100</td><td>101.06</td><td>0</td><td>.</td><td>.</td></tr>
<tr><td>chr11</td><td>5311586</td><td>5312647</td><td>1062</td><td>595</td><td>783</td><td>3100</td><td>168.11</td><td>0</td><td>.</td><td>.</td></tr>
<tr><td>chr11</td><td>36122948</td><td>36123372</td><td>425</td><td>281</td><td>63</td><td>441.98</td><td>27.79</td><td>0</td><td>.</td><td>.</td></tr>
<tr><td>chr11</td><td>57669714</td><td>57670066</td><td>353</td><td>129</td><td>42</td><td>199.98</td><td>11.97</td><td>0</td><td>.</td><td>.</td></tr>
<tr><td>chr11</td><td>66057056</td><td>66057342</td><td>287</td><td>127</td><td>45</td><td>329.48</td><td>30.65</td><td>0</td><td>2317:NM_153266:TMEM151A|</td><td>705:NM_020470:YIF1A|</td></tr>
<tr><td>chr11</td><td>66131388</td><td>66131805</td><td>418</td><td>189</td><td>56</td><td>388.52</td><td>33.37</td><td>0</td><td>.</td><td>-7485:NM_001532:SLC29A2|</td></tr>
<tr><td>chr12</td><td>76347754</td><td>76348240</td><td>487</td><td>266</td><td>76</td><td>513.86</td><td>22.08</td><td>0</td><td>.</td><td>.</td></tr>
<tr><td>chr15</td><td>64162769</td><td>64163183</td><td>415</td><td>80</td><td>51</td><td>400.95</td><td>29.06</td><td>0</td><td>.</td><td>.</td></tr>
<tr><td>chr16</td><td>1359061</td><td>1359401</td><td>341</td><td>284</td><td>41</td><td>299.03</td><td>25.24</td><td>0</td><td>93:NM_003345,NM_194259,NM_194260,NM_194261:UBE2I|</td><td>.</td></tr>
<tr><td>chr16</td><td>67522986</td><td>67523488</td><td>503</td><td>265</td><td>110</td><td>863.92</td><td>38.37</td><td>0</td><td>.</td><td>8400:NM_004691:ATP6V0D1|5773:NM_001138:AGRP|</td></tr>
<tr><td>chr4</td><td>81093373</td><td>81093786</td><td>414</td><td>190</td><td>46</td><td>266.82</td><td>19.07</td><td>0</td><td>.</td><td>.</td></tr>
<tr><td>chr5</td><td>118784853</td><td>118785151</td><td>299</td><td>163</td><td>44</td><td>318.58</td><td>31.93</td><td>0</td><td>3285:NM_000414,NM_001199291,NM_001199292:HSD17B4|</td><td>.</td></tr>
<tr><td>chr6</td><td>116781574</td><td>116781978</td><td>405</td><td>138</td><td>41</td><td>208.29</td><td>13.35</td><td>0</td><td>982:NM_001010919:FAM26F|</td><td>.</td></tr>
<tr><td>chr7</td><td>132001863</td><td>132002364</td><td>502</td><td>272</td><td>71</td><td>488.49</td><td>27.24</td><td>0</td><td>.</td><td>.</td></tr>
<tr><td>chr9</td><td>108639933</td><td>108640362</td><td>430</td><td>283</td><td>41</td><td>171.13</td><td>14.3</td><td>0</td><td>.</td><td>.</td></tr>
</table>

Common (sgRNA #6 and #17)

Target site

Supplementary Table S2. List of genomic regions commonly detected in the differentiated states (sgRNA #17) by enChIP-Seq.

sgRNA #17 (NaB-specific and constitutive peaks)										
chr	start	end	length	summit	tags	-10*LOG10(pvalue)	fold_enrichment	FDR(%)	gene1	gene2
chr1	11919466	11919666	201	155	16	106.97	21.5	0.62	.	675:NM_002521:NPPB|
chr1	12316588	12316738	151	51	11	61.36	11.46	1.52	.	.
chr1	12316760	12316874	115	56	11	61.65	14.06	1.46	.	.
chr1	16840452	16840625	174	129	28	105.1	12.77	0.61	.	.
chr1	25119803	25119904	102	65	11	87.09	18.88	1.02	.	.
chr1	26744588	26744690	103	56	11	90.74	26.45	0.81	-7319:NM_024674:LIN28A|	.
chr1	45187527	45187664	138	66	12	88.76	21.4	0.74	.	.
chr1	47201499	47201650	152	64	12	87.34	19.65	1.04	.	.
chr1	61511409	61511491	83	54	12	90.53	19.03	0.78	.	.
chr1	65583464	65583638	175	38	13	79.36	13.49	0.98	.	.
chr1	76238395	76238528	134	70	11	89.52	22.37	0.76	.	.
chr1	111289435	111289681	247	97	19	91.87	10.42	0.87	.	.
chr1	111492829	111493061	233	52	25	131.81	17.64	0	.	.
chr1	114755922	114756032	111	31	12	88.62	22.07	0.74	.	.
chr1	115300292	115300460	169	77	13	81.86	10.92	1.13	.	-210:NM_001007553,NM_001130523,NM_001242891,NM_001242892,NM_001242893,NM_007158:CSDE1|
chr1	118377797	118377896	100	33	16	154.8	30.77	0	.	.
chr1	118448119	118448224	106	32	11	83.6	18.24	1.21	.	.
chr1	118448271	118448398	128	33	11	75.27	15.81	0.83	.	.
chr1	119098643	119098812	170	114	16	88.27	17.54	0.72	.	.
chr1	119098833	119098996	164	92	12	76.75	17.19	0.89	.	.
chr1	142812735	142812886	152	55	15	56.75	11.62	1.62	.	.
chr1	154955985	154956103	119	42	12	68.62	14.11	1.15	-215:NM_001184891,NM_001184892,NM_025207,NM_201398:FLAD1|10077:NM_018655:LENEP|-8867:NM_001826:CKS1B|	9145:NM_001130040,NM_001130041,NM_001202859,NM_003029,NM_183001:SHC1|
chr1	163151931	163152050	120	22	15	129.81	21.4	0	.	.
chr1	165366980	165367170	191	96	14	83.95	16.03	1.23	.	.
chr1	176760594	176760706	113	56	17	145.97	25.91	0	.	.
chr1	182156036	182156175	140	66	12	84.73	25.79	1.28	.	.
chr1	185033362	185033478	117	55	12	70.13	12.52	0.92	.	.
chr1	190281875	190281997	123	52	12	79.99	16.05	1.01	.	.
chr1	202662235	202662452	218	75	20	117.16	13.76	0	.	.
chr1	206600619	206600731	113	29	12	98.07	20.98	1.05	.	.
chr1	207682410	207682546	137	21	13	83.77	16.05	1.22	.	.
chr1	209779349	209779484	136	66	11	80.31	21.83	1.02	.	.
chr1	215441144	215441251	108	33	15	158.35	42.46	0	.	.
chr1	224278844	224278981	138	75	11	63.66	16.18	1.23	.	.
chr1	224659181	224659306	126	60	12	82.98	17.44	1.17	.	.
chr1	227498113	227498241	129	61	12	70.13	11.83	0.92	.	-7584:NM_003607,NM_014826:CDC42BPA|
chr1	230233766	230233849	84	30	15	97.35	14.12	1.02	.	.
chr1	231064213	231064322	110	83	12	100.86	21.54	1.09	.	.
chr1	233764914	233765193	280	171	23	117.81	15.28	0	.	.
chr1	235028303	235028404	102	24	12	88.24	17.89	0.72	.	.
chr1	242123452	242123574	123	30	15	66.9	11.28	1.07	.	.
chr1	247239197	247239370	174	79	12	61.3	11.08	1.52	.	-2744:NM_001204220,NM_033213:ZNF670|
chr1	247240610	247240804	195	110	18	91.65	12.84	0.85	.	-1310:NM_001204220,NM_033213:ZNF670|
chr1	247240971	247241324	354	67	28	95.69	10.33	0.98	.	-790:NM_001204220,NM_033213:ZNF670|
chr10	38811337	38811453	117	87	23	116.01	11.62	0	.	.
chr10	51531252	51531379	128	47	11	71.68	17.07	1.04	.	.
chr10	62735764	62735872	109	32	11	77.69	21.27	0.91	.	.
chr10	73341962	73342129	168	32	12	75.86	11.54	0.85	.	.
chr10	73555129	73555287	159	56	14	52.85	10.21	1.47	.	.
chr10	85609352	85609539	188	118	14	107.74	20.11	0.65	.	.
chr10	102027577	102027717	141	89	15	139.52	37.52	0	.	281:NM_018294:CWF19L1|
chr10	103124592	103124695	104	80	24	141.5	14.31	0	.	.
chr11	2306298	2306439	142	66	12	70.13	13.02	0.92	.	.
chr11	5311878	5312599	722	379	217	2485.12	122.27	0	.	.
chr11	10773231	10773367	137	43	11	80.96	22.29	1.08	-420:NM_014633:CTR9|	.
chr11	43598639	43598790	152	71	16	106.97	20.91	0.62	.	.
chr11	47747635	47747752	118	70	11	86.11	18.34	0.99	.	.
chr11	66057120	66057297	178	122	13	72.58	14.48	1.09	2253:NM_153266:TMEM151A|	660:NM_020470:YIF1A|
chr11	77516946	77517101	156	78	12	89.81	21.16	0.77	.	.
chr11	101073559	101073677	119	61	12	93.06	25.79	0.91	.	.
chr11	112021013	112021234	222	115	24	157.94	16.89	0	.	.
chr11	118797135	118797270	136	58	13	81.54	15.28	1.1	.	.
chr12	8868954	8869095	142	58	11	80.59	26.2	1.06	.	.
chr12	53135306	53135428	123	41	11	78.22	24.28	0.93	.	.
chr12	56203742	56203871	130	79	16	134.36	26.82	0	8064:NM_014182:ORMDL2|	-7668:NM_033082:SARNP|
chr12	57042458	57042561	104	63	13	118.99	32.75	0	.	2710:NM_001686:ATP5B|
chr12	85985227	85985323	97	46	12	123.66	37.52	0	.	.
chr12	98812996	98813098	103	39	11	107.33	28.92	0.64	.	.
chr12	105549728	105549905	178	103	15	122.99	28.57	0	.	.
chr12	108608436	108608645	210	44	13	78.91	12.84	0.95	.	.
chr12	117295952	117296132	181	102	14	111.08	28.66	0.7	.	.
chr14	33091150	33091253	104	59	11	99.11	24.69	1.07	.	.
chr14	90961717	90961859	143	63	12	105.15	39.07	0.61	.	.
chr14	95057304	95057394	91	29	13	52.85	10.01	1.47	-9573:NM_000624:SERPINA5|	.
chr15	64162762	64163061	300	169	31	237.77	27.51	0	.	.
chr16	11211397	11211556	160	70	13	93.99	22.6	0.95	.	.
chr16	12058873	12059000	128	44	11	89.81	21.49	0.77	91:NM_001192:TNFRSF17|	.
chr16	21144723	21144826	104	24	11	61.65	11.13	1.46	.	.
chr16	29805602	29805714	113	85	11	80.31	20.73	1.02	-3568:NM_001256269,NM_001256270,NM_007317:KIF22|	.
chr16	33905884	33906025	142	43	11	55.65	11.37	1.61	.	.
chr16	83050094	83050214	121	29	11	81.85	17.19	1.13	.	.
chr17	5144346	5144393	48	24	12	220.54	52.09	0	.	6311:NM_207103:SCIMP|
chr17	5144554	5144702	149	82	11	91.74	26.05	0.86	.	6620:NM_207103:SCIMP|
chr17	28456193	28456317	125	95	11	87.68	16.73	1.06	.	.
chr17	35198187	35198353	167	74	13	92.02	26.02	0.88	.	.
chr17	37005338	37005503	166	61	13	78.91	15.22	0.95	.	7862:NM_001080465:C17orf98|-4549:NM_000978:RPL23|
chr17	50624202	50624445	244	181	14	80.58	16.38	1.06	.	.
chr17	56081214	56081363	150	90	12	63.35	11.96	1.21	.	-3343:NM_001078166,NM_006924:SRSF1|
chr17	56197642	56197819	178	93	13	78.45	13.49	0.94	.	.
chr17	72667558	72667729	172	97	14	111.01	27.2	0.69	-302:NM_001006638,NM_001163989,NM_001163990,NM_175738:RAB37|	.
chr17	77290668	77290823	156	88	13	101.06	25.04	1.1	.	.
chr18	2767358	2767467	110	85	13	78.91	11.77	0.95	.	.
chr18	21027513	21027636	124	76	11	55.2	11.46	1.72	5274:NM_003831:RIOK3|	9712:NM_032933:TMEM241|
chr19	2740205	2740339	135	43	13	96.09	16.98	0.99	.	266:NM_144564,NM_213568:SLC39A3|
chr19	2865928	2866018	91	26	11	80.31	18.78	1.02	1405:NM_024967:ZNF556|	.
chr19	10172764	10172860	97	31	11	107.69	31.35	0.64	.	.
chr19	10806567	10806701	135	86	11	87.25	21.49	1.03	5545:NM_031209:QTRT1|	.
chr19	13552812	13553009	198	58	15	111.98	15.28	0.72	.	.
chr19	51163523	51163627	105	72	12	106.58	26.2	0.62	.	.
chr19	51899706	51899815	110	27	12	108.45	28.44	0.65	.	8606:NM_001161748,NM_030657:LIM2|5988:NM_001193623:LOC147646|
chr19	55728405	55728551	147	71	19	167.56	29.48	0	.	7678:NM_001161440,NM_002842:PTPRH|
chr19	58313749	58313906	158	33	17	145.97	28.98	0	.	.
chr2	2501326	2501472	147	85	11	92.39	21.71	0.88	.	.
chr2	19649935	19650067	133	91	11	97.24	21.71	1.02	.	.
chr2	33805048	33805185	138	45	13	101.03	25.3	1.09	.	.
chr2	41050089	41050214	126	32	14	111.89	26	0.71	.	.
chr2	48796345	48796483	139	69	11	75.33	20.06	0.84	.	.
chr2	48796571	48796702	132	48	11	84.05	19.65	1.25	.	.
chr2	49006652	49006786	135	35	11	80.31	24.76	1.02	.	.
chr2	55339054	55339165	112	45	11	61.65	11.98	1.46	.	.
chr2	62765971	62766119	149	71	12	89.85	23.6	0.77	.	.
chr2	65579204	65579296	93	48	12	110.42	21.83	0.67	.	.
chr2	67451938	67452074	137	57	12	90.53	25.13	0.81	.	.
chr2	144672490	144672592	103	80	12	103.96	21.4	0.6	.	.
chr2	176214740	176214909	170	90	12	85.41	14.11	0.96	.	.
chr2	176684937	176685148	212	151	15	86.13	16.18	1	.	.
chr20	29828167	29828268	102	31	15	60.74	11.74	1.61	.	.
chr20	29833148	29833299	152	89	20	80.83	14.95	1.07	.	.
chr20	34973025	34973174	150	74	11	78.02	16.38	0.92	.	.
chr20	41087420	41087551	132	70	11	61.65	10.09	1.46	.	.
chr20	44991894	44992129	236	47	15	66.9	10.82	1.07	.	-934:NM_015945,NM_173073,NM_173179:SLC35C2|
chr20	58342812	58342917	106	73	14	50.36	10.53	1.69	.	.
chr20	62958875	62958944	70	33	12	143.55	47.75	0	.	.
chr21	10086992	10087071	80	24	12	90.53	16.51	0.79	.	.
chr21	10780850	10781320	471	181	91	191.42	10.35	0	.	.
chr21	10781597	10781888	292	72	44	111.33	10.66	0.7	.	.
chr21	10820644	10820802	159	68	16	55.99	10.69	1.58	.	.
chr21	35433969	35434067	99	66	11	93.59	20.98	0.94	.	.
chr21	40721397	40721547	151	78	11	75.74	18.84	0.85	.	501:NM_004965:HMGN1|
chr3	9594015	9594183	169	102	12	88.96	18.79	0.75	.	-1302:NM_198560:LHFPL4|
chr3	15110410	15110532	123	80	11	73.03	19.31	1.12	.	3717:NM_022497:MRPS25|
chr3	15857688	15857840	153	67	13	89.89	17.19	0.78	.	.
chr3	18516350	18516461	112	28	13	97.61	19.31	1.04	.	.
chr3	20334686	20334787	102	32	13	123.48	24.32	0	.	.
chr3	20464141	20464268	128	68	11	85.52	16.38	0.96	.	.
chr3	22765046	22765174	129	72	11	75.99	18.88	0.85	.	.
chr3	37179355	37179507	153	66	13	77.86	13.76	0.92	.	.
chr3	38398191	38398470	280	209	26	141.35	17.12	0	-9940:NM_005108:XYLB|	.
chr3	39641045	39641181	137	68	12	85.83	20.06	0.98	.	.
chr3	43838051	43838241	191	84	17	107.29	20.38	0.63	.	.
chr3	128734050	128734200	151	61	13	106.59	30.57	0.62	.	.
chr3	187387531	187387687	157	69	12	90.53	18	0.79	.	-513:NM_001048:SST|
chr4	16942427	16942548	122	87	12	90.53	16.78	0.79	.	.
chr4	49114113	49114357	245	149	42	91.16	14.16	0.83	.	.
chr4	63348398	63348547	150	65	11	91.42	26.05	0.84	.	.
chr4	90013787	90013966	180	122	12	72.13	14.33	1.07	.	.
chr4	99533935	99534152	218	132	23	132.43	13.99	0	.	.
chr4	114745360	114745505	146	73	11	92.72	26.05	0.9	.	.
chr4	123125633	123125737	105	51	12	83.16	14.59	1.18	.	.
chr4	129691953	129692053	101	46	12	90.53	23.16	0.79	.	.
chr4	129692062	129692229	168	86	13	63.92	13.48	1.14	.	.
chr4	135285183	135285325	143	65	12	90.53	26.23	0.79	.	.
chr4	166395801	166395913	113	41	11	93.97	20.69	0.94	.	.
chr4	173415022	173415155	134	69	11	96.71	25.95	1.01	.	.
chr4	183604156	183604277	122	55	13	126	39.07	0	.	.
chr4	187615264	187615429	166	87	15	60.1	10.79	1.53	.	.
chr5	1247105	1247251	147	85	13	78.91	13.48	0.96	.	.
chr5	19122156	19122284	129	42	12	79.38	19.01	0.98	.	.
chr5	19893738	19893901	164	85	12	87.25	24.69	1.03	.	.
chr5	35873182	35873268	87	32	12	90.53	15.96	0.79	.	.
chr5	42907147	42907258	112	29	12	70.13	13.69	0.92	.	.
chr5	78838334	78838461	128	57	12	90.53	23.48	0.79	.	.
chr5	82283114	82283242	129	33	12	102.14	18.49	1.14	.	.
chr5	96979018	96979189	172	87	12	95.49	30.39	0.97	.	.
chr5	130413821	130413936	116	82	11	78.28	15.28	0.93	.	.
chr5	139780313	139780495	183	152	15	96.34	19.37	0.99	1086:NM_020690NM_001197030,NM_017747,NM_017978,NM_024668:ANKHD1-EIF4EBP3ANKHD1|	.
chr5	149507482	149507609	128	80	12	92.63	21.4	0.89	.	.
chr5	172145919	172146093	175	117	12	65.13	12.07	1.25	.	.
chr5	174098577	174098697	121	70	12	99.14	26.2	1.08	.	.
chr6	339643	339807	165	97	15	86.25	14.72	1	.	.
chr6	26215728	26215919	192	88	15	64.86	11.89	1.19	9655:NM_003532:HIST1H3E|1420:NM_021052:HIST1H2AE|	-952:NM_003518:HIST1H2BG|
chr6	33480560	33480703	144	85	13	80.56	18.16	1.05	.	.
chr6	36199960	36200049	90	40	11	91.71	20.96	0.86	.	.
chr6	37471332	37471475	144	91	13	78.91	17.61	0.95	.	3776:NM_138493:CCDC167|
chr6	38944243	38944442	200	46	22	110.78	14.23	0.68	.	.
chr6	40987638	40987756	119	27	11	79.77	13.49	1	.	.
chr6	43023483	43023619	137	65	11	57.96	13.49	1.76	3889:NM_138343,NM_201521,NM_201522,NM_201523:KLC4|	1937:NM_001168370,NM_014780:CUL7|-3622:NM_015950:MRPL2|
chr6	47227648	47227794	147	87	11	61.65	11.23	1.47	.	.
chr6	49082920	49083027	108	33	11	61.65	11.55	1.47	.	.
chr6	49435653	49435778	126	56	12	62.29	13.1	1.44	-4557:NM_018132:CENPQ|	4738:NM_000255:MUT|
chr6	50513516	50513842	327	232	27	146.43	21.72	0	.	.
chr6	57034776	57034906	131	81	12	70.13	14.02	0.92	2328:NM_004282:BAG2|	.
chr6	84559590	84559726	137	84	11	73.19	18.88	1.13	9780:NM_016230:CYB5R4|3395:NM_001009994:RIPPLY2|	.
chr6	90605137	90605297	161	87	13	96.44	27.17	1	-949:NM_032602:GJA10|	.
chr6	90611205	90611336	132	88	12	56.37	10.59	1.63	-7017:NM_032602:GJA10|	.
chr6	135462571	135462749	179	26	12	70.13	13.68	0.92	.	.
chr6	142410064	142410285	222	165	14	90.03	20.84	0.78	.	350:NM_002511:NMBR|
chr7	30711536	30711787	252	178	20	154.39	25.01	0	.	.
chr7	64126544	64126681	138	67	11	70.44	12.66	0.94	-33:NM_001013746,NM_016220:ZNF107|	.
chr7	72882751	72882897	147	58	13	101.49	20.38	1.14	.	.
chr7	100701825	100701994	170	62	13	80.91	16.18	1.08	.	.
chr7	105750346	105750463	118	33	13	89.23	19.65	0.76	.	-2593:NM_006754,NM_182715:SYPL1|
chr7	108247705	108247875	171	77	13	64.78	11.96	1.18	.	.
chr7	120055328	120055450	123	59	11	80.31	16.92	1.02	.	.
chr7	120750945	120751200	256	66	27	139.21	14.11	0	.	.
chr7	128687683	128687824	142	80	12	70.13	13.02	0.92	.	-7402:NM_001191028,NM_012470:TNPO3|
chr7	128934494	128934639	146	62	11	70.8	19.11	0.99	.	.
chr7	130495379	130495587	209	29	14	71.77	11.98	1.05	.	.
chr7	132001929	132002385	457	90	50	366.81	22.29	0	.	.
chr7	132750586	132750723	138	104	11	68.4	15.08	1.15	.	.
chr7	138085294	138085386	93	65	12	102.75	29.67	1.15	.	.
chr7	138085441	138085593	153	79	12	56.37	10.52	1.63	.	.
chr7	147700410	147700564	155	31	17	135.03	24.45	0	.	.
chr8	37855469	37855620	152	64	11	73.87	21.26	1.16	.	.
chr8	53624207	53624318	112	64	11	77.39	12.07	0.91	.	-2707:NM_001083617,NM_014781:RB1CC1|
chr8	80795754	80795871	118	87	12	110.82	27.91	0.68	.	.
chr8	96224042	96224186	145	76	11	61.65	11.08	1.46	.	.
chr8	106891294	106891455	162	70	17	116.83	29.71	0	.	.
chr8	120350535	120350678	144	95	11	93.39	21.71	0.93	.	.
chr8	125691717	125691830	114	50	11	80.31	15.68	1.02	.	.
chr8	125691900	125692133	234	70	15	77.21	14.79	0.9	.	.
chr9	71335971	71336255	285	216	25	160	14.33	0	.	.
chr9	71879957	71880100	144	51	12	104.79	30.39	0.6	.	.
chr9	94547018	94547253	236	84	20	92.25	12.62	0.88	.	.
chr9	101715656	101715844	189	96	13	72.27	12.74	1.07	-9518:NM_001855:COL15A1|	.
chr9	114345859	114345945	87	20	11	111.81	22.93	0.71	.	.
chr9	116310179	116310329	151	66	12	80.89	20.06	1.07	.	.
chr9	127799707	127799787	81	33	11	101.17	22.93	1.12	.	.
chr9	128368105	128368214	110	36	11	61.65	15.13	1.46	.	.
chr9	132552715	132553119	405	209	44	163.59	11.14	0	.	.
chr9	134674677	134674817	141	86	13	91.05	16.18	0.82	.	.
chr9	134989573	134989704	132	33	15	111.98	15.28	0.72	.	.
chr9	138276599	138276819	221	189	26	223.01	36.39	0	.	.
chr9	138276995	138277204	210	180	17	116.83	19.26	0	.	.
chr9	138809239	138809348	110	30	11	83.49	18.88	1.2	.	.
chr9	140779125	140779271	147	77	13	63.92	11.8	1.14	-6884:NM_000718,NM_001243812:CACNA1B|	.
chrX	48829583	48829716	134	67	12	83.87	18.88	1.23	.	1466:NM_004979:KCND1|
chrY	13837603	13837715	113	68	11	78.48	14.95	0.95	.	.

Common (sgRNA #6 and #17)
Target site

Supplementary Table S3. Enrichment of NGS reads corresponding to the *HBG1* and *HBG2* genes

Sample name	# Reads Total	# Reads HBG1 and HBG2 (TSSs ± 0.5 kb)	Normalized to total #reads (column C / column B)	Fold enrichment to input (enChIP / input)
dCas9_(-) (input)	36,541,063	30	8.21E-07	-
dCas9_(-) (enChIP)	34,309,199	24	7.00E-07	0.85
dCas9_NaB (input)	45,440,321	42	9.24E-07	-
dCas9_NaB (enChIP)	47,223,361	32	6.78E-07	0.73
dCas9+sgRNA#6_(-) (input)	36,617,514	25	6.83E-07	-
dCas9+sgRNA#6_(-) (enChIP)	45,609,281	34	7.45E-07	1.09
dCas9+sgRNA#6_NaB (input)	44,561,486	35	7.85E-07	-
dCas9+sgRNA#6_NaB (enChIP)	33,069,913	59	1.78E-06	2.27
dCas9+sgRNA#17_(-) (input)	48,786,495	38	7.79E-07	-
dCas9+sgRNA#17_(-) (enChIP)	39,473,425	34	8.61E-07	1.11
dCas9+sgRNA#17_NaB (input)	40,252,370	24	5.96E-07	-
dCas9+sgRNA#17_NaB (enChIP)	24,118,085	57	2.36E-06	3.96

Supplementary Table S4. NGS reads corresponding to the *IFNG* and *ACTA1* genes

Sample name	# Reads Total	# Reads IFNG (TSS ± 0.5 kb)	Normalized to total #reads (column C / column B)	Fold enrichment to input (enChIP / input)
dCas9_(-) (input)	36,541,063	13	3.56E-07	-
dCas9_(-) (enChIP)	34,309,199	15	4.37E-07	1.23
dCas9_NaB (input)	45,440,321	17	3.74E-07	-
dCas9_NaB (enChIP)	47,223,361	20	4.24E-07	1.13
dCas9+sgRNA#6_(-) (input)	36,617,514	9	2.46E-07	-
dCas9+sgRNA#6_(-) (enChIP)	45,609,281	15	3.29E-07	1.34
dCas9+sgRNA#6_NaB (input)	44,561,486	18	4.04E-07	-
dCas9+sgRNA#6_NaB (enChIP)	33,069,913	17	5.14E-07	1.27
dCas9+sgRNA#17_(-) (input)	48,786,495	19	3.89E-07	-
dCas9+sgRNA#17_(-) (enChIP)	39,473,425	19	4.81E-07	1.24
dCas9+sgRNA#17_NaB (input)	40,252,370	13	3.23E-07	-
dCas9+sgRNA#17_NaB (enChIP)	24,118,085	11	4.56E-07	1.41

Sample name	# Reads Total	# Reads ACTA1 (TSS ± 0.5 kb)	Normalized to total #reads (column C / column B)	Fold enrichment to input (enChIP / input)
dCas9_(-) (input)	36,541,063	10	2.74E-07	-
dCas9_(-) (enChIP)	34,309,199	14	4.08E-07	1.49
dCas9_NaB (input)	45,440,321	14	3.08E-07	-
dCas9_NaB (enChIP)	47,223,361	13	2.75E-07	0.89
dCas9+sgRNA#6_(-) (input)	36,617,514	11	3.00E-07	-
dCas9+sgRNA#6_(-) (enChIP)	45,609,281	17	3.73E-07	1.24
dCas9+sgRNA#6_NaB (input)	44,561,486	9	2.02E-07	-
dCas9+sgRNA#6_NaB (enChIP)	33,069,913	6	1.81E-07	0.90
dCas9+sgRNA#17_(-) (input)	48,786,495	16	3.28E-07	-
dCas9+sgRNA#17_(-) (enChIP)	39,473,425	13	3.29E-07	1.00
dCas9+sgRNA#17_NaB (input)	40,252,370	10	2.48E-07	-
dCas9+sgRNA#17_NaB (enChIP)	24,118,085	5	2.07E-07	0.83

Supplementary Table S5. Primers used in this study

Number	Name	Sequence (5' → 3')	Experiments
27420	hHS5-TAL-Target-F	ccagtttctccagtttccctttt	enChIP assays in Figure 2C (5'HS5)
27421	hHS5-TAL-Target-R	ttttcaaaatgcaaggtgatgtc	enChIP assays in Figure 2C (5'HS5)
27222	hSox2-prom-F	attggtcgctagaaacccatttatt	enChIP assays in Figure 2C (Sox2)
27223	hSox2-prom-R	ctgccttgacaactcctgatacttt	enChIP assays in Figure 2C (Sox2)
27915	h5'HS5-3D-3'-ISceI-F	(Biotin)ccggTAGGGATAACAGGGTAATttgagaaggtagggttgcatgag	1 st PCR in proximity ligation assay in Figure 5A-C (Upper cases: I-Sce I site)
27880	hZNF670-3D-F2	gagtcttggactcgggctca	1 st PCR in proximity ligation assay in Figure 5A
27863	hZNF670-3D-F	atctttggggtgaagttcccttt	2nd PCR in proximity ligation assay in Figure 5A and PCR in Supplementary Figure S3A
27864	hZNF670-3D-R	ccaagagatctggctgctaaaca	2nd PCR in proximity ligation assay in Figure 5A and PCR in Supplementary Figure S3A
27865	hMIR422A-3D-F	ggctatcctagcttggctcagaa	1 st PCR in proximity ligation assay in Figure 5B
28038	hMIR422A-3D-F4	ctggacagctcccatgctatta	2nd PCR in proximity ligation assay in Figure 5B and PCR in Supplementary Figure S3B
28039	hMIR422A-3D-R4	tactctggagctctgggtctgat	2nd PCR in proximity ligation assay in Figure 5B and PCR in Supplementary Figure S3B
27868	hYIF1A-3D-R	tatcagtgacagagctgccaagc	1 st PCR in proximity ligation assay in Figure 5C
28040	hYIF1A-3D-F3	cgggaggacattttcagcac	2nd PCR in proximity ligation assay in Figure 5C and PCR in Supplementary Figure S3C
28041	hYIF1A-3D-R3	aagccccaggatgctgactc	2nd PCR in proximity ligation assay in Figure 5C and PCR in Supplementary Figure S3C
27771	hZNF670-ZNF695-Ex1_2-F	agcttctgttgctctgggacct	RT-PCR in Figure 6 (ZNF670-ZNF695)
27772	hZNF670-ZNF695-Ex1_2-R	cacatccctgaatgccaatagtc	RT-PCR in Figure 6 (ZNF670-ZNF695)
27779	hZNF670-Ex4-F2	gttccccaggaggatgtatgttc	RT-PCR in Figure 6 (ZNF670)
27780	hZNF670-Ex4-R2	acgagatgcagttaccacagagc	RT-PCR in Figure 6 (ZNF670)
27773	hMIR422A-F	gacttagggtcagaaggcctgag	RT-PCR in Figure 6 (MIR422A)
27774	hMIR422A-R	aagcttggctcagggacagag	RT-PCR in Figure 6 (MIR422A)
27782	hTMEM151A-Ex2-F	cttagatgcccgagtgattgttg	RT-PCR in Figure 6 (TMEM151A)
27783	hTMEM151A-Ex2-R	cttaggcttccacttcctccaaa	RT-PCR in Figure 6 (TMEM151A)
27777	hYIF1A-Ex1_2-F	agcagctgcctcattagtattcg	RT-PCR in Figure 6 (YIF1A)
27778	hYIF1A-Ex1_2-R	gaataaccaccgcttgtgtcatc	RT-PCR in Figure 6 (YIF1A)
27784	hCNIH2-Ex1_4-F	gcctccctcatcttctttgtcat	RT-PCR in Figure 6 (CNIH2)
27785	hCNIH2-Ex1_4-R	agtattctgggaccaccagcttc	RT-PCR in Figure 6 (CNIH2)

Supplementary Table S6. Information on enChIP-Seq analysis

140318_SN7001075L_0319_AC42GNACXX (Hiseq2500, 36 bp single end) reference: hg19

Sample Name	Lane	Sample ID	Index	Description	Yield (Mbases)	% PF	# Reads	% of raw clusters per lane	% of >= Q30 Bases (PF)	Mean Quality Score (PF) Max =40	% Align (PF) =% uniquely mapped reads	% Mismatch Rate (PF)	mode of sequencing	Library Prep Kit	PCR cycles	Redundant rate
dCas9+sgRNA#6_(-) (enChIP)	7	140318_Hiseq3A_l7_001_Dr_Fujita_ChIP	ATCACG	1_HS5_no6_none	1,557	94.81	45,609,281	25.78	98.03	38.26	80.15	0.20	Single-End	TruSeq ChIP Sample Prep Kit (IP-202-1012, Illumina	18	6%
dCas9+sgRNA#17_(-) (enChIP)	7	140318_Hiseq3A_l7_010_Dr_Fujita_ChIP	TAGCTT	2_HS5_no17_none	1,349	94.94	39,473,425	22.31	98.10	38.29	79.61	0.20	Single-End	TruSeq ChIP Sample Prep Kit (IP-202-1012, Illumina	18	6%
dCas9+sgRNA#17_NaB (input)	7	140318_Hiseq3A_l7_011_Dr_Fujita_ChIP	GGCTAC	8_HS5_no17_NaB_input	1,379	95.17	40,252,370	22.75	98.17	38.32	75.34	0.21	Single-End	TruSeq ChIP Sample Prep Kit (IP-202-1012, Illumina	18	4%
dCas9+sgRNA#6_NaB (enChIP)	7	140318_Hiseq3A_l7_020_Dr_Fujita_ChIP	GTGGCC	3_HS5_no6_NaB	1,141	95.82	33,069,913	18.69	98.26	38.33	79.41	0.20	Single-End	TruSeq ChIP Sample Prep Kit (IP-202-1012, Illumina	18	7%
dCas9+sgRNA#17_NaB (enChIP)	8	140318_Hiseq3A_l8_003_Dr_Fujita_ChIP	TTAGGC	4_HS5_no17_NaB	835	96.20	24,118,085	14.04	98.29	38.35	79.66	0.24	Single-End	TruSeq ChIP Sample Prep Kit (IP-202-1012, Illumina	18	6%
dCas9+sgRNA#6_NaB (input)	8	140318_Hiseq3A_l8_008_Dr_Fujita_ChIP	ACTTGA	7_HS5_no6_NaB_input	1,516	94.48	44,561,486	25.94	98.05	38.29	76.36	0.25	Single-End	TruSeq ChIP Sample Prep Kit (IP-202-1012, Illumina	18	4%
dCas9+sgRNA#6_(-) (input)	8	140318_Hiseq3A_l8_009_Dr_Fujita_ChIP	GATCAG	5_HS5_no6_none_input	1,249	94.76	36,617,514	21.32	98.02	38.27	74.73	0.26	Single-End	TruSeq ChIP Sample Prep Kit (IP-202-1012, Illumina	18	4%
dCas9+sgRNA#17_(-) (input)	8	140318_Hiseq3A_l8_025_Dr_Fujita_ChIP	ACTGAT	6_HS5_no17_none_input	1,644	93.62	48,786,495	28.40	97.88	38.23	74.64	0.26	Single-End	TruSeq ChIP Sample Prep Kit (IP-202-1012, Illumina	18	4%

141202_SN7001075L_0366_AC5Y10ACXX (Hiseq2500, 36 bp single end) reference: hg19

Sample Name	Lane	Sample ID	Index	Description	Yield (Mbases)	% PF	# Reads	% of raw clusters per lane	% of >= Q30 Bases (PF)	Mean Quality Score (PF) Max =40	% Align (PF)	% Mismatch Rate (PF)	mode of sequencing	Library Prep Kit	PCR cycles	Redundant rate
dCas9_NaB (enChIP)	1	141202_Hiseq3A_l1_001_Dr_Fujita_ChIP	ATCAC	en_ChIP_NaB	1,620	95.32	47,223,361	27.55	98.07	38.38	79.60	0.18	Single-End	TruSeq ChIP Sample Prep Kit (IP-202-1012, Illumina	18	2%
dCas9_(-) (enChIP)	1	141202_Hiseq3A_l1_008_Dr_Fujita_ChIP	ACTTG	en_ChIP_minus	1,182	95.70	34,309,199	20.01	98.10	38.39	78.81	0.18	Single-End	TruSeq ChIP Sample Prep Kit (IP-202-1012, Illumina	18	1%
dCas9_NaB (input)	1	141202_Hiseq3A_l1_010_Dr_Fujita_ChIP	TAGCT	1p_input_NaB	1,517	92.73	45,440,321	26.51	97.68	38.27	74.96	0.20	Single-End	TruSeq ChIP Sample Prep Kit (IP-202-1012, Illumina	18	2%
dCas9_(-) (input)	1	141202_Hiseq3A_l1_011_Dr_Fujita_ChIP	GGCTA	1p_input_minus	1,228	93.37	36,541,063	21.32	97.75	38.29	76.09	0.20	Single-End	TruSeq ChIP Sample Prep Kit (IP-202-1012, Illumina	18	1%

Figure S1

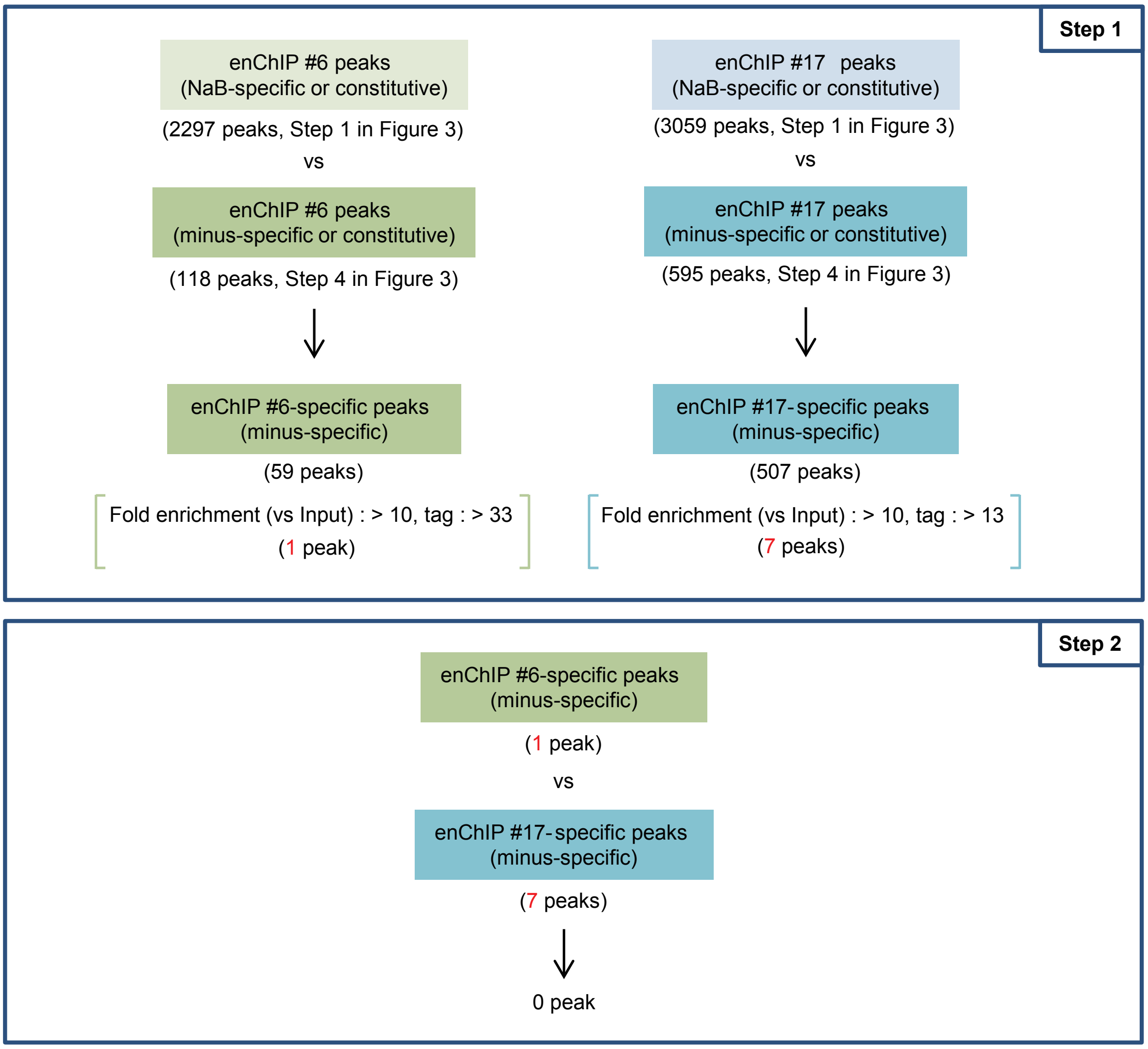

Step 1
enChIP #6 peaks
(NaB-specific or constitutive)
(2297 peaks, Step 1 in Figure 3)
vs
enChIP #6 peaks
(minus-specific or constitutive)
(118 peaks, Step 4 in Figure 3)
enChIP #6-specific peaks
(minus-specific)
(59 peaks)
Fold enrichment (vs Input) : > 10, tag : > 33
(1 peak)
enChIP #17 peaks
(NaB-specific or constitutive)
(3059 peaks, Step 1 in Figure 3)
vs
enChIP #17 peaks
(minus-specific or constitutive)
(595 peaks, Step 4 in Figure 3)
enChIP #17-specific peaks
(minus-specific)
(507 peaks)
Fold enrichment (vs Input) : > 10, tag : > 13
(7 peaks)
Step 2
enChIP #6-specific peaks
(minus-specific)
(1 peak)
vs
enChIP #17-specific peaks
(minus-specific)
(7 peaks)
0 peak

Figure S2

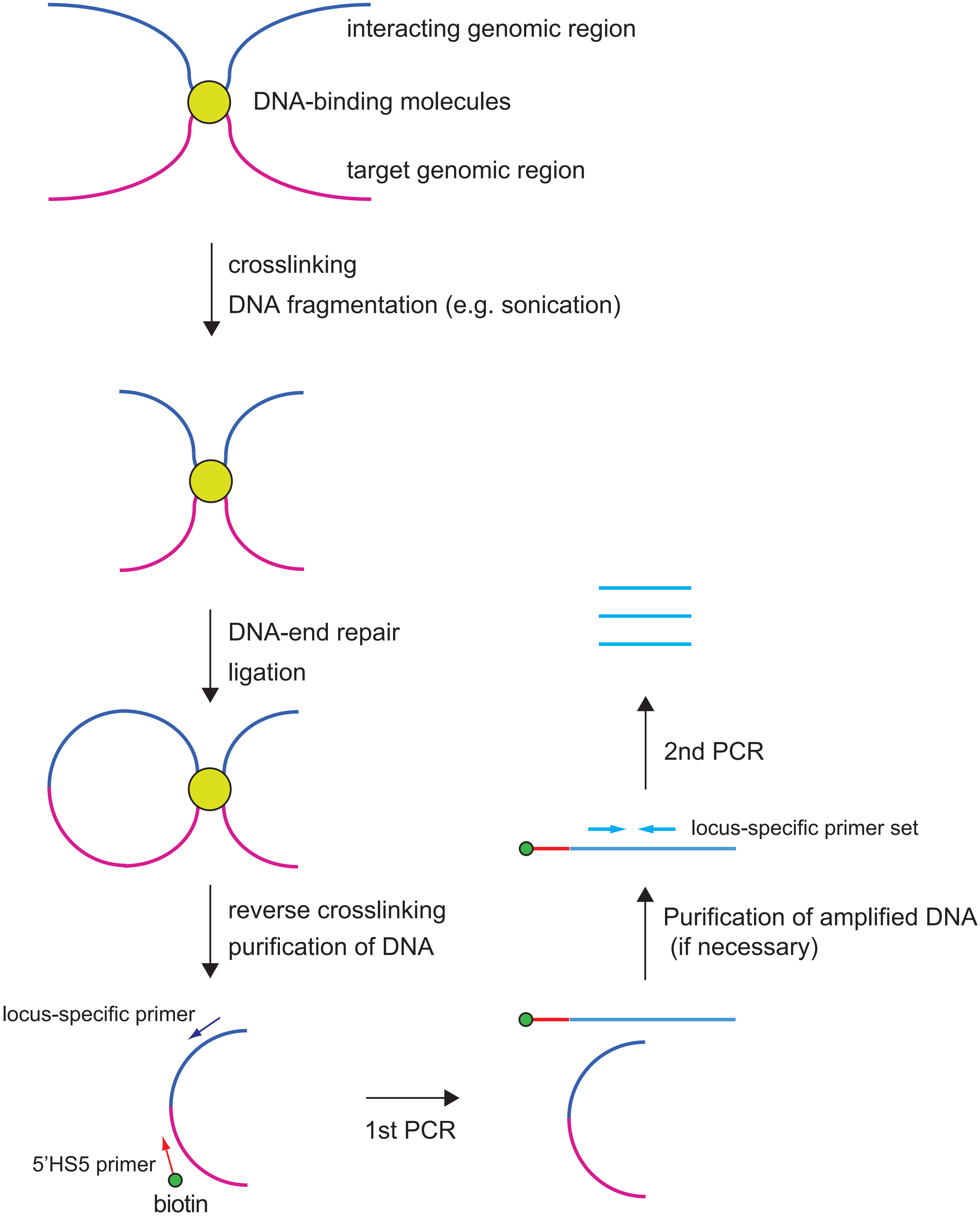
interacting genomic region
DNA-binding molecules
target genomic region
crosslinking
DNA fragmentation (e.g. sonication)
DNA-end repair
ligation
reverse crosslinking
purification of DNA
locus-specific primer
5'HS5 primer
biotin
1st PCR
Purification of amplified DNA
(if necessary)
locus-specific primer set
2nd PCR

Figure S3

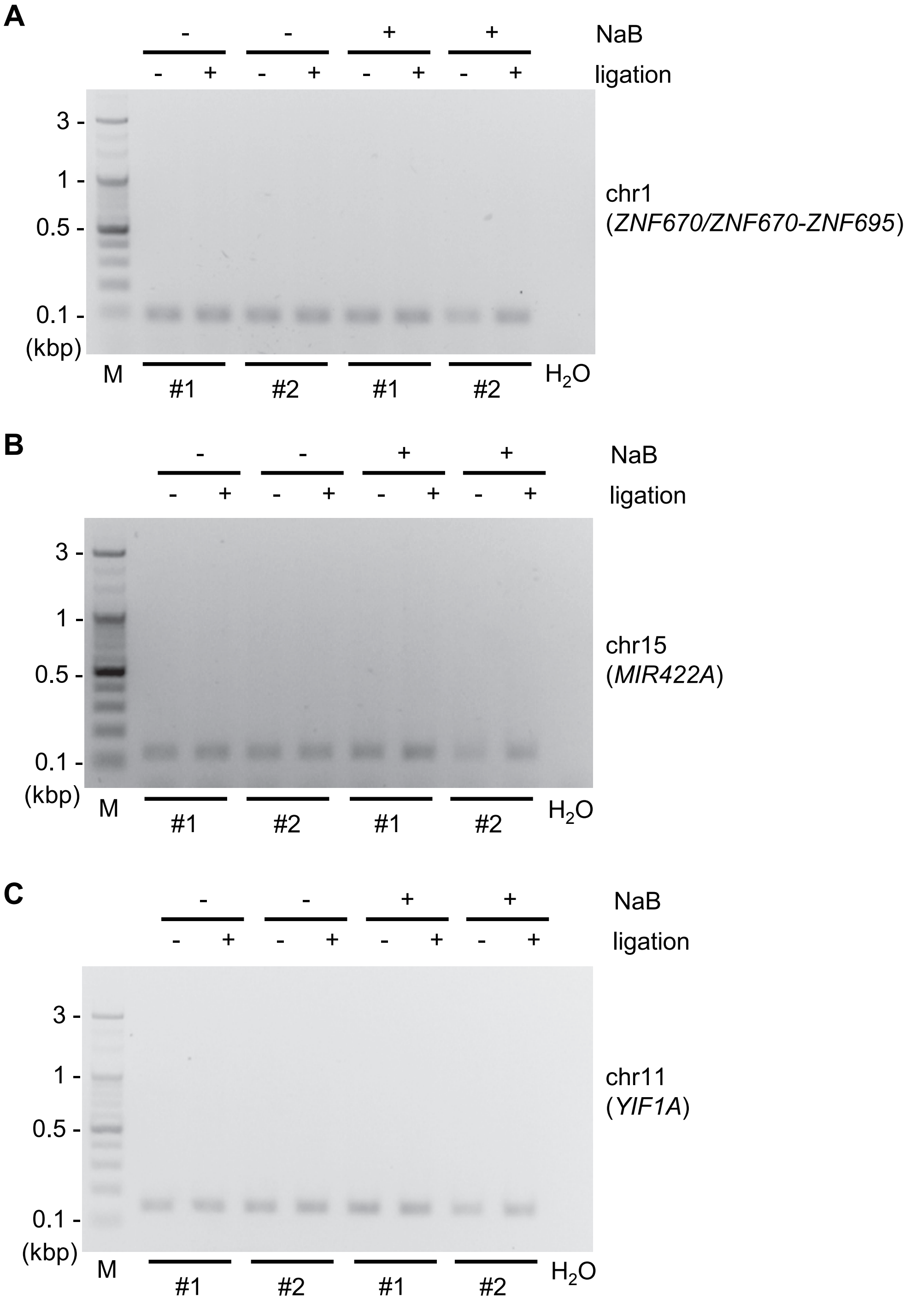
A
- - + +
NaB
- + - + - + - +
ligation
3 -
1 -
0.5 -
0.1 -
(kbp)
M
#1 #2 #1 #2
H_2O
chr1
(*ZNF670/ZNF670-ZNF695*)
B
- - + +
NaB
- + - + - + - +
ligation
3 -
1 -
0.5 -
0.1 -
(kbp)
M
#1 #2 #1 #2
H_2O
chr15
(*MIR422A*)
C
- - + +
NaB
- + - + - + - +
ligation
3 -
1 -
0.5 -
0.1 -
(kbp)
M
#1 #2 #1 #2
H_2O
chr11
(*YIF1A*)

Figure S4

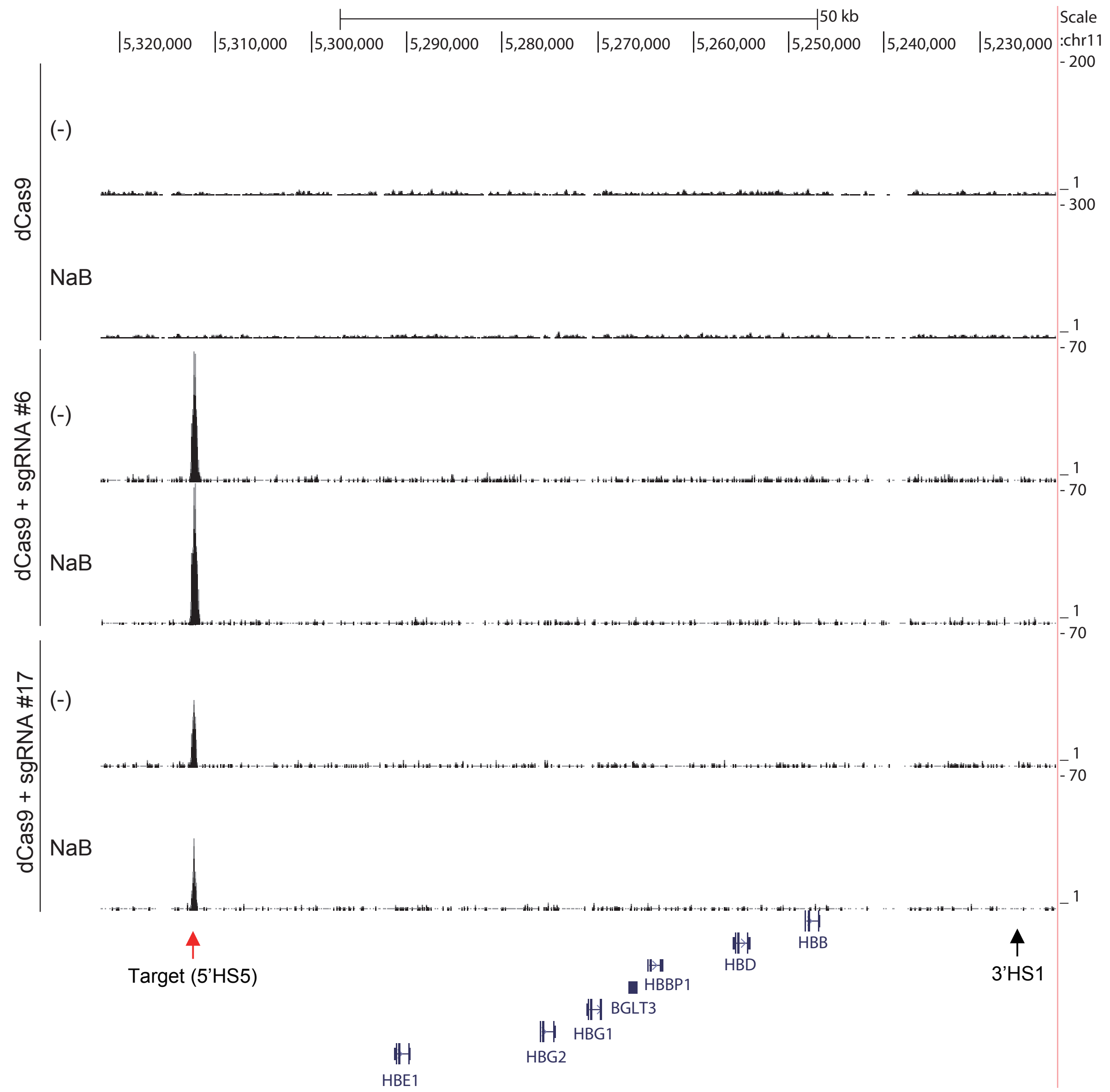

50 kb
Scale
chr11
5,320,000
5,310,000
5,300,000
5,290,000
5,280,000
5,270,000
5,260,000
5,250,000
5,240,000
5,230,000
dCas9
(-)
NaB
dCas9 + sgRNA #6
(-)
NaB
dCas9 + sgRNA #17
(-)
NaB
- 200
_ 1
- 300
_ 1
- 70
_ 1
- 70
_ 1
- 70
_ 1
- 70
_ 1
Target (5'HS5)
HBE1
HBG2
HBG1
BGLT3
HBBP1
HBD
HBB
3'HS1

Figure S5

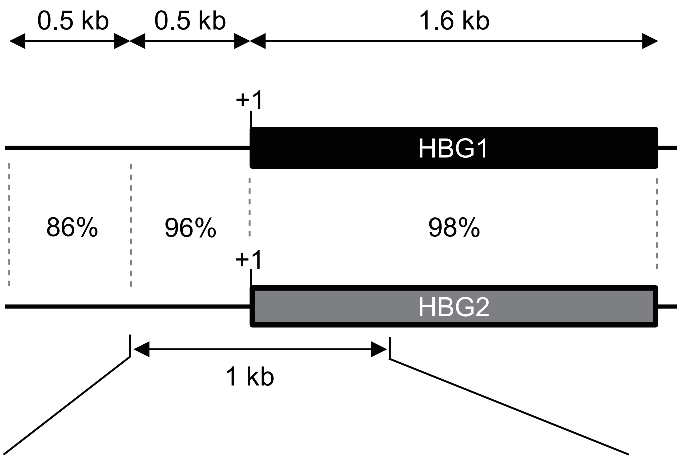


```
HBG1   1' ACTGCTAACTGAAAGAGACTAAGATTTGTCCCGTCAAAAATCCTGGACCTATGCCTAAAA
              ** *** ** **********  ****** ***************************
HBG2   1"     CTTACTAAATGAGACTAAGACGTGTCCCATCAAAAATCCTGGACCTATGCCTAAAA

      61' CACATTTCACAATCCCTGAACTTTTCAAAAATTGGTACATGCTTTAGCTTTAAACTACAG
          ********************************************* **************
      57" CACATTTCACAATCCCTGAACTTTTCAAAAATTGGTACATGCTTTAACTTTAAACTACAG

     121' GCCTCACTGGAGCTAGAGACAAGAAGGTAAAAAACGGCTGACAAAAGAAGTCCTGGTATC
          *************** ************ *******************************
     117" GCCTCACTGGAGCTACAGACAAGAAGGTGAAAAACGGCTGACAAAAGAAGTCCTGGTATC

     181' CTCTATGATGGGAGAAGGAAACTAGCTAAAGGGAAGAATAAATTAGAGAAAAACTGGAAT
           ****** ********* *********************************** ******
     177" TTCTATGGTGGGAGAAGAAAACTAGCTAAAGGGAAGAATAAATTAGAGAAAAATTGGAAT

     241' GACTGAATCGGAACAAGGCAAAGGCTATAAAAAAAATT----AGCAGTATCCTCTTGGGG
          **************************************    ******************
     237" GACTGAATCGGAACAAGGCAAAGGCTATAAAAAAAATTAAGCAGCAGTATCCTCTTGGGG

     297' GCCCCTTCCCCACACTATCTCAATGCAAATATCTGTCTGAAACGGTCCCTGGCTAAACTC
          ************************************************************
     297" GCCCCTTCCCCACACTATCTCAATGCAAATATCTGTCTGAAACGGTCCCTGGCTAAACTC

     357' CACCCATGGGTTGGCCAGCCTTGCCTTGACCAATAGCCTTGACAAGGCAAACTTGACCAA
          ************************************************************
     357" CACCCATGGGTTGGCCAGCCTTGCCTTGACCAATAGCCTTGACAAGGCAAACTTGACCAA

     417' TAGTCTTAGAGTATCCAGTGAGGCCAGGGGCCGGCGGCTGGCTAGGGATGAAGAATAAAA
          ************************************************************
     417" TAGTCTTAGAGTATCCAGTGAGGCCAGGGGCCGGCGGCTGGCTAGGGATGAAGAATAAAA

     477' GGAAGCACCCTTCAGCAGTTCCACACACTCGCTTCTGGAACGTCTGAGGTTATCAATAAG
          ************************************************************
     477" GGAAGCACCCTTCAGCAGTTCCACACACTCGCTTCTGGAACGTCTGAGGTTATCAATAAG

     537' CTCCTAGTCCAGACGCCATGGGTCATTTCACAGAGGAGGACAAGGCTACTATCACAAGCC
          ************************************************************
     537" CTCCTAGTCCAGACGCCATGGGTCATTTCACAGAGGAGGACAAGGCTACTATCACAAGCC

     597' TGTGGGGCAAGGTGAATGTGGAAGATGCTGGAGGAGAAACCCTGGGAAGGTAGGCTCTGG
          ************************************************************
     597" TGTGGGGCAAGGTGAATGTGGAAGATGCTGGAGGAGAAACCCTGGGAAGGTAGGCTCTGG

     657' TGACCAGGACAAGGGAGGGAAGGAAGGACCCTGTGCCTGGCAAAAGTCCAGGTCGCTTCT
          ************************************************************
     657" TGACCAGGACAAGGGAGGGAAGGAAGGACCCTGTGCCTGGCAAAAGTCCAGGTCGCTTCT

     717' CAGGATTTGTGGCACCTTCTGACTGTCAAACTGTTCTTGTCAATCTCACAGGCTCCTGGT
          ************************************************************
     717" CAGGATTTGTGGCACCTTCTGACTGTCAAACTGTTCTTGTCAATCTCACAGGCTCCTGGT

     777' TGTCTACCCATGGACCCAGAGGTTCTTTGACAGCTTTGGCAACCTGTCCTCTGCCTCTGC
          ************************************************************
     777" TGTCTACCCATGGACCCAGAGGTTCTTTGACAGCTTTGGCAACCTGTCCTCTGCCTCTGC

     837' CATCATGGGCAACCCCAAAGTCAAGGCACATGGCAAGAAGGTGCTGACTTCCTTGGGAGA
          ************************************************************
     837" CATCATGGGCAACCCCAAAGTCAAGGCACATGGCAAGAAGGTGCTGACTTCCTTGGGAGA

     897' TGCCACAAAGCACCTGGATGATCTCAAGGGCACCTTTGCCCAGCTGAGTGAACTGCACTG
          ***** ******************************************************
     897" TGCCATAAAGCACCTGGATGATCTCAAGGGCACCTTTGCCCAGCTGAGTGAACTGCACTG

     957' TGACAAGCTGCATGTGGATCCTGAGAACTTCAAGGTGAGTCCAG
          ********************************************
     957" TGACAAGCTGCATGTGGATCCTGAGAACTTCAAGGTGAGTCCAG
```

Figure S6

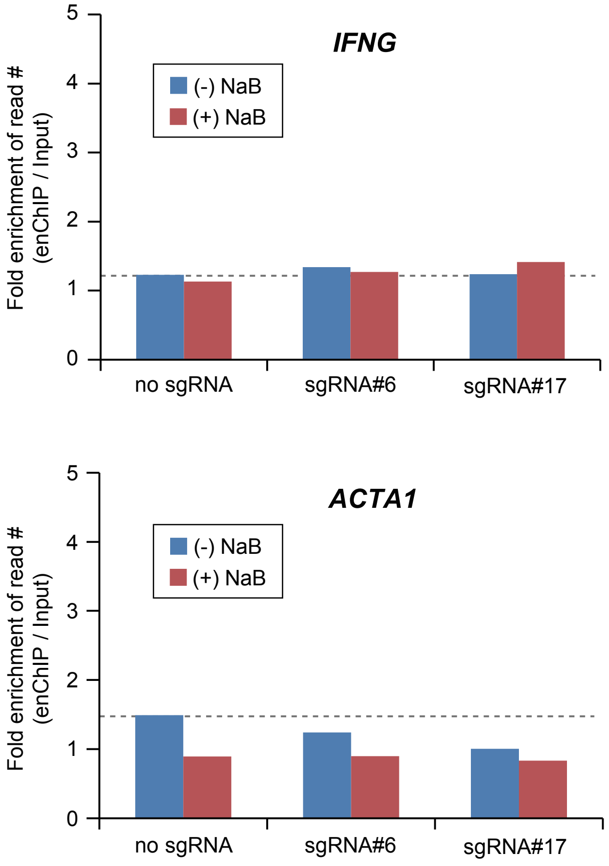
IFNG
(-) NaB
(+) NaB
Fold enrichment of read #
(enChIP / Input)
0
1
2
3
4
5
no sgRNA
sgRNA#6
sgRNA#17
ACTA1
(-) NaB
(+) NaB
Fold enrichment of read #
(enChIP / Input)
0
1
2
3
4
5
no sgRNA
sgRNA#6
sgRNA#17
