# Supplementary material for: Locus-specific ChIP combined with NGS analysis reveals genomic regulatory regions that physically interact with the Pax5 promoter in a chicken B cell line
Source: DNA Res. 2017 Jun 6;24(5):537–48. doi: 10.1093/dnares/dsx023 (PMC5737561; doi:10.1093/dnares/dsx023)
Supplement: Supplementary Data [file dsx023_supp_170329_data_for_reviewers.pdf]

Result of RNA-Seq

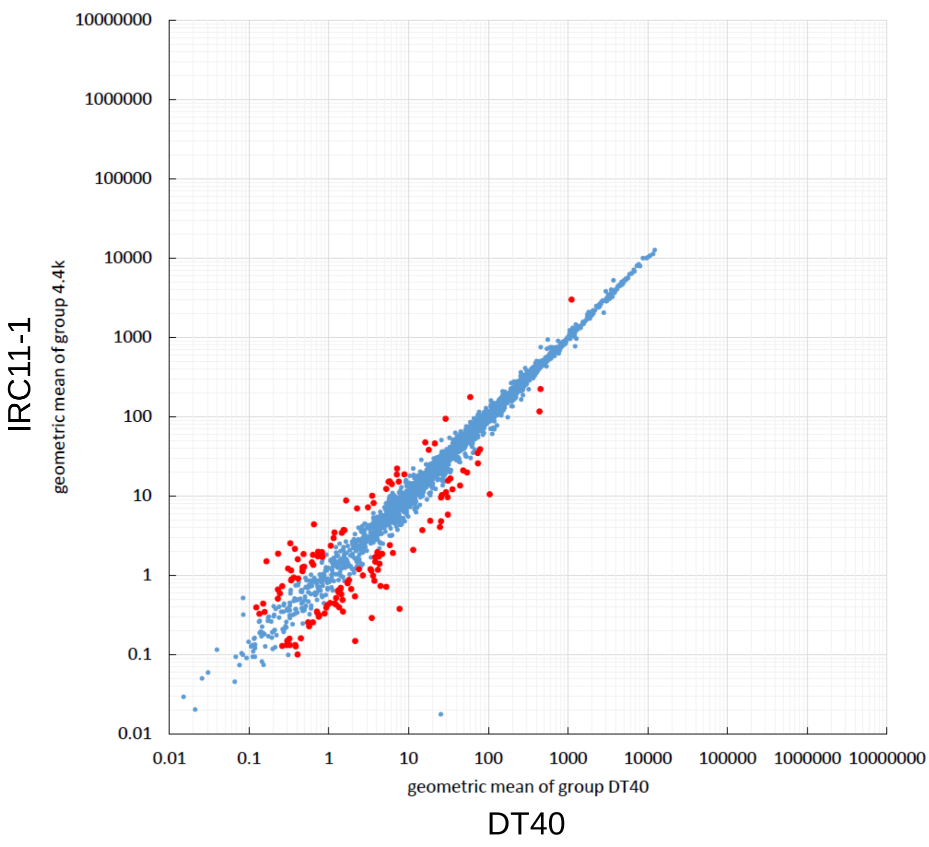

Data for reviewers (1)

	4.4k vs DT40
tracking_id	Fold Change
MIR6572	1773.314
MBOAT1	55.974
GPX2	10.717
BCL2A1	9.918
CD82	9.075
EMP1	8.117
COL1A2	6.727
SYPL1	5.718
MIXL1	5.529
PROM1	5.301
LOC417192	5.214
ZP1	5.203
CRP	4.670
CIDEA	3.812
COTL1	3.230
FAM65B	3.085
CD5	3.082
CD72	2.950
LGALS1	2.922
ANKRD1	2.912
GAS2L3	2.867
MAPK11	2.858
ROR1	2.838
CCDC28B	2.838
NPL	2.619
LOC420160	2.616
TLR3	2.581
CGNRH-R	2.416
NR3C1	2.310
MTSS1	2.280
CD247	2.232
UCP3	2.201
PLIN1	2.201
TXNRD1	2.153
GSTA3	2.130
MKRN3	2.071
PAX3	2.065

	4.4k vs DT40
tracking_id	Fold Change
ATP1B1	-2.002
MXRA8	-2.032
FUT4	-2.032
ENG	-2.032
GLCCI1	-2.039
RFTN2	-2.058
NOX1	-2.088
LOC395933	-2.103
SLC25A36	-2.110
ANKDD1A	-2.245
LOC395100	-2.265
CDH1	-2.272
GPM6B	-2.315
MCAM	-2.393
ARID5B	-2.409
GNOT1	-2.447
SSX2IP	-2.469
BMPR2	-2.470
SDC4	-2.488
ANXA2	-2.567
FGF12	-2.669
B3GNT5	-2.693
MMP2	-2.717
LAMP3	-2.744
CPS1	-2.782
IGF1R	-2.812
PRR5	-2.927
TACSTD2	-3.136
RSPH9	-3.201
ST3GAL5	-3.207
SALL4	-3.336
SYT1	-3.344
NEURL1	-3.430
SLC31A2	-3.628
ZAR1L	-3.645
MAT1A	-3.663
CACNA2D1	-3.804
BEND7	-3.836
RBM38	-4.041
NSG1	-4.310
SNTB1	-4.399
GHRH	-4.553
NPTN	-5.165
PTPRS	-5.322
AICDA	-5.339
KLHL14	-5.504
AP1S3	-6.092
FZD2	-6.096
POPDC3	-6.105
USP2	-6.122
SMIM18	-6.928
PMP22	-7.222
FGF18	-7.677
MT3	-9.954
IL10	-9.981
PPARG	-11.963
SCIN	-14.419
SMAD3	-17.605
SRC	-21.922
SOX9	-65.745
FAM213A	-252.950
BVES	-394.600
HAND2	-2261.502
MIR1804	-21350.537

PAX5	-1.193

Results of iChIP-Seq and *in vitro* enChIP-Seq

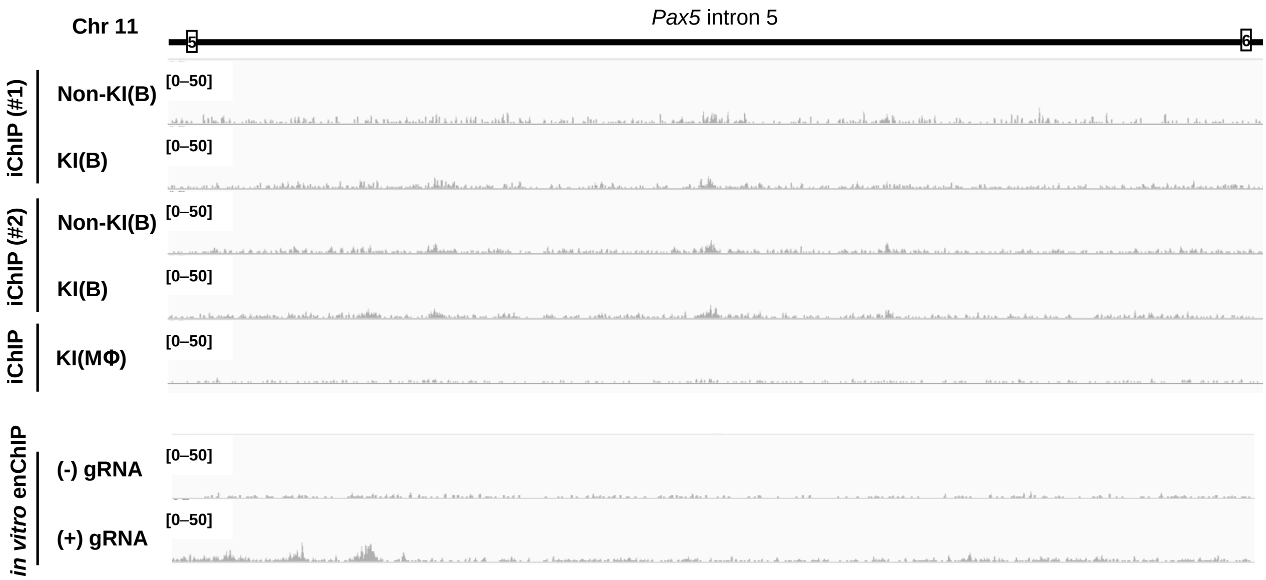

Data for reviewers (2)
