# Supplementary material for: Locus-specific ChIP combined with NGS analysis reveals genomic regulatory regions that physically interact with the Pax5 promoter in a chicken B cell line
Source: DNA Res. 2017 Jun 6;24(5):537–48. doi: 10.1093/dnares/dsx023 (PMC5737561; doi:10.1093/dnares/dsx023)
Supplement: Supplementary Data [file dsx023_supp_170401_supplementaryinfo.pdf]

Supplementary Information

Locus-specific ChIP combined with NGS analysis reveals genomic regulatory regions that physically interact with the *Pax5* promoter in a chicken B cell line

Toshitsugu Fujita, Fusako Kitaura, Miyuki Yuno, Yutaka Suzuki, Sumio Sugano, and Hodaka Fujii

Supplementary Table and Figure Legends

Supplementary Table S1. gRNA and primers used in this study

Supplementary Table S2. Information on iChIP-Seq and in vitro enChIP-Seq analyses

Supplementary Table S3. List of genomic regions detected by iChIP-Seq

Supplementary Figure S1. Genomic regions that interact with the *Pax5* promoter region. (**A**) Thirty four genomic regions were commonly detected in Data set #1 (Fold enrichment, >7) and #2 (Fold enrichment, >7). (**B**) Ninety (75.6%) genomic regions in Data set #1 (Fold enrichment, >7) were detected in Data set #2 (Fold enrichment, >2). (**B**) Sixty two (59.0%) genomic regions in Data set #2 (Fold enrichment, >7) were detected in Data set #1 (Fold enrichment, >2).

Supplementary Figure S2. Isolation of the *Pax5* promoter region by *in vitro* enChIP. (**A**) Positions of gRNA and the primer set. (**B**) Target sequence of Pax5 gRNA. The protospacer adjacent motif (PAM) is underlined. (**C**) DNA yields of *in vitro* enChIP. DNA isolated by *in vitro* enChIP was used for quantitative real-time PCR.

Supplementary Figure S3. Representative peak positions detected by iChIP-Seq (#1). Three peak positions in chromosome 11 (**A–C**) and one peak position in chromosome 21 (**D**) are shown. The peak positions corresponding to iChIP-Seq (#2) are shown in Figure 4.

Supplementary Figure S4. Representative peak positions not confirmed by *in vitro* enChIP-Seq. (**A–C**) Two peak positions in chromosome 21 that were detected by iChIP-Seq but not by *in vitro* enChIP-Seq.

Supplementary Figure S5. Prediction of potential off-target binding sites. Potential off-target binding sites for Pax5 gRNA were predicted using a CRISPR design tool (http://crispr.mit.edu/) and the chicken genome reference galGal4.

Supplementary Figure S6. Intra-chromosomal interaction with the *Pax5* promoter region. Intra-chromosomal interactions in Data set #1 (Fold enrichment, >2) and #2 (Fold enrichment, >2) are shown.

Supplementary Figure S7. Distribution of identified genomic regions in chromosome 21.

Supplementary Figure S8. The LexA BE insertion site and Pax5 gRNA-targeted site in the *Pax5* promoter. The genomic region upstream from nucleotide -1 is shown.

Supplementary Figure S9. Representative peak positions constitutively detected by iChIP-Seq in KI(B) and KI(MΦ). Two representative peak positions on chromosome 21 are shown.

Supplementary Figure S10. *in vitro* enChIP-Seq with DT40(MΦ). (**A**) Trans-differentiation of DT40 into a macrophage-like cell by stable expression of chicken C/EBPβ. (**B**) Silencing of *Pax5* and *AID* transcription in the macrophage-like cell DT40(MΦ). Expression levels of *Pax5* and *AID* mRNA were quantified by real-time RT-PCR and

normalized against *GAPDH* mRNA (mean ± s.e.m., n = 3); the mRNA levels in DT40 were defined as 1. (**C**) *M-CSFR* transcription in DT40(MΦ). *M-CSFR* transcription was confirmed by RT-PCR analysis. (**D**) Unsuccessful isolation of the *Pax5* promoter region from DT40(MΦ) by *in vitro* enChIP. The images of *in vitro* enChIP-Seq with DT40 in Figure 3C are also shown here for comparison with that of *in vitro* enChIP-Seq with DT40(MΦ).

Supplementary Figure S11. CRISPR-mediated locus deletion. (**A**) Schematic depiction of the loci in chromosome 11, which were identified as those interacting with the *Pax5* promoter region in a B cell–specific manner. One allele is shown. The interacting regions are shown with gray triangles. Numbers indicate target sites of sgRNAs. (**B**) Scheme of CRISPR-mediated locus deletion. (**C**) Target sequences of sgRNAs. (**D** and **E**) Scheme of CRISPR-mediated locus deletion in DT40. WT indicates intact DT40.

Supplementary Figure S12. Confirmation of CRISPR-mediated locus deletion by genotyping PCR. (**A**) Depiction of the genomic regions in each cell clone. Primer positions used in genotyping PCR are shown. (**B**) Results of genotyping PCR.

Supplementary Materials and Methods

Trans-differentiation of DT40 cells into macrophage-like cells.

For trans-differentiation into macrophage-like cells, DT40 cells (1×10^7) were co-transfected with 110 μg of linearized cC/EBPβ/pEF (Fujita et al. 2015) and 3 μg of puromycin-resistance gene by electroporation. The cells were selected in the presence of puromycin (0.35 μg/ml). The surviving cell colonies were individually picked and expanded. An established cell line was used as DT40(MΦ) in Supplementary Figure S8.
