# Supplementary material for: Locus-specific ChIP combined with NGS analysis reveals genomic regulatory regions that physically interact with the Pax5 promoter in a chicken B cell line
Source: DNA Res. 2017 Jun 6;24(5):537–48. doi: 10.1093/dnares/dsx023 (PMC5737561; doi:10.1093/dnares/dsx023)
Supplement: Supplementary Data [file dsx023_supplfigures.pdf]

A

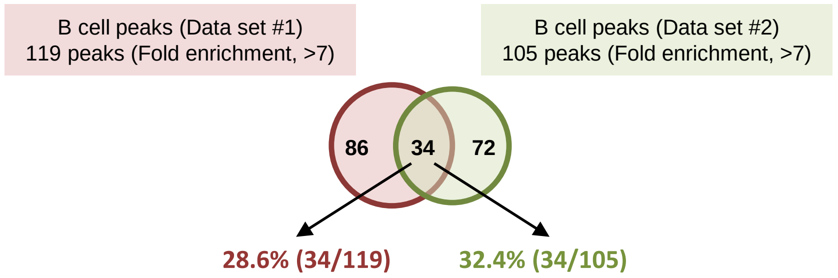

B

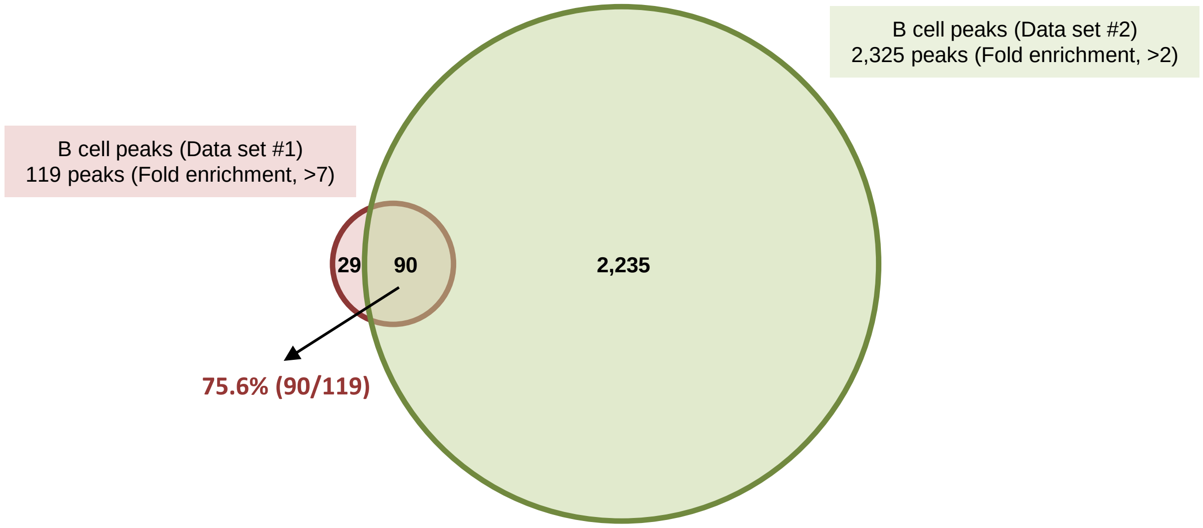

C

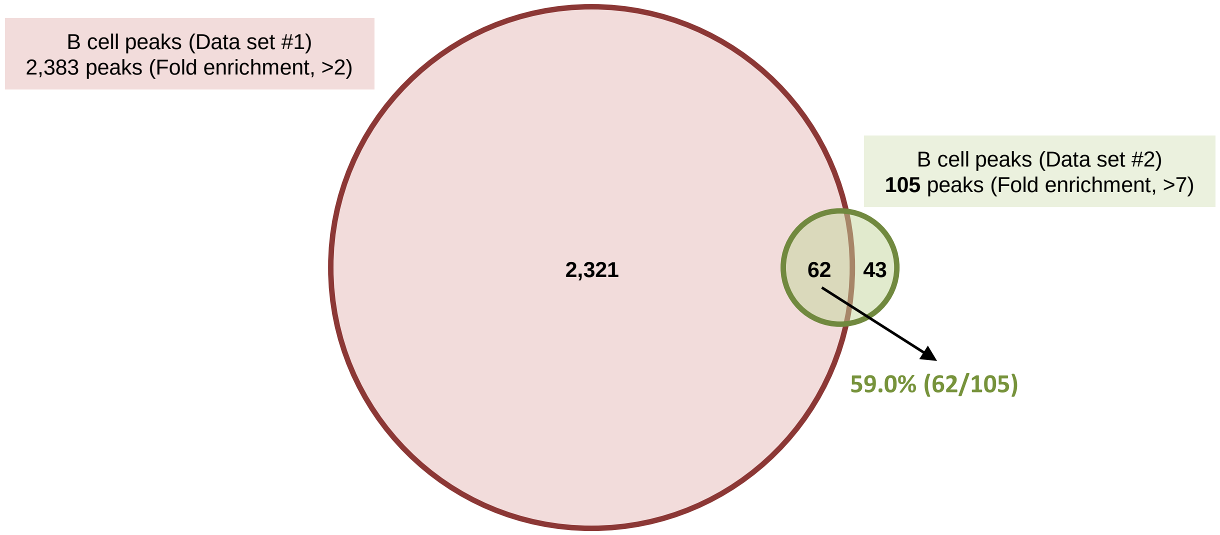

Supplementary Figure S1

A

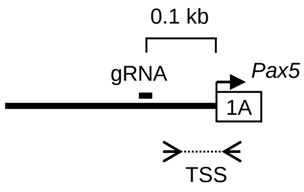

B

CGGCAGGCTCGGGTGCGCCTGGG

C

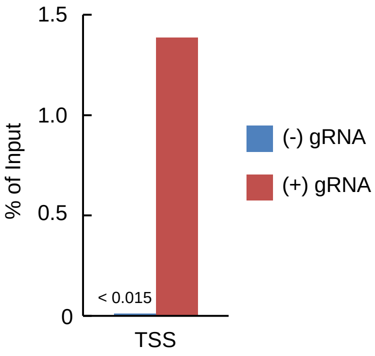

Supplementary Figure S2

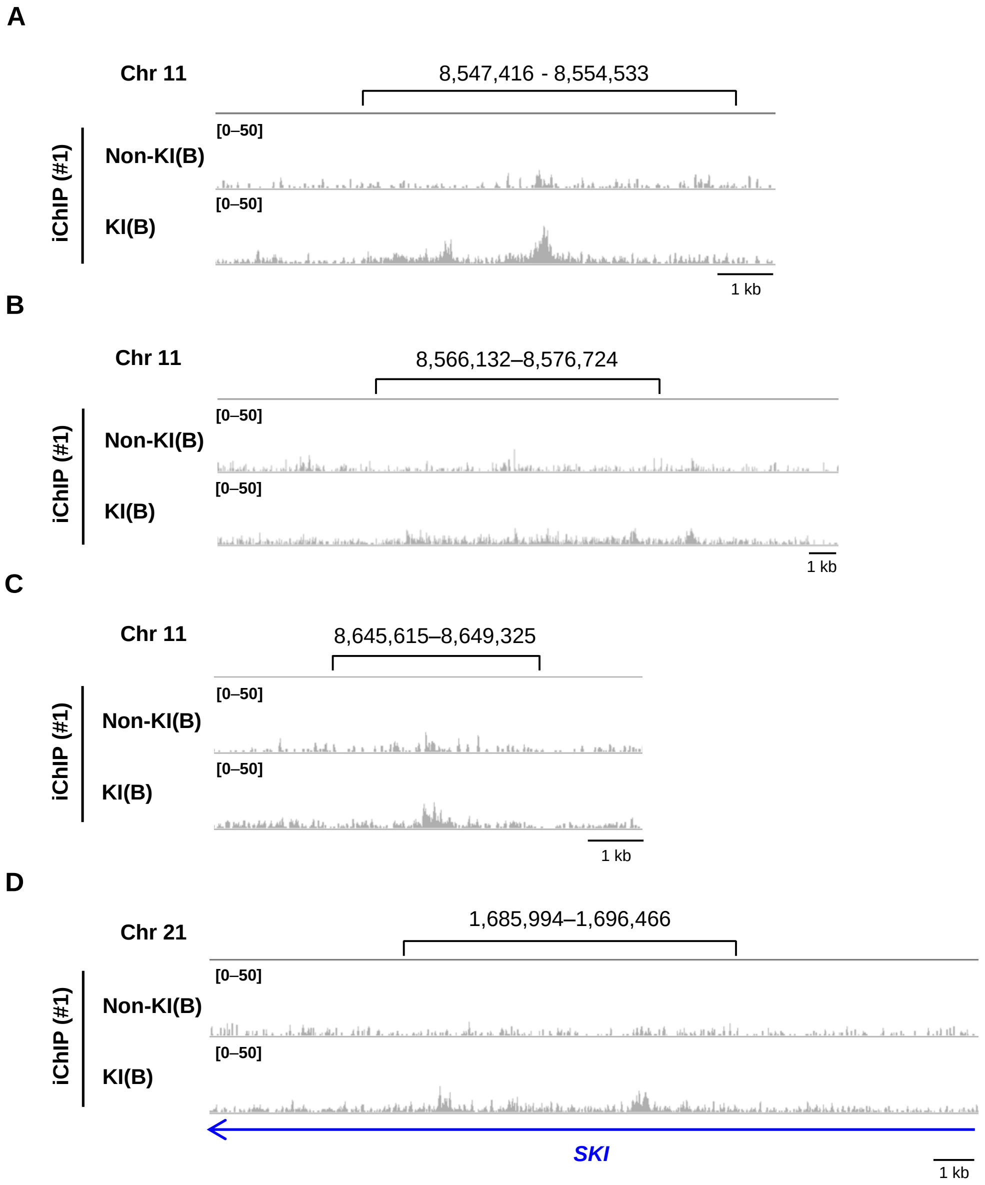

Supplementary Figure S3

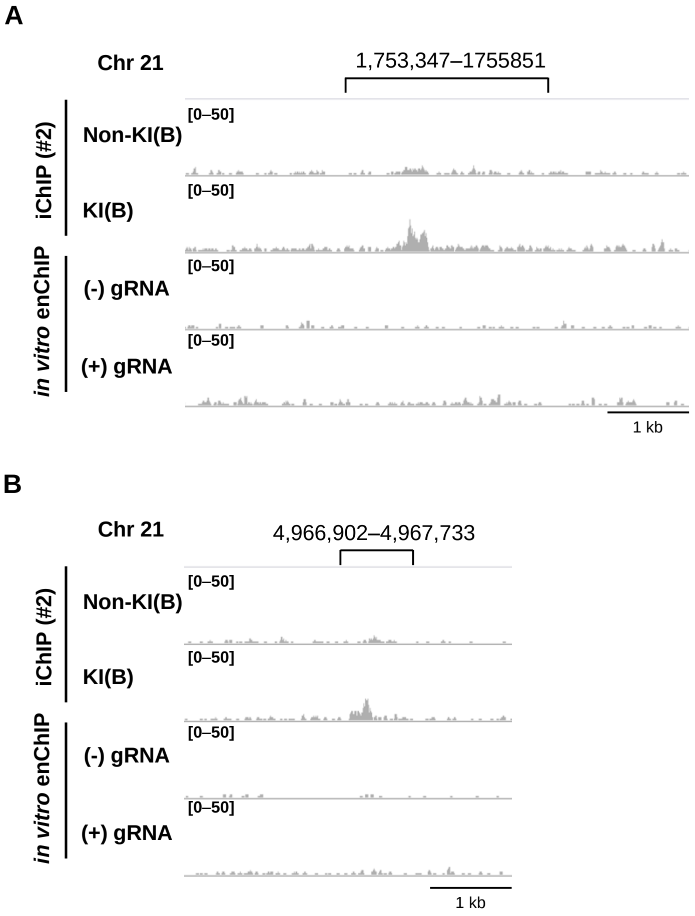

Supplementary Figure S4

sequence	score	mismatches	locus
CGTCAGGCTCTGCTGCGCCTCGG	0.6	3MMs [3:11:13]	chr3:-99566330
CGGGGGGCCCTGGTGCGCCTGGG	0.5	4MMs [4:5:9:11]	chr15:-11125782
TTGCAGGCCCGGCTGCGCCTGGG	0.4	4MMs [1:2:9:13]	chr12:+11477384
TGTCAGGCTCTGCTGCGCCTGGG	0.3	4MMs [1:3:11:13]	chr3:+99518274
CGGCAGCCACGGGTCCGCCTCGG	0.3	3MMs [7:9:15]	chr9:-908349
GGGCAGGATCGCGTGCGCATTGG	0.3	4MMs [1:8:12:19]	chr4:+3876999
GGGCTGTCTCGGGTGGGCCTGGG	0.2	4MMs [1:5:7:16]	chr5:+57943847
CTGCAGGCTCCCGTGCTCCTGAG	0.2	4MMs [2:11:12:17]	chr3:-17761948
GGGCAGGGCCGGGTGAGCCTCAG	0.2	4MMs [1:8:9:16]	chr8:+7479787
AGGCAGGACCGGGTGTGCCTTGG	0.2	4MMs [1:8:9:16]	chr1:-34513890
CCGGAGGCTCCGGTGAGCCTCAG	0.1	4MMs [2:4:11:16]	chrZ:+19851342
CTGCAGGCACGGCTGCACCTCGG	0.1	4MMs [2:9:13:17]	chr24:-1742686
CGGCGGGCGGGGGTGTGCCTAGG	0.1	4MMs [5:9:10:16]	chr2:+85697950
AGGGAGGCTCGGGTGGGCCCGGG	0.1	4MMs [1:4:16:20]	chr2:+115166870
CGGCGTGCTGGGGCGCGCCTAGG	0.1	4MMs [5:6:10:14]	chr21:+671425
CTGCAGGCTGGGGTGCAGCTTGG	0.1	4MMs [2:10:17:18]	chr7:+29178289
CAGCAGGCTCTGGTGCACATGGG	0.1	4MMs [2:11:17:19]	chr9:-22780280
CTGCAGCCTCGAGTGAGCCTCAG	0.1	4MMs [2:7:12:16]	chr5:+44604323
CGGCAGGCAGCGGAGCGCCTCGG	0.1	4MMs [9:10:11:14]	chr18:+5449056
CTGCAGGCTCAGGAGCCCCTTGG	0.1	4MMs [2:11:14:17]	chr23:-3037645
GGGCAGGCTTGGGAGAGCCTGGG	0.0	4MMs [1:10:14:16]	chr27:+1435846
CTGCAGTCTCGGGTGGGACTTAG	0.0	4MMs [2:7:16:18]	chr4:+86154512
CGGCATCCTCGGGCCCGCCTGGG	0.0	4MMs [6:7:14:15]	chr4:-1601108
CGCCAGGCCCGGGAGAGCCTGAG	0.0	4MMs [3:9:14:16]	chr1:-34110060
CGGCAGGCTGGGGTGTGACCAAG	0.0	4MMs [10:16:18:20]	chr8:-21181895
CGGCATGCTCAGGTGTGTCTGAG	0.0	4MMs [6:11:16:18]	chrZ:+55687786
CGGCAGGCTCGGCTGTCCGTGGG	0.0	4MMs [13:16:17:19]	chr11:-1192610
CGGCAGGCTCGGATTCAGCTCGG	0.0	4MMs [13:15:17:18]	chr23:+5009064
CGGCAGGCCCGGGAGCGGGTAAG	0.0	4MMs [9:14:18:19]	chrZ:+9069818

Supplementary Figure S5

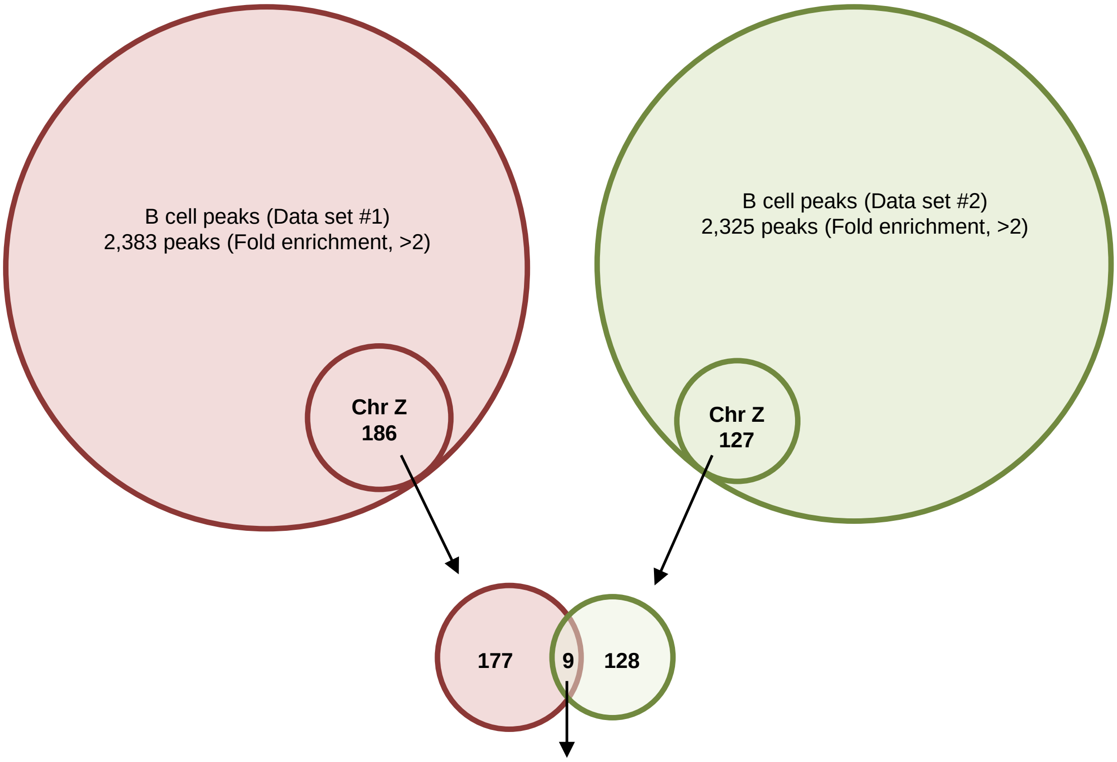

Data set #1								Data set #2								
chr	start	end	length	summit	tags	=-10*LOG10(pvalue)	fold_enrichment	start	end	length	summit	tags	=-10*LOG10(pvalue)	fold_enrichment	fwd	rev
chrZ	331146	332935	1790	1495	103	85.73	4.17	331283	333044	1762	1431	110	55.55	2.77		
chrZ	8943565	8944403	839	303	46	54.81	5.41	8943522	8944983	1462	363	73	52.17	3.77		
chrZ	40367218	40367928	711	517	117	221.15	9.5	40367267	40368168	902	468	162	64.4	6.21		
chrZ	52573289	52574274	986	470	48	106.46	8.9	52573401	52573979	579	332	27	62.3	6.85		
chrZ	65614805	65616234	1430	559	142	69.47	6.07	65614834	65615956	1123	566	105	95.88	5.16		
chrZ	66066227	66070713	4487	1678	289	63.26	3.98	66066776	66070524	3749	1112	286	71.04	3.41		
chrZ	73039200	73040445	1246	503	138	60.37	4.84	73039175	73040354	1180	521	151	57.02	3.68		
chrZ	73405844	73406350	507	315	25	50.95	4.91	73405854	73406498	645	305	25	55.74	7.19		
chrZ	81611786	81613470	1685	1108	597	3100	115.99	81611754	81613591	1838	1161	526	3100	74.37	2090:PAX5:NM_204424,|	

Supplementary Figure S6

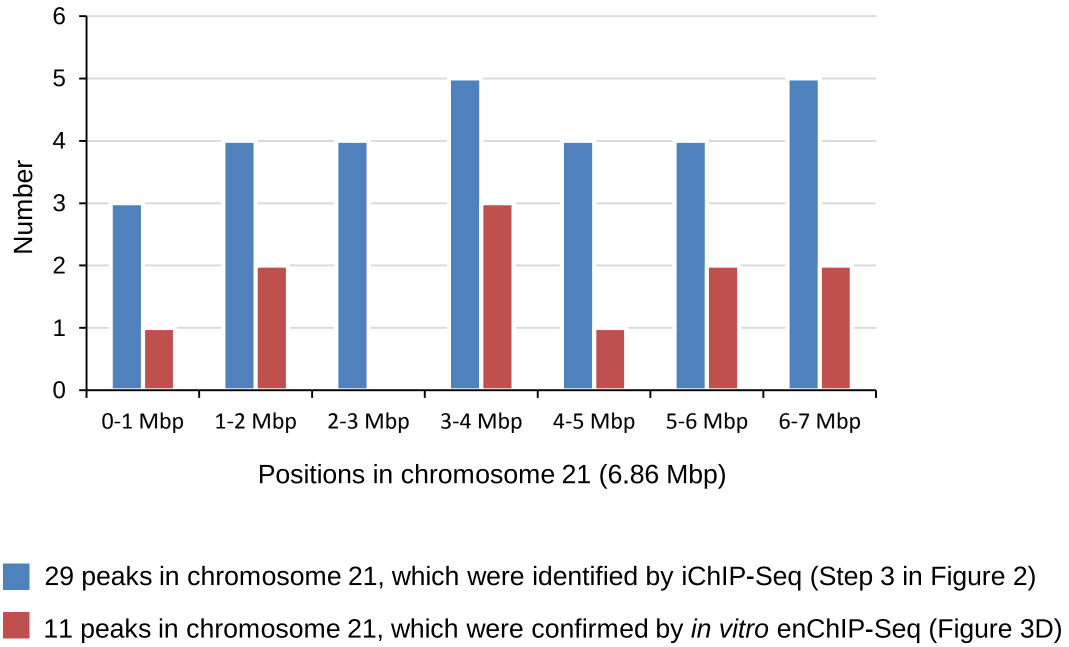

Supplementary Figure S7

LexA BE

caccgcagccactgcagcccccagcccggctccgggcatcagtcgcccttcgcct
ccttctctcggccgtgacacgcagcgcggggaggggtaacggggccgcgcagggg
tcccccgccgccgcagggcaaagtcacgttccgccctcgcggcggggagcggag
aaggggaagtgggcggcaggcgggggcggccgggcgcggccgggctgcgggtgtc
ggcgggcccaaggcgggtccggtccacgcggcggcggagttggtgct**cggcaggc**
tcgggtgcgcctggggacatcctgtgatgctgcagcgcacaggacatgatctcac
acggcgaggggctccgcggcttcttaatcatttcacggttccttcagaggc

-1

Pax5 gRNA

Supplementary Figure S8

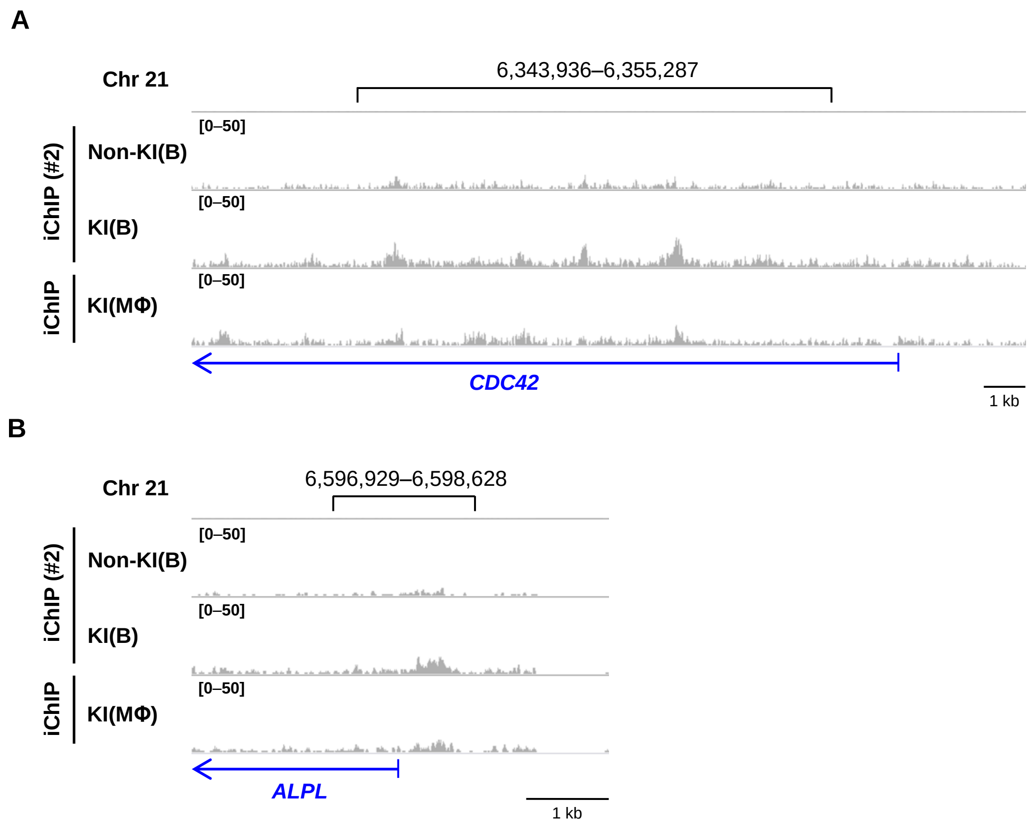

Supplementary Figure S9

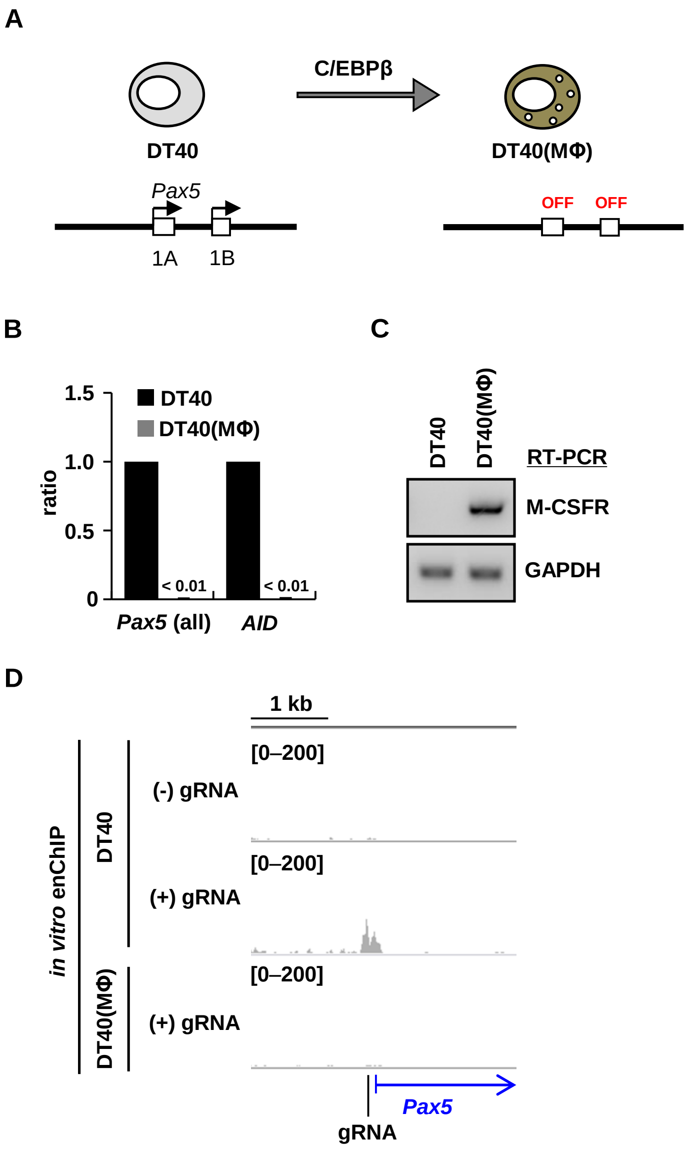

Supplementary Figure S10

A

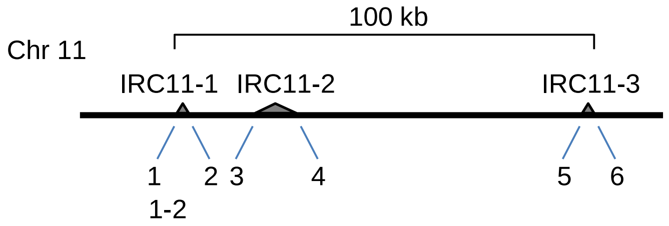

B

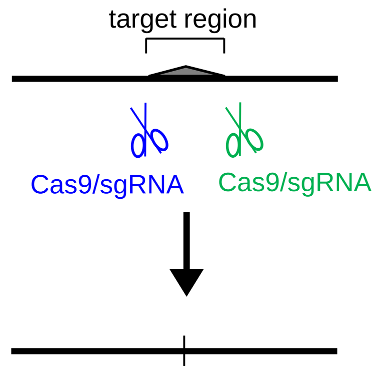

C

Target site	Target sequence (PAM)	sgRNA plasmids
1	GAAAGGCCTGGACCGAGTTGGG	5’ #54
1-2	CACAGCGAGGGGGACGTGAGAGG	5’ #37
2	ACCTTGCTATAGATGGTTGTGG	4.4k 3’ #26
3	ACAGTTCAGATGCAACCCGAGG	10k 5’ #32
4	CCTGGCTACGGACTTTGTCAGG	10k 3’ #10
5	GCAGTTAATCGCATGCCTTTGG	2.5k 5’ #27
6	ACTGTAATAAAGCGTTTAATGG	3’ #29

D

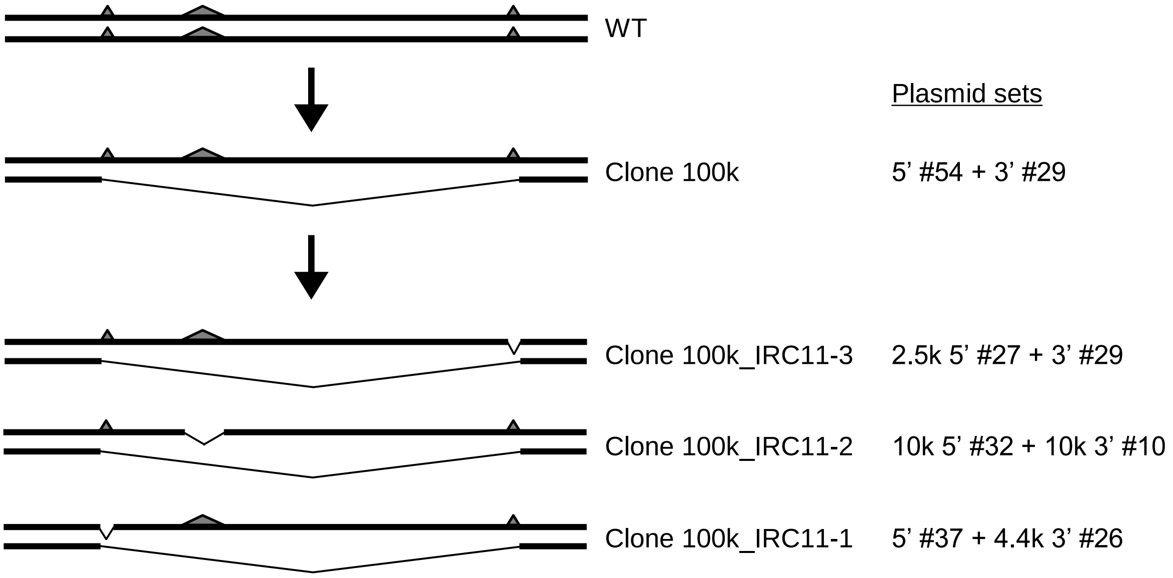

E

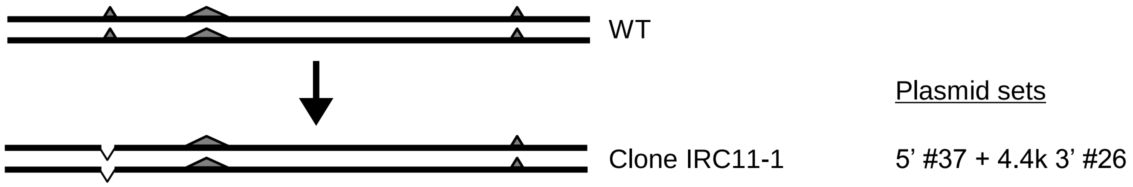

Supplementary Figure S11

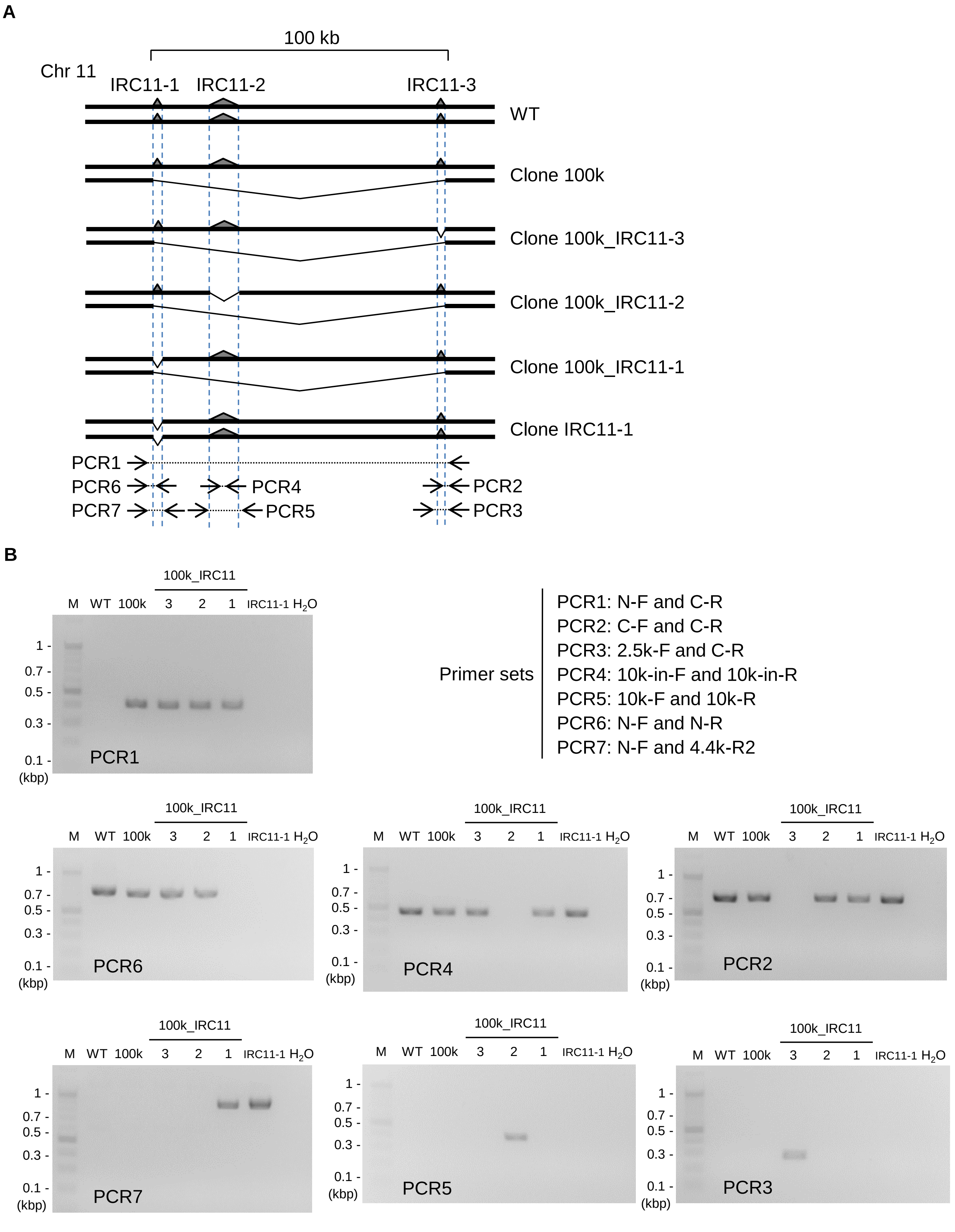

M: DNA ladder marker

Supplementary Figure S12
